# From Haphazard to a Sustainable Normothermic Regional Perfusion Service: A Blueprint for the Introduction of Novel Perfusion Technologies

**DOI:** 10.3389/ti.2022.10493

**Published:** 2022-06-03

**Authors:** Fiona Hunt, Chris J. C. Johnston, Lesley Coutts, Ahmed E. Sherif, Lynsey Farwell, Ben M. Stutchfield, Avi Sewpaul, Andrew Sutherland, Benoy I. Babu, Ian S. Currie, Gabriel C. Oniscu

**Affiliations:** Edinburgh Transplant Centre, Royal Infirmary of Edinburgh, Edinburgh, United Kingdom

**Keywords:** donation after circulatory death, education, normothermic machine perfusion, training model, normothermic regional perfusion, training, simulation

## Abstract

Normothermic Regional Perfusion (NRP) has shown encouraging clinical results. However, translation from an experimental to routine procedure poses several challenges. Herein we describe a model that led to the implementation of NRP into standard clinical practice in our centre following an iterative process of refinement incorporating training, staffing and operative techniques. Using this approach we achieved a four-fold increase in trained surgical staff and a 6-fold increase in competent senior organ preservation practitioners in 12 months, covering 93% of the retrieval calls. We now routinely provide NRP throughout the UK and attended 186 NRP retrievals from which 225 kidneys, 26 pancreases and 61 livers have been transplanted, including 5 that were initially declined by all UK transplant centres. The 61 DCD(NRP) liver transplants undertaken exhibited no primary non-function or ischaemic cholangiopathy with up to 8 years of follow-up. This approach also enabled successful implementation of ex situ normothermic liver perfusion which together with NRP contributed 37.5% of liver transplant activity in 2021. Perfusion technologies (*in situ* and *ex situ*) are now supported by a team of Advanced Perfusion and Organ Preservation Specialists. The introduction of novel perfusion technologies into routine clinical practice presents significant challenges but can be greatly facilitated by developing a specific role of Advanced Perfusion and Organ Preservation Specialist supported by a robust education, training and recruitment programme.

## GRAPHICAL ABSTRACT

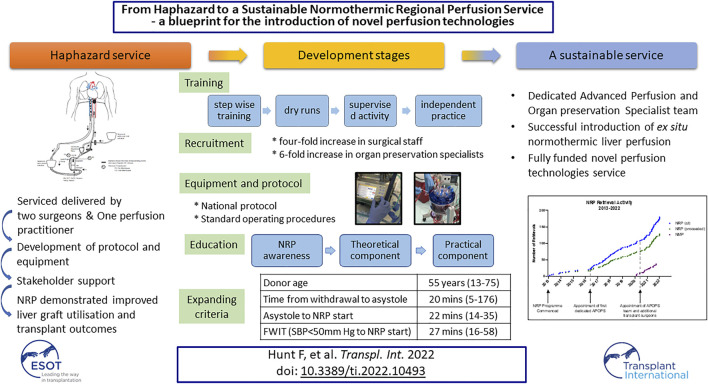

## Introduction

Donation after circulatory death is now accepted in many countries across the globe. The confines of widely varying regulatory arrangements lead to significant variation in clinical practice with regards to the duration of the “no touch” period after asystole (from 2 min in some areas of the US though to 20 min in Italy) and the use of ante-mortem interventions such as heparinisation and femoral vessel cannulation (1).

In the United Kingdom, donation after circulatory death (DCD) currently accounts for 43% of all deceased organ donors (2). DCD donation has had a considerable impact on kidney transplantation in the UK with utilisation rates comparable with DBD donation and very good clinical outcomes (1). In contrast, only a minority of DCD livers are utilised, and, as a result, DCD liver transplantation contributed to only 16.2% of all deceased donor transplants in the UK in 2020-21 (3). Despite a selective approach, including minimisation of cold ischaemic time and careful recipient selection, the burden of additional complications specific to DCD liver transplantation is significant. Specifically, higher rates of early allograft dysfunction in the short term and multi-focal biliary stricturing (ischaemic cholangiopathy) in the long term lead to considerable morbidity and result in a retransplantation rate of nearly 25% in the recent UK experience (4). Similarly, the rates of DCD pancreas utilisation are low, with increased complication rates (5) but good long-term clinical outcomes (6).

In recent years, several approaches have been adopted to mitigate the complications of DCD organ transplantation. These range in ambition and complexity from optimisation of the retrieval process (7), through the use of novel perfusion and preservation technology, in the form of *ex situ* machine perfusion [hypothermic (8, 9), oxygenated hypothermic (10, 11), or normothermic (12)], or by re-establishing an oxygenated blood supply to donor organs *in situ* after donor asystole with Normothermic Regional Perfusion (NRP) (4, 13) or a combination of these strategies (14). *Ex situ* technologies have shown a variable degree of benefit in DCD transplantation in terms of either prolonged preservation, a reduction in the rate of complications, immunomodulation, and improved graft outcomes. In contrast, the use of *in situ* NRP offers several advantages over these approaches, including a beneficial impact for all abdominal organs with a single intervention (15).

Novel perfusion and preservation technologies are an exciting innovation that so far has been driven by enthusiasts who have designed the technology, refined the protocols, and generated the initial outcome data. However, translation into routine clinical use is more complex and requires adequate and continuous evaluation (16), an appropriate skill mix in the team, training, and education as well as institutional support for innovation and adequate funding. The absence of such an environment has often been cited as a reason for the lack of adoption (17). Furthermore, the individual technology complexity and the magnitude of change required to the existing practices play important roles when considering their adoption. There is no doubt that the use of NRP is at the higher end of technological complexity, as it requires supplementation of skills as well as additional equipment to travel to the donor centre with a modified Extracorporeal Membrane Oxygenation (ECMO) device. For this reason, logistical feasibility has been cited as a barrier to NRP implementation into clinical practice by many transplant teams.

Herein, we describe the Edinburgh Transplant Centre’s experience in implementing NRP from a haphazard use to a routine and sustainable clinical service. Whilst the focus here is on NRP specifically, the approach described can act as a framework for the introduction of other novel technologies more widely.

## Normothermic Regional Perfusion—The Exploratory Stage

NRP was first described in Spain in the context of unexpected cardiac arrest, with potential donors commenced on Extra-Corporeal Membrane Oxygenation (ECMO) *via* femoral vessel cannulation whilst CPR was ongoing (18). In the context of unexpected cardiac arrest, NRP presents effectively the only viable solution for proceeding to successful solid organ donation [“uncontrolled” or Maastricht Category II DCD donation (19)] and transplantation. This approach was subsequently expanded to include all DCD donors and has since been considered in several European countries.

The cumulative insults of the initial warm ischaemic time followed by ongoing ischaemia during static cold storage affects all DCD organs to various degrees. The liver is particularly sensitive, as reflected in higher rates of early allograft dysfunction and late ischaemic biliary stricturing (ischaemic cholangiopathy) compared to livers transplanted from DBD donors. Accordingly, in the context of Category III DCD, the use of NRP was driven primarily by a need to improve liver graft utilisation and outcomes.

To assess the role of NRP with regard to the liver outcomes, the procedure was first introduced in the UK in 2011-2012 as part of a research study (Cambridge), to support the development of a pilot of uncontrolled DCD donation (Edinburgh) (20) and as a service development in Birmingham. The initial combined report from the three centres demonstrated a reduction in early allograft liver dysfunction, a complete absence of ischaemic cholangiopathy, and hinted at an improved function for the renal transplants undertaken following NRP (21). Based on these encouraging data, NRP provision continued as part of a formal service evaluation project in two centres (Edinburgh and Cambridge) overseen by NHS Blood and Transplant (NHSBT) to enable further data acquisition whilst allowing for refinement of the surgical procedure and technology as well as considering how the NRP service could be configured and integrated into the established UK national retrieval service (22) to build a robust business case for funding.

## Normothermic Regional Perfusion—The Development Stage

The development phase had several key targets, including promotion and wider acceptance, refinement of the protocol, technique and equipment, and defining the standard operating procedure, and it involved a formal four-step process for implementation: recruitment, education and training, implementation, and review for evaluation.

### Stakeholders Support

Successful implementation of NRP involves many stakeholders including transplant co-ordinators, specialist nurses in organ donation and donor hospital staff, patients, and transplant surgeons for all organs at a national level, given the current allocation protocols in place in the United Kingdom. The plans and proposed approach were discussed extensively and support was gained from stakeholders groups at the local and regional levels to facilitate logistics, deployment, and operating room planning in the referring hospitals. At the national level, discussions with NHS Blood and Transplant and organ-specific advisory groups ensured governance support and organ acceptance. A wider consultation about the ethical implications of NRP took place and several technical modifications were put in place to ensure that the use of NRP does not invalidate the circulatory determination of death and does not lead to accidental brain perfusion (23), thus addressing issues that may preclude its adoption in other countries (24).

### Recruitment and Definition of Roles in the Team

One of the key points in ensuring sustainability was the definition of the roles in the team. Our standard retrieval team comprises a lead surgeon and an assistant surgeon, a scrub practitioner, and a theatre practitioner for cold perfusion. During the exploratory stage, it became clear that the additional tasks needing completion during NRP combined with the management of the pump will require additional manpower. To that extent, a new role of Advanced Perfusion and Organ Preservation Specialist (APOPS) with the knowledge and skills to manage all aspects of machine perfusion and the NRP process was created. APOPS travel to the donor centre with the standard retrieval team, manage the pump during perfusion and coordinate the NRP process (blood sample processing, data recording, and allocation of roles prior to the start of the procedure to ensure a rapid setup in theatre). The team of five routinely travels within the standard logistical footprint of a regular retrieval team in a single vehicle or aircraft anywhere in the United Kingdom.

**FIGURE 1:**
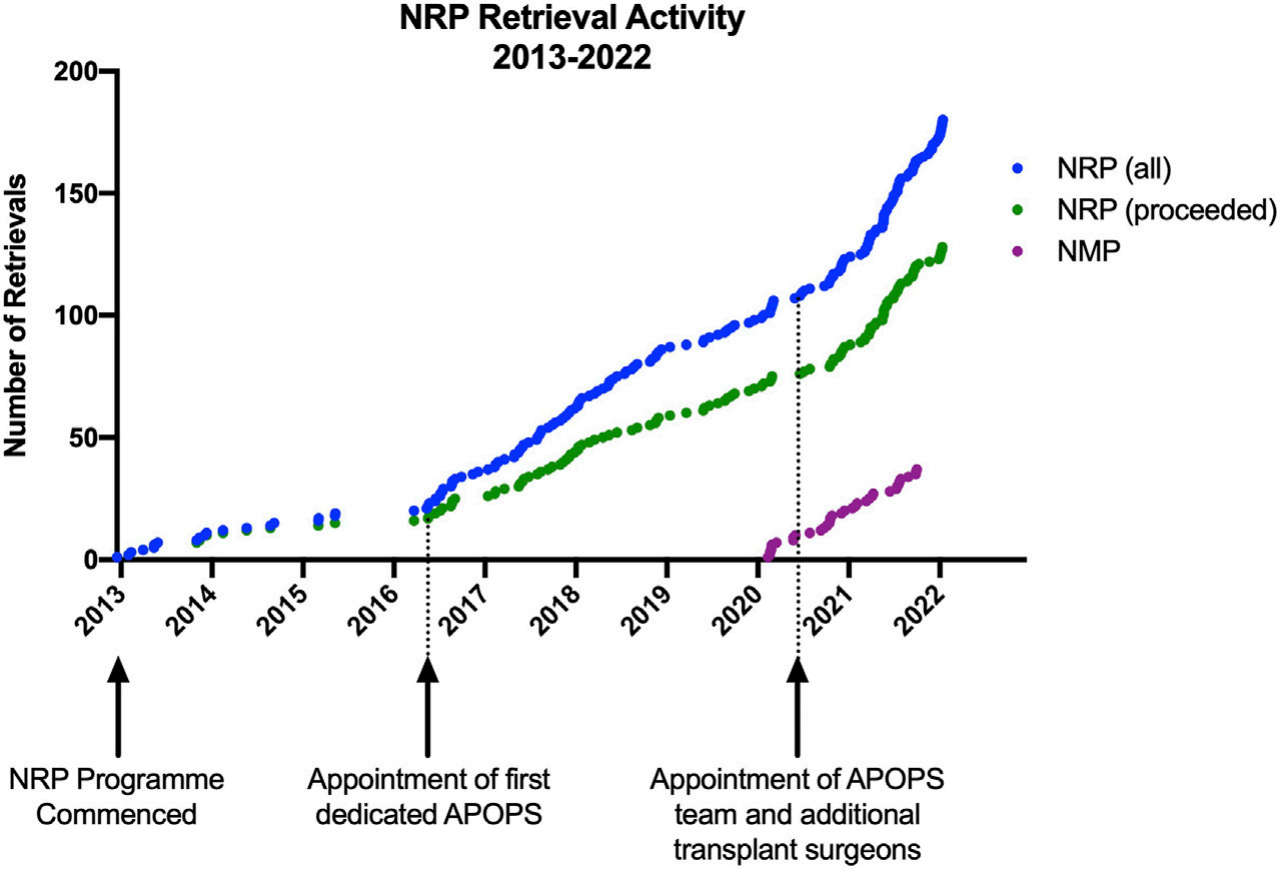
Edinburgh normothermic regional perfusion and *ex situ* normothermic machine perfusion activity and key points in the evolution of the programme.

The development of the APOPS role also had an instrumental impact on the expansion of the NRP programme within our service (Figure 1) by coordinating logistics, setting up training and education, and overseeing the governance of the equipment.

**FIGURE 2:**
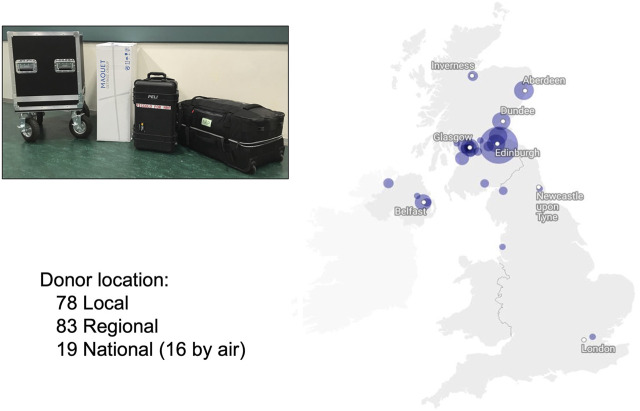
Distribution of NRP retrievals by location and footprint of standard NRP retrieval equipment. (Map by Datawrapper^©^) (locally defined as Edinburgh; Regional defined as Scotland; National defined as the rest of the United Kingdom).

Concomitantly with the development of the APOPS team, additional surgeons were recruited to undertake NRP (six currently) to ensure robust service delivery and a sustainable rota, which allowed us to increase the reach of NRP within and outwith our allocated retrieval area (Figure 2).

### Equipment and Surgical Procedure

**FIGURE 3:**
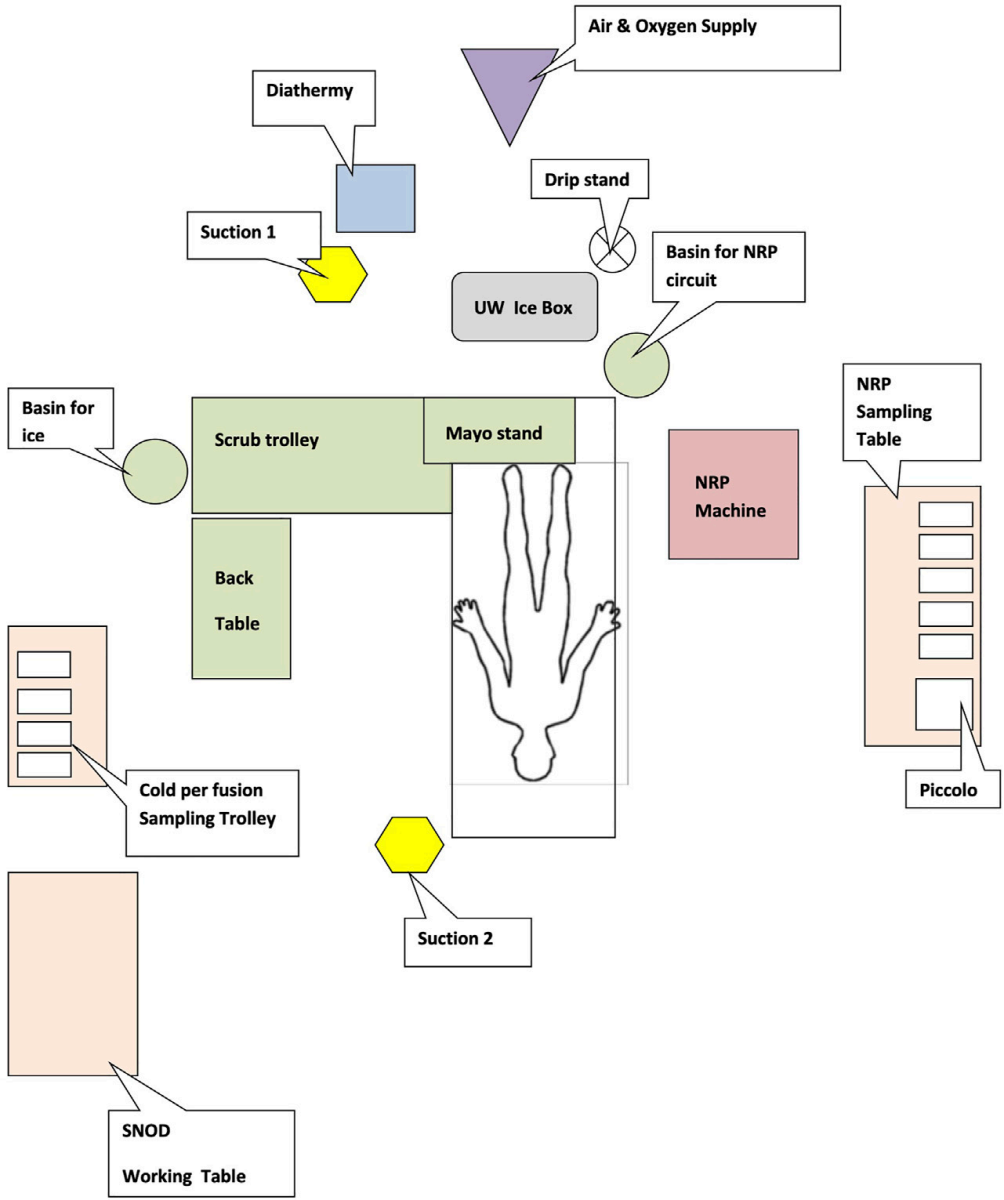
NRP theatre setup (floor map).

The initial equipment included a Medtronic pump, a Maquet heat exchanger, and a complex bespoke circuit that had two bypass loops to mitigate against potential problems such as clots in the reservoir or the leukocyte filter. Biochemical and blood gas analyses were undertaken in the local hospitals. However, this limited portability and presented logistic challenges with regional and national donors and lengthen the response time to biochemical interpretation and decision making. As a result, the equipment was minimised to allow portability and ease of use and currently consists of a Maquet Cardiohelp^®^ device, a heat exchanger, and a simplified circuit (Maquet®– BE-MECC 50312 Edinburgh NRP pack) (Figure 3). Point of care devices are used for biochemical (Abbott Picolo^®^) and blood gas analysis (I-Stat^®^) to provide real-time decisions and make the team fully self-sufficient.

In terms of surgical approach, initially, minimal changes were made to a standard DCD procurement to enable adoption, with aortic (24Fr DLP^®^ Single Stage Cannula, Medtronic Inc, Minneapolis, MN, United States) and inferior vena cava cannulation (29/37Fr Edwards Trim-Flex™ Dual Stage Venous Cannula, Edwards Lifesciences LLC, Irvine, CA, United States), and descended aorta occlusion using an intra-aortic balloon (Coda^®^ LP Balloon Catheter, Cook Medical Inc, Bloomington, IN, United States) as previously described (21). Recently we have adapted the surgical cannulation to the donor situation using femoral cannulation if there is a hostile abdomen (i.e., extensive previous surgery) or if there is concomitant cardio-thoracic retrieval. If cardiothoracic organs are to be retrieved, the intention to undertake NRP is discussed with the cardiothoracic team as early as possible and the skill mix of the on-call retrieval teams is reviewed, with the provision of additional support if needed. To prevent inadvertent brain perfusion (which is unlikely in abdominal NRP as long as complete occlusion of the descending aorta is achieved) we added a safeguard that involves the insertion of a large cannula (9Fr DLP^®^ Aortic Root Cannula, Medtronic Inc, Minneapolis, MN, United States) in the ascending aorta that is left open to atmosphere before A-NRP is commenced (23).

### Protocol and Standard Operating Procedures

Over the last 9 years, a comprehensive NRP protocol and standard operating procedures have been developed and refined to incorporate all aspects of the process, including pre-departure equipment checklists, quick reference cards for equipment set-up, and troubleshooting potential problems during perfusion. An overview of the process is outlined below, and the entire operating procedures and relevant material are included in Supplementary Appendix SA.

Upon arrival at the donor hospital the APOPS set up the theatre as illustrated in Figure 3 (often repositioning the operating table to facilitate access to air and oxygen supplies for the NRP machine) before setting up the NRP machine and point of care blood gas and biochemistry analysers (iStat^®^ and Piccolo^®^) whilst the surgeons take a handover from the Specialist Nurse in Organ Donation (SNOD) and check through relevant donor documentation.

**FIGURE 4:**
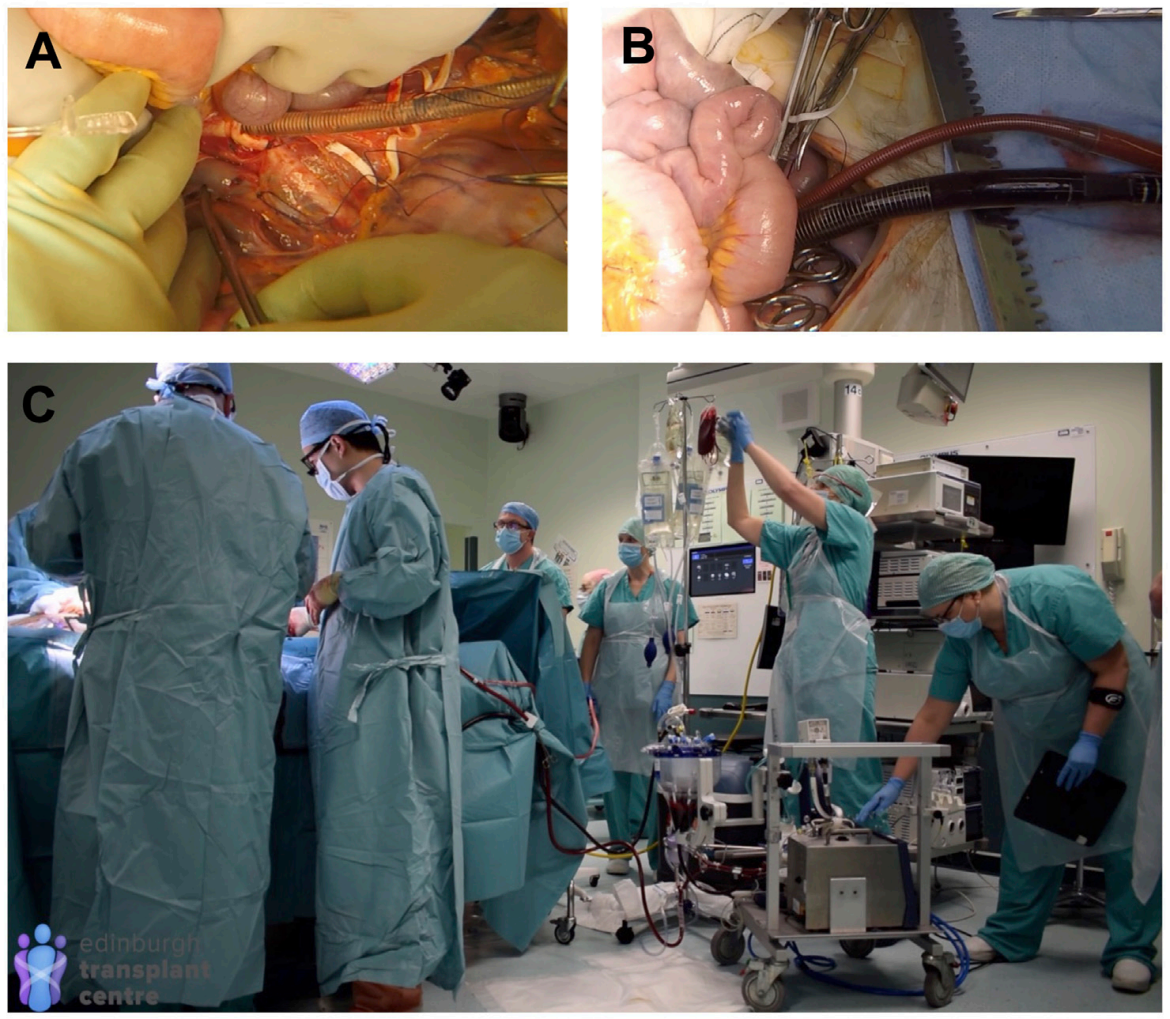
NRP Technique (see also full protocol and operative video demonstration in Supplementary Appendix). **(A)** cannulation of the abdominal aorta and IVC; **(B)** NRP circulation established; **(C)** theatre arrangement shortly after commencing NRP circulation (all operative images obtained with donor family permission).

The entire team (retrieval team, SNOD, local theatre staff, and intensive care doctor) then join together for a detailed briefing to include all aspects of the process, timings, communication with recipient transplant teams, and what to expect for those who have not been previously involved in an NRP retrieval (e.g., awareness of small bowel peristalsis). The APOPS will then set and prime the disposable NRP circuit whilst the surgeons prepare all antibiotics and drugs required for addition to the perfusate solution. The entire set-up can be completed within 30 min, minimising any additional time required before withdrawal of life-sustaining treatment from the donor. After asystole and the legally mandated “stand off” period, the donor is transferred into the operating theatre. Rapid laparotomy and a limited Cattel-Braasch manoeuvre are undertaken to allow access to the abdominal aorta and vena cava, which are then cannulated with 26F and 29F cannulas respectively and connected to the NRP circuit (Figure 4). A median sternotomy is carried out, the descending thoracic aorta is cross-clamped and a cannula is placed in the aorta arch and left open to air to confirm the complete absence of any circulation to the brain. At this point (usually 7–10 min after knife to skin), tubing clamps are released and NRP circulation commenced. Any bleeding points in the abdomen or chest are secured whilst continuously communicating with the APOPS managing the NRP pump until a stable circulation is achieved. After 5–10 min of stable circulation, a T-tube is placed in the bile duct (to measure bile production) and no/minimal further dissection is undertaken during the 2 h of NRP circulation to minimise haemodynamic disturbance and allow time to focus on dynamic functional organ assessment. Biochemistry and blood gas samples are taken every 30 min for the 2-hour duration of the NRP after which retrieval proceeds in a similar manner to DBD donation (21).

### Education and Training

Mastering all aspects of NRP DCD organ retrieval, pump management, and organ assessment required training to ensure repeatability and routine. An education programme was designed involving three key components: an NRP awareness session followed by theoretical and practical components (Table 1). Training sessions were delivered initially on a daily basis focusing on the practical aspects of NRP and evolved as the programme expanded with regular group/team sessions, weekly drop-in sessions, tailored one to one meetings depending on staff availability and level of skill and knowledge and fully simulated retrievals using bespoke mannequins developed in-house. The NRP awareness sessions were instrumental in creating wider support and were open to all stakeholders in the donation, retrieval, and transplant process. These sessions covered the need for NRP, the known benefits for transplanted organs, and the practicalities of how NRP is undertaken (Table 1).

**TABLE 1:** Normothermic regional perfusion training programme structure and content (APOPS = Advanced Perfusion and Organ Preservation Specialist; SNOD = Specialist Nurse in Organ Donation; ICU = Intensive Care Unit).

Training session	Content	Attendees	Session approach
NRP Awareness	• What is the need?	• Surgeons	• Drop-in
	• What is NRP?	• APOPS	• Team session
	• Why NRP?	• Theatre team	• Seminars
	• Benefits of NRP?	Scrub	
	• Outcomes	Cold preservation	
	• Simulation of NRP Retrieval	• SNODs	
	Theatre set-up	• Recipient Coordinators	
	Practical demonstration	• Blood bank staff	
		• Donor hospital team	
		ICU team	
		Theatre staff	
Theoretical Component	• Anatomy	• Surgeons	• One:one
	• Physiology	• APOPS	• Team session
	What happens in cells during DBD	• Theatre team	• Interactive case-based discussion
	What happens in cells during DCD	Scrub	
	Impact of DCD on organ function	Cold preservation	
	What happens in cells when using	• SNODs	
	NRP		
	• Equipment configuration and circuit dynamics		
Practical Component	• Pre–retrieval setup	• Surgeons	• One:one
	• Composition of Priming solution	• APOPS	• Team session
	• Surgical Protocol/Cannulation	• Theatre team	• Video debrief
	• Pump/ Circuit Training	Scrub	• Case-based discussion
	• Troubleshooting	Cold preservation	• Focus tutorials
	• Blood Sampling/Blood Analysers	• SNODs	
	• Interpretation of Blood Results		
	• Communication		
	• Paperwork/documentation		
	• Simulation		

**FIGURE 5:**
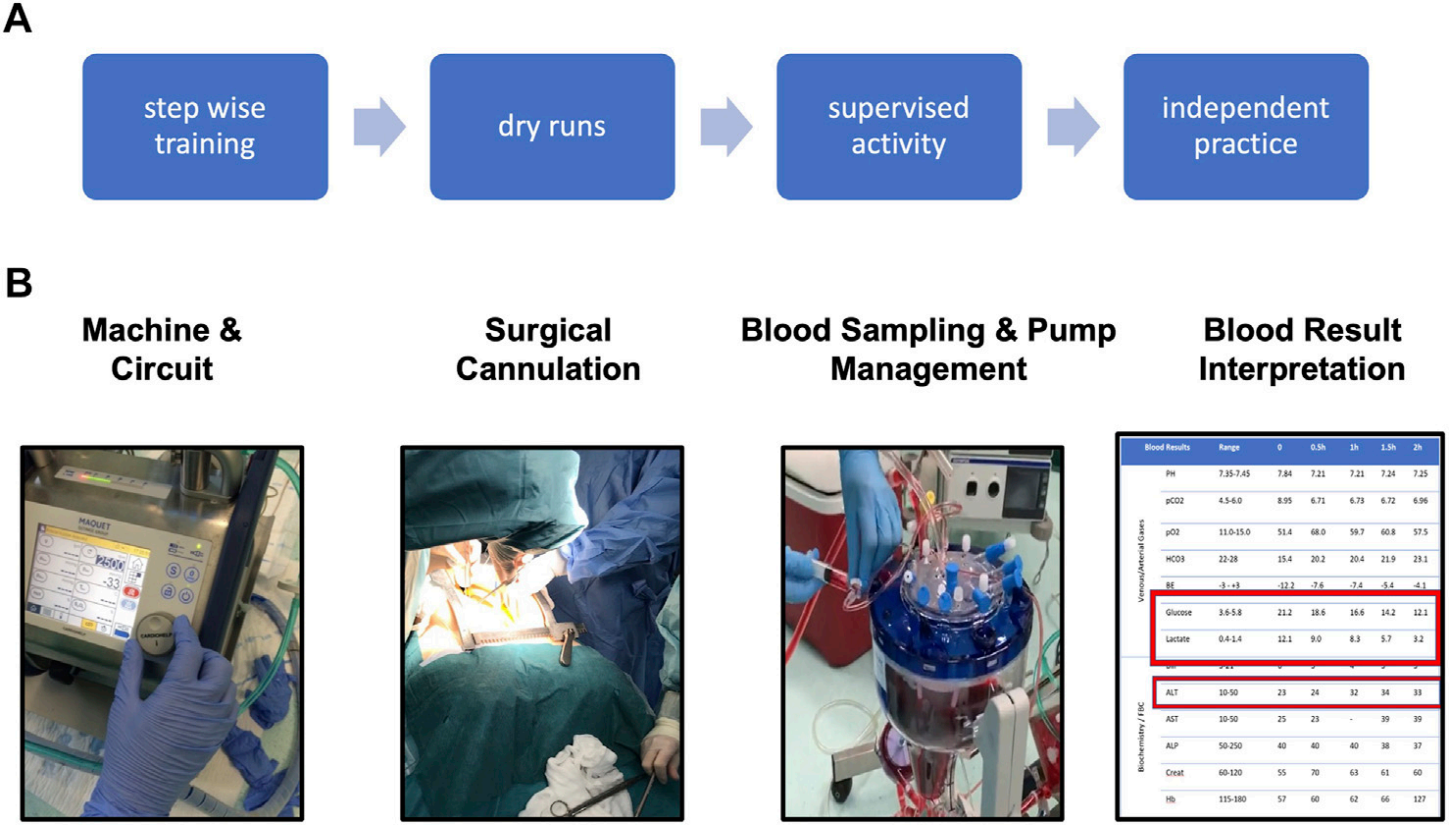
NRP training. **(A)** overview of training strategy. **(B)** Fundamentals of NRP knowledge and skills required for independent practice.

Theory covered the fundamentals of physiology and basic scientific principles underpinning the effects of organ ischaemia during the retrieval process and how this can be mitigated with NRP. Practical sessions were designed to teach the technical skills of all aspects required to establish and maintain NRP (from machine setup, through surgical technique, to blood result interpretation) and were delivered in tasks specific workstations. Dry-runs, which included troubleshooting scenarios, were considered essential for all core retrieval team members prior to actual donor attendance (Figure 5).

Training was delivered by the APOPS with support from clinicians and technical experts from the companies providing the NRP equipment.

A comprehensive competency framework (Supplementary Appendix SA) was designed to guide trainees safely through the detailed aspects of the process; this acted as a record of achievement of knowledge, skills, and competencies, ensuring accountability. A training pack (Supplementary Appendix SA) was also developed to support the training sessions and as a practical guide directing the learner through the entire NRP retrieval process in a logical way.

Regular team debriefings include high-definition intraoperative videos, which provide invaluable material for teaching, particularly after technically challenging donors, and focused tutorials on historical donor cases complementing the accumulating experience on organ retrievals runs.

Whilst the road to achieving independence in NRP practice varies greatly and depends on several factors including previous experience with standard DCD retrievals, following this training programme most of our senior trainees became comfortable running NRP after five mentored cases.

## Results and Current Status

**FIGURE 6:**
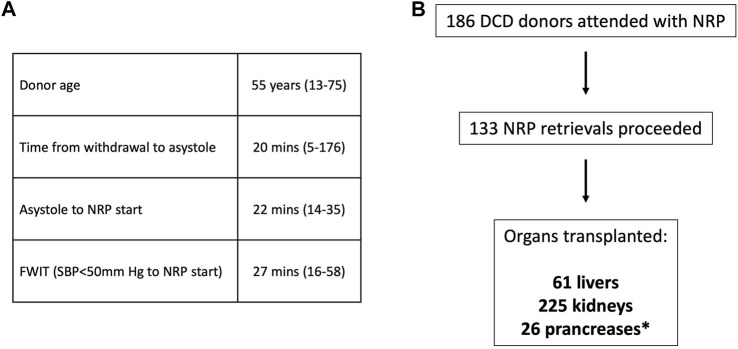
NRP retrieval outcomes. **(A)** Donor and retrieval parameters (median, range); FWIT, functional warm ischaemia time (SBP- systolic blood pressure). **(B)** Breakdown of organs successfully transplanted from NRP donor retrievals (*–solid pancreas transplants).

Over the last 9 years, our team has attended 186 DCD donors with NRP. A total of 133 donors progressed to donation, resulting in 61 liver transplants, 225 kidney transplants, and 26 pancreas transplants (Figure 6). Changes in the team structure outlined above have allowed for a considerable increase in capacity, reaching 93% NRP cover for all DCD donors attended by our team anywhere in the United Kingdom. The functional warm ischaemic time has been extended to 1 h and the time from withdrawal to asystole to 3 h (Figure 6) with no detrimental effects. All livers transplanted (52 locally and 9 in other centres) remain free of ischaemic biliary complications (4). A total of five livers that were declined by all UK centres based on donor history prior to retrieval were rescued and transplanted following assessment during NRP; this led to a change in acceptance policy and we only decline livers after functional assessment during NRP. The criteria for accepting a DCD NRP liver for transplantation have evolved with time and currently are based on a composite index of liver function tests (ALT less than 500–600 iu/L), lactate (downward trend), glucose (evidence of consumption), pH (normalisation), as detailed in the attached national protocol (Supplementary Appendix SA). Livers where ALT >1000 iu/L or lactate is increasing or not falling are usually discarded whilst for those in-between *ex situ* NMP is used for additional functional assessment.

**FIGURE 7:**
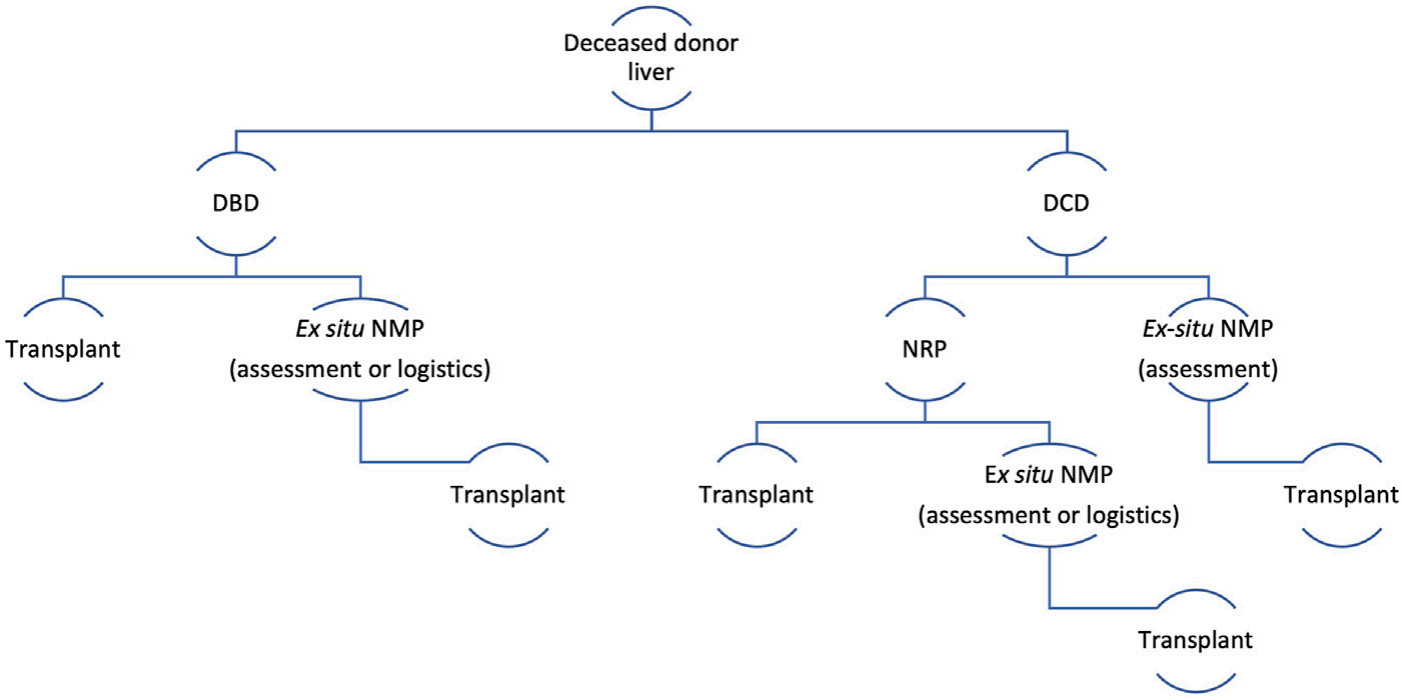
Perfusion and preservation strategy for deceased donor liver transplantation. *Ex situ* NMP, *Ex situ* Normothermic machine perfusion (back at base model); DBD, donation after brain death; DCD, donation after circulatory death.

Using NRP transferable skills and employing the strategy outlined above, we successfully implemented *ex situ* Normothermic Machine Perfusion using the OrganOx *Metra®* in clinical practice in 2020 and undertook 41 liver perfusions and transplanted 27 grafts. The donor and recipient demographics and preservation times for the NMP programme are illustrated in Table 2. All grafts are monitored and managed on the device by the APOPS. Our current perfusion and preservation strategy is detailed in Figure 7. Essentially, all DCD donors that we attend undergo NRP. Additional use of *ex situ* NMP is indicated if further functional assessment is required or if there are logistics or complex recipient issues. If a DCD liver is retrieved without NRP by a different team, these organs will undergo *ex situ* NMP at base for functional assessment prior to transplantation. The use of *ex situ* NMP for DBD livers is driven by logistics or if a functional assessment is needed for marginal grafts.

**TABLE 2:** Demographic data, indications, and preservation times for *ex situ* normothermic machine perfusion.

	All Perfusions (*n* = 41)	Livers Transplanted (*n* = 27)	Livers Not Transplanted (*n* = 14)
Donor demographics
Gender M:F	20:21	12:15	8:6
Age median (range)	52 (15–77)	51 (15–71)	58.5 (21–77)
BMI median (range)	26.5 (19.7–37.0)	26.1 (20.0–36.3)	27.8 (19.7–37.0)
Cause of death
Hypoxic Brain	15	10	5
Intracerebral haemorrhage	21	13	8
Intracerebral thrombosis	2	2	0
Meningitis	1	1	0
Trauma	2	1	1
Donor type
DBD	32	20	12
DCD	1	0	1
DCD/NRP	8	7	1
Indication for *ex situ* NMP
Further Assessment	10	5	5
Logistics	26	20	6
Complex Recipient	5	2	3
Preservation time (min)
CIT (1st)	453	470	415
Normothermic machine preservation time	508	621	297
CIT (2nd)		22	—
Total preservation time		1113	712
Recipient demographics
Gender M:F		22:5	
Age median (range)		58 (24–71)	
BMI median (range)		27 (20–44)	
UKELD median (range)		53.5 (45–74)	
Indication
ALD		8
HCC*		9
HCV		1
NAFLD		6
PBC		2
PSC		4
Cryptogenic cirrhosis		1

BMI, body mass index; DBD, donation after brain death; DCD, donation after circulatory death; NRP, normothermic regional perfusion; CIT(1st), Cold ischaemic time from *in situ* cold perfusion to liver perfusion on device; CIT (2nd), cold ischaemic time from liver disconnected from device to reperfusion in recipient; UKELD, UK model for end stage liver disease; HCV- hepatitis C; ALD, alcoholic liver disease; HCC, hepatocellular carcinoma; NAFLD, non-alcoholic fatty liver disease; PBC, primary biliary cirrhosis; PSC, primary Sclerosing cholangitis; *–HCC cases as primary indication or in association with other liver disease.

**FIGURE 8:**
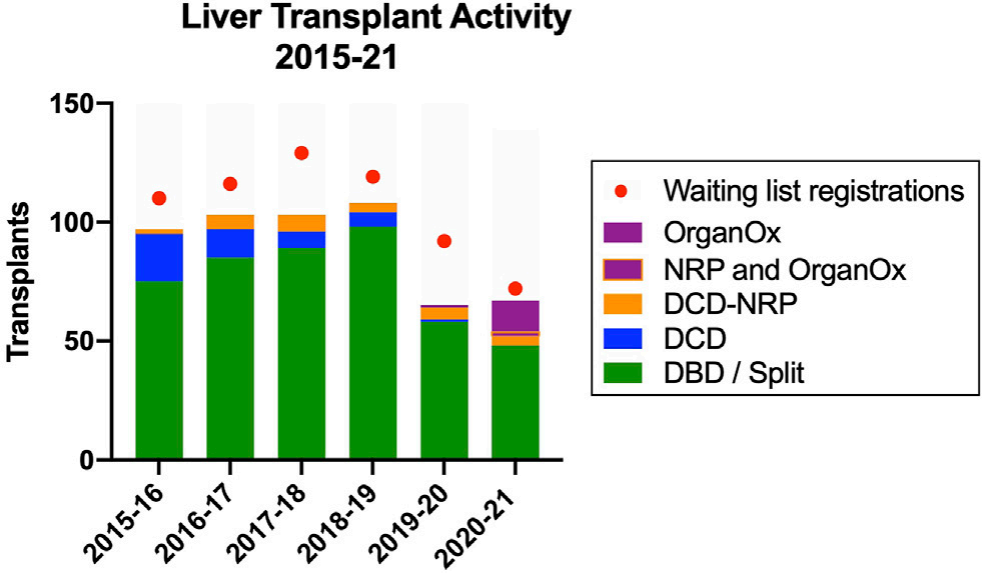
Impact of NRP and *ex situ* NMP on liver transplant activity in the Edinburgh Transplant Centre.

The use of these two technologies had a significant impact on the liver transplant activity mitigating a considerable reduction in liver transplant activity in the wake of the introduction of the new UK National Liver Offering System for DBD grafts (25) and the COVID-19 pandemic and represented 37.5% of activity in 2021 (Figure 8).

### Funding a Novel Perfusion Technologies Programme

**FIGURE 9:**
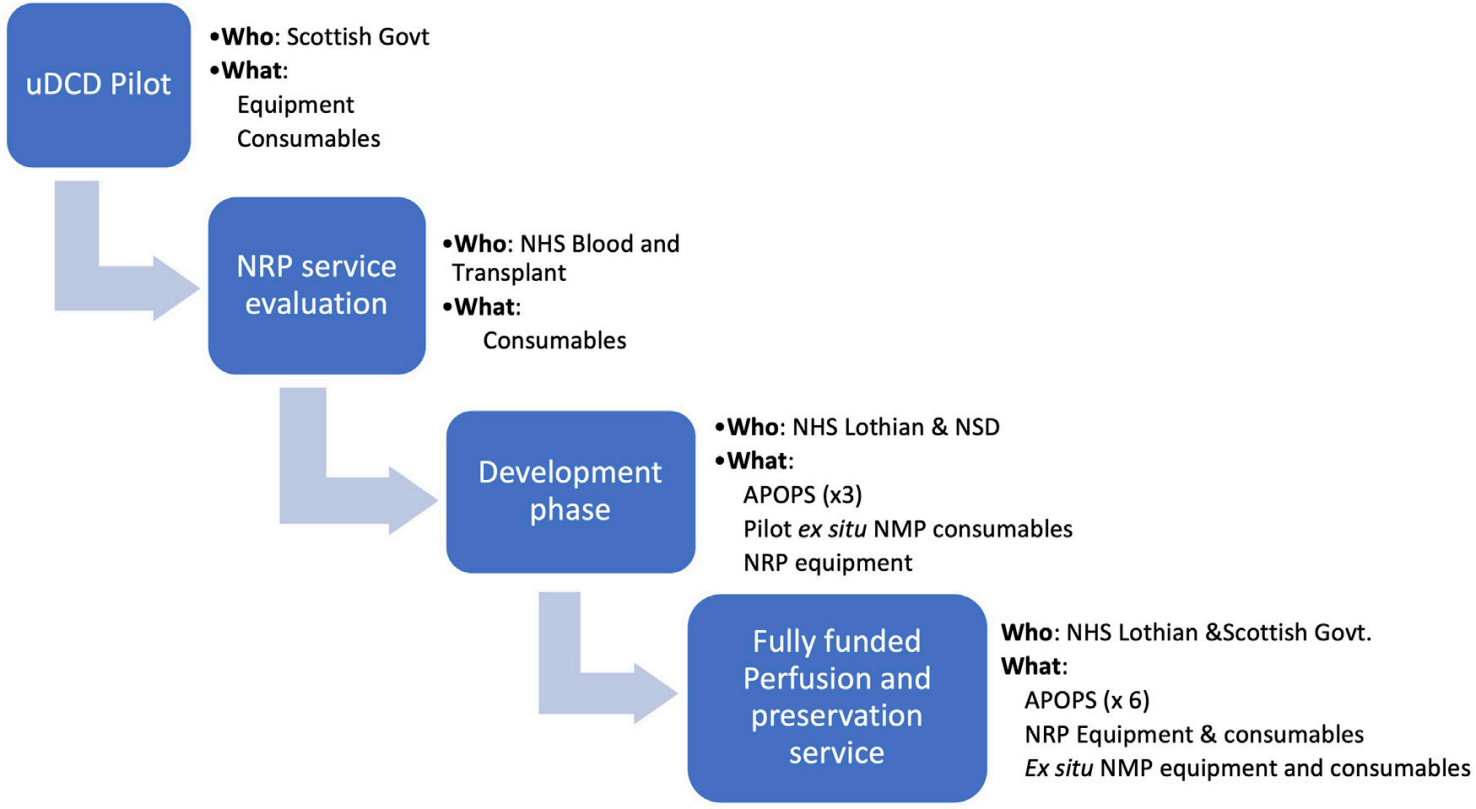
Funding strategy from pilot project to a fully funded service. APOPS, advanced perfusion and organ preservation specialists; NMP, normothermic machine perfusion.

The development of the NRP programme required financial support from several sources at the different stages of development (Figure 9). Initial setup (equipment, consumables) was funded as part of a clinical pilot for uncontrolled donation (20) during which the feasibility of donation from A&E was explored. This allowed the parallel development of the use of NRP in controlled DCD donation which generated the initial clinical data (21) that helped secure a more significant financial support from NHS Blood and Transplant as part of a wider Novel Technologies service evaluation. Although this funding covered the consumables for the procedures, it was insufficient to support the development of the team infrastructure and therefore additional (initially temporary) funding was secured from local funds, leading to the appointment of the first APOPS in 2016. The expanded programme demonstrated good clinical outcomes (4) that supported a successful funding application for additional APOPS from the local hospital to support the conceptual change in service delivery by a dedicated and self-sufficient team. At the same time, a cost-effectiveness study was undertaken based on clinical data generated by the UK programmes (26) which demonstrated that the incremental cost of using NRP to recover DCD livers (£4,500/procedure) is economically justified, leading to a cost saving of £1.17 million for every 100 NRP DCD cases. These data mirror the Spanish experience where cost utility is one of the drivers for the increased use of NRP. The new Donation and Transplantation Plan for Scotland: 2021 to 2026 (27), identified the use of novel technologies as a key strategy to increase organ utilisation and supported the roll-out of machine perfusion technology as a medium-term goal. The results from the NRP and the *ex situ* NMP feasibility work led to full funding of the Organ Perfusion and Preservation Service as of 1 April 2022.

## Discussion

DCD donation has seen a rapid expansion and in many countries provides a significant contribution to the donor pool (28). Whilst very good DCD renal organ utilisation and transplant outcomes have been achieved, the situation is different for other solid organs where lower utilisation rates, increased complication rates, and inferior long-term survival rates have been reported (29). Despite several refinements in the donation process and more stringent selection criteria, it is really the advent of novel *in situ* and *ex situ* perfusion technologies that holds the greatest promise for a leap improvement in utilisation and outcomes. While the place of individual technologies in the transplant process and the benefit each provides is yet to be fully defined, they all share the common challenge of translation from experimental to routine clinical procedure.

The novel perfusion and preservation strategies are inherently technologically complex and the magnitude of change to routine practice required has often been seen as a barrier to implementation and risk for non-adoption. NRP sits at the higher end of complexity as it requires not only supplementation of skills but also a significant change in logistics. Furthermore, despite excellent reported results (13, 15, 21), the technique has not been subjected to randomised controlled trials. When introduced in our centre, NRP was still in a developmental stage, requiring further refinement of the technical details and equipment. As such, the service evolved from selected donors attended by a small group of enthusiasts when available, through to a detailed evaluation of safety, clinical and non-clinical outcomes, protocols, standard operating procedures, and reporting mechanisms, leading to the current arrangement of NRP for all DCD donors attended by the team (regardless of donor history).

Failure to adopt new clinical techniques (even with unequivocally proven benefits) is unfortunately common (30), and therefore the implementation strategy requires just as much attention as the technique itself. This is not usually the result of technology failure but the resistance of the organisation to embrace change, which appears to be independent of size, academic status, resources, innovation history, or support from senior management (31). To minimise the risk of failure and non-adoption, we progressed through an iterative process, with several key elements such as recruitment, education, and training that had a transformative impact on service delivery and outcomes. This was supported by an awareness campaign to reach all stakeholders in the donation, retrieval, and transplant process.

Several key steps to the successful implementation of novel technology have been described (31) with the use of “full team dry-runs” as a fundamental step in the training process which enable the team to work together in standard as well as troubleshooting scenarios. The training process evolved from covering theoretical aspects and practical skills to including dry runs and full simulations that provided the opportunity for a technical run but also highlighted the necessity for interdependence and communication between all team members, fostering trust and confidence prior to actual donor attendance. This was supported by the development of relevant educational material, competency frameworks, and a training pack to support the training session which helped participants to implement similar programmes in their home institutions. This structured approach allowed us to support training teams that implemented the Dutch and Swedish NRP programmes.

Leadership is critical in driving change and enabling the successful introduction of any innovation. Whilst the input of senior clinicians and support from the organisation was critical in the initial stages when we demonstrated the safety and feasibility of NRP, it was the creation of a dedicated advanced perfusion and organ preservation specialist role that led to a significant change in the delivery of the service. This role focused exclusively on the delivery of perfusion technologies and took ownership of the NRP process and logistics, provided leadership and training, and facilitated the implementation of the changes in the pathways. The expansion of the NRP service enabled by these appointments was followed by the seamless implementation of the *ex situ* normothermic liver perfusion programme, with the two programmes contributing 38% of the current liver transplant activity in our centre.

Such developments could not have been possible without funding. Whilst the initial stages were supported through projects or local finances, the full development of the programme required a cost-effectiveness analysis. This was timely as it coincided with a new donation and transplantation strategy that identified the novel perfusion technology as a key priority. The clinical results of the NRP and the *ex situ* NMP programmes and the impact on organ utilisation and clinical activity lead to the establishment of a fully funded organ perfusion and preservation service.

At a systems scale, NRP and all *ex situ* perfusion technologies will operate in a complex and dynamic socio-technical environment with a sophisticated web of structural, socio-political interdependencies and continuous technological evolution (17). Therefore, it is important to define the framework for these technological developments. As the evidence for a beneficial impact gathers pace, the implementation will be dependent on the willingness to adopt and integrate these innovations into practice. The approach described herein demonstrates that this is achievable and provides adaptability and organisational resilience to continued change as illustrated by the successful implementation of two different perfusion technologies. This can only be made possible by support from a healthcare organisation with the capacity to innovate and fund these disruptive changes into routine clinical practice as was the case for us (17).

In summary, NRP is a highly-disruptive technology with a transformative impact on DCD transplantation. Herein we advocate a stepwise approach to training and service delivery, coupled with a well-defined framework, face-to-face leadership, and teamwork that has proven to be very effective in our centre. Whilst some details may require local adaptation, the blueprint described should accelerate wider and sustainable adoption of all organ perfusion and preservation technologies in the future.

## Data Availability

De-identified data for the purpose of meta-analyses, statistical analysis plans and data dictionary are available on request which should be sent to the corresponding author (gabriel.oniscu@ed.ac.uk). Data requestors will need to sign a data access agreement.

## References

[1] De Beule J Vandendriessche K Pengel LHM Bellini MI Dark JH Hessheimer AJ A Systematic Review and Meta‐analyses of Regional Perfusion in Donation after Circulatory Death Solid Organ Transplantation. Transpl Int (2021) 34(11):2046–60. 10.1111/tri.14121 34570380

[2] NHSBT. NHSBT Annual Report on the National Organ Retrieval Service (2020-21) (2021). Available from: https://nhsbtdbe.blob.core.windows.net/umbraco-assets-corp/24643/annual-report-on-the-national-organ-retrieval-service-202021.pdf (Accessed May 16, 2022).

[3] NHSBT. NHSBT Annual Report on Liver Transplantation (2020-21) (2021). Available from: https://nhsbtdbe.blob.core.windows.net/umbraco-assets-corp/19867/nhsbt-liver-transplant-report-1920.pdf (Accessed May 16, 2022).

[4] Watson CJE Hunt F Messer S Currie I Large S Sutherland A *In Situ* normothermic Perfusion of Livers in Controlled Circulatory Death Donation May Prevent Ischemic Cholangiopathy and Improve Graft Survival. Am J Transpl (2019) 19(6):1745–58. 10.1111/ajt.15241 30589499

[5] Mittal S Gilbert J Friend PJ. Donors after Circulatory Death Pancreas Transplantation. Curr Opin Organ Transpl (2017) 22(4):372–6. 10.1097/mot.0000000000000437 28678058

[6] Richards JA Roberts JL Fedotovs A Paul S Cottee S Defries G Outcomes for Circulatory Death and Brainstem Death Pancreas Transplantation with or without Use of Normothermic Regional Perfusion. Br J Surg (2021) 108(12):1406–8. 10.1093/bjs/znab212 34155506 PMC10364865

[7] Goussous N Alvarez-Casas J Dawany N Xie W Malik S Gray SH Ischemic Cholangiopathy Postdonation after Circulatory Death Liver Transplantation: Donor Hepatectomy Time Matters. Transpl Direct (2022) 8(1):e1277. 10.1097/TXD.0000000000001277 PMC8710320 34966844

[8] Karangwa S Panayotova G Dutkowski P Porte RJ Guarrera JV Schlegel A. Hypothermic Machine Perfusion in Liver Transplantation. Int J Surg (2020) 82:44–51. 10.1016/j.ijsu.2020.04.057 32353556

[9] De Deken J Kocabayoglu P Moers C. Hypothermic Machine Perfusion in Kidney Transplantation. Curr Opin Organ Transpl (2016) 21(3):294–300. 10.1097/mot.0000000000000306 26945319

[10] van Rijn R Schurink IJ de Vries Y van den Berg AP Cortes Cerisuelo M Darwish Murad S Hypothermic Machine Perfusion in Liver Transplantation - A Randomized Trial. N Engl J Med (2021) 384:1391. 10.1056/nejmoa2031532 33626248

[11] Jochmans I Brat A Davies L Hofker HS van de Leemkolk FEM Leuvenink HGD Oxygenated versus Standard Cold Perfusion Preservation in Kidney Transplantation (COMPARE): A Randomised, Double-Blind, Paired, Phase 3 Trial. Lancet (2020) 396(10263):1653–62. 10.1016/s0140-6736(20)32411-9 33220737

[12] Nasralla D Coussios CC Coussios CC Mergental H Akhtar MZ Butler AJ A Randomized Trial of Normothermic Preservation in Liver Transplantation. Nature (2018) 557(7703):50–6. 10.1038/s41586-018-0047-9 29670285

[13] Hessheimer AJ Coll E Torres F Ruíz P Gastaca M Rivas JI Normothermic Regional Perfusion vs. Super-rapid Recovery in Controlled Donation after Circulatory Death Liver Transplantation. J Hepatol (2019) 70(4):658–65. 10.1016/j.jhep.2018.12.013 30582980

[14] O'Neill S Srinivasa S Callaghan CJ Watson CJ Dark JH Fisher AJ Novel Organ Perfusion and Preservation Strategies in Transplantation - Where are We Going in the UK? Transplantation (2020) 104:1813. 10.1097/TP.0000000000003106 31972706

[15] Antoine C Savoye E Gaudez F Cheisson G Badet L Videcoq M Kidney Transplant from Uncontrolled Donation after Circulatory Death: Contribution of Normothermic Regional Perfusion. Transplantation (2020) 104(1):130–6. 10.1097/tp.0000000000002753 30985577

[16] McCulloch P Altman DG Campbell WB Flum DR Glasziou P Marshall JC No Surgical Innovation without Evaluation: The IDEAL Recommendations. Lancet (2009) 374(9695):1105–12. 10.1016/s0140-6736(09)61116-8 19782876

[17] Greenhalgh T Wherton J Papoutsi C Lynch J Hughes G A'Court C Beyond Adoption: A New Framework for Theorizing and Evaluating Nonadoption, Abandonment, and Challenges to the Scale-Up, Spread, and Sustainability of Health and Care Technologies. J Med Internet Res (2017) 19(11):e367. 10.2196/jmir.8775 29092808 PMC5688245

[18] Fondevila C Hessheimer AJ Ruiz A Calatayud D Ferrer J Charco R Liver Transplant Using Donors after Unexpected Cardiac Death: Novel Preservation Protocol and Acceptance Criteria. Am J Transpl (2007) 7(7):1849–55. 10.1111/j.1600-6143.2007.01846.x 17564639

[19] Thuong M Ruiz A Evrard P Kuiper M Boffa C Akhtar MZ New Classification of Donation after Circulatory Death Donors Definitions and Terminology. Transpl Int (2016) 29(7):749–59. 10.1111/tri.12776 26991858

[20] Reed MJ Currie I Forsythe J Young I Stirling J Logan L Lessons from a Pilot for Uncontrolled Donation after Circulatory Death in the ED in the UK. Emerg Med J (2020) 37(3):155–61. 10.1136/emermed-2019-208650 31757833

[21] Oniscu GC Randle LV Muiesan P Butler AJ Currie IS Perera MTPR In SituNormothermic Regional Perfusion for Controlled Donation after Circulatory Death-The United Kingdom Experience. Am J Transpl (2014) 14(12):2846–54. 10.1111/ajt.12927 25283987

[22] Neuberger J. Organisational Structure of Liver Transplantation in the UK. Langenbecks Arch Surg (2015) 400(5):559–66. 10.1007/s00423-015-1296-9 25761844

[23] Manara A Shemie SD Large S Healey A Baker A Badiwala M Maintaining the Permanence Principle for Death during *In Situ* Normothermic Regional Perfusion for Donation after Circulatory Death Organ Recovery: A United Kingdom and Canadian Proposal. Am J Transpl (2020) 20(8):2017–25. 10.1111/ajt.15775 PMC7540256 31922653

[24] Parent B Moazami N Wall S Carillo J Kon Z Smith D Ethical and Logistical Concerns for Establishing NRP‐cDCD Heart Transplantation in the United States. Am J Transpl (2020) 20(6):1508–12. 10.1111/ajt.15772 31913567

[25] Lee E Johnston CJC Oniscu GC. The Trials and Tribulations of Liver Allocation. Transpl Int (2020) 33(11):1343–52. 10.1111/tri.13710 32722866

[26] Healthcare Improvement Scotland. Scottish Health Technologied Group. Organ Retrieval Using *In Situ* Normothermic Regional Perfusion (NRP) for Liver Transplantation. Evidence Synthesis nr 2 (2019). Available from: https://shtg.scot/our-advice/organ-retrieval-using-in-situ-normothermic-regional-perfusion-nrp-for-liver-transplantation (Accessed May 1, 2022).

[27] Scottish Government. Donation and Transplantation Plan for Scotland: 2021-2026 (2022). Available from: https://www.gov.scot/publications/donation-transplantation-plan-scotland-2021-2026/documents/ (Accessed May 1, 2022).

[28] Lomero M Gardiner D Coll E Haase‐Kromwijk B Procaccio F Immer F Donation after Circulatory Death Today: An Updated Overview of the European Landscape. Transpl Int (2020) 33(1):76–88. 10.1111/tri.13506 31482628

[29] Rijkse E Ceuppens S Qi H IJzermans JNM Hesselink DA Minnee RC. Implementation of Donation after Circulatory Death Kidney Transplantation Can Safely Enlarge the Donor Pool: A Systematic Review and Meta-Analysis. Int J Surg (2021) 92:106021. 10.1016/j.ijsu.2021.106021 34256169

[30] Kurian D Gorcos J Meinke S Thirumavalavan N Mizrahi I Kiani S Change Management and an Innovative Approach to Heart Bypass Surgery. Phys Exec (2011) 37(6):30–7. PMC4146430 22195414

[31] Edmonson AC Bohmer RM Pisano GP. Disrupted Routines: Team Learning and New Technology Implementation in Hospitals. Adm Sci Q (2001) 46(4):685–716. 10.2307/3094828

