# Supplementary material for: From Haphazard to a Sustainable Normothermic Regional Perfusion Service: A Blueprint for the Introduction of Novel Perfusion Technologies
Source: Transpl Int. 2022 Jun 3;35:10493. doi: 10.3389/ti.2022.10493 (PMC9203686; doi:10.3389/ti.2022.10493)

Normothermic Regional Perfusion

Training Pack

Edinburgh Transplant Centre NRP Team

Edinburgh Transplant Centre ©

October 2021. Review October 2022.

UK Protocol for Normothermic Regional Perfusion (NRP) in controlled Donation after Circulatory determination of Death

NRP NATIONAL PROTOCOL

Version number: 1.5
Date: 7th February 2022

Responsible author: Chris Watson, co-chair of the Novel Technologies Implementation Group

1 Preface

This protocol was produced by the NHSBT NRP implementation group (see section 11). The protocol reflects the combined experience of UK experts over many years developing NRP in the UK. It is acknowledged that it reflects practices that have evolved locally and which may be superseded as more evidence and experience accrues. It also reflects the equipment available to clinicians at the time of writing and is not meant to endorse any particular piece of equipment.

2 Table of contents

1 Preface 2
2 Table of contents 2
3 Amendments....4
3.1 Version 1.0 Dated 18th March 2021....4
3.2 Version 1.1. Dated 19th March 2021....4
3.3 Version 1.2, dated 8th April 2021. 4
3.4 Version 1.3, dated 10th April 2021 4
3.5 Version 1.4, dated 25th May 2021....4
3.6 Version 1.4.2, dated 29th June 2021....4
3.7 Version 1.5, dated 7th February 2022....5
4 Introduction 5
5 Composition of Organ Retrieval Team for A-NRP....5
6 Mobilisation 6
7 Setting up the equipment for A-NRP 6
7.1 Ordering blood....6
7.2 Donor hospital handover 6
7.3 Timing and Place of Withdrawal of Life Supportive Therapy (WOLST) 6
7.4 Pre-retrieval preparation 7
7.5 NRP setup....8
7.5.1 The heater....8
7.5.2 Heparin in the donor before circulatory arrest 8
7.5.3 Preparation of cold perfusion fluids 8
7.6 Composition of circuit priming fluid 9
7.6.1 Standard prime 9
7.6.2 Anaemic donor (Hb<70gm/L) 9
7.6.3 Small donor or paediatric donor....9
7.6.4 Additional fluids during retrieval 9
7.6.5 Additional heparin during retrieval 9
8 Surgical Protocol for DCD NRP....10

8.1 Cannulation 10
8.1.1 Femoral cannulation 10
8.1.2 Aorto-iliac cannulation 11
8.1.3 The contralateral external iliac artery 11
8.2 Controlling the thoracic aorta 11
8.3 Maastricht 4 controlled DCD donors 12
8.4 Establishing NRP 12
8.5 Haemodynamic and biochemical goals 13
8.6 During NRP 13
8.6.1 Haemostasis 13
8.6.2 Direct retrieval and perfusion of lung or heart 13
8.6.3 Additives to the perfusion fluid 13
8.7 Surgical dissection 14
8.8 Cold perfusion 14
8.9 Biochemical Evaluation 15
8.9.1 Liver 15
8.9.2 Pancreas 15
8.9.3 Kidneys 15
8.10 Post NRP 15
8.11 Failure to establish NRP 15
9 Documentation 15
10 Troubleshooting NRP 16
10.1 Communication 16
10.2 Volume loss 16
10.3 Air in the circuit 16
10.4 Clots in the circuit 17
10.5 Troubleshooting Cs for poor flows 17
11 Appendix 1 Protocol for direct recovery and perfusion of the heart and A-NRP 18
11.1 Cardiac team requirements for successful cardiac recovery 18
11.2 Abdominal team requirement for successful cardiac recovery during A-NRP 18
11.3 SNOD requirements 18
11.4 Circuit 18
11.4.1 Preparation 18
11.4.2 Prime solution 18
11.5 Operative procedure 20
11.5.1 Abdominal team procedure 20
11.5.2 Cardiac procedure 21
11.5.3 Procedure for lung retrieval alone without heart while on A-NRP 21

12 Appendix: UK NRP implementation group members involved in drafting the protocol.22

3 Amendments

3.1 Version 1.0 Dated 18th March 2021

Final protocol agreed 18/3/21.

3.2 Version 1.1. Dated 19th March 2021

Removal of reference to previous antibiotic protocol and correction of typographical errors.

3.3 Version 1.2, dated 8th April 2021.

Introduction of clamping the SVC and IVC in the chest to prevent cardiac filling and reanimation.

3.4 Version 1.3, dated 10th April 2021

Additional note to make it clear that venting the ascending aorta is mandatory in all cases of A-NRP (including Maastricht 4) where no cardiothoracic team is attending.

3.5 Version 1.4, dated 26th June 2021

Change in requirement when an Endoclamp is used to require formal venting of the ascending aorta with a separate cannula.

Removal of requirement to clamp the IVC and SVC (amendment 3.3)

Revert to adding antimicrobials to the prime.

Recommendation to cannulate artery first.

Recommendation for someone to call out times at 10, 15, 20 minutes following knife to skin to guide surgeon on when to abandon and go cold.

Guidance on amount of red cells to be added to prime if donor is anaemic.

Suggestion to ligate the artery supplying the contralateral leg if femoral or iliac artery cannulation has taken place.

Alteration in requirement for liver function tests from every 30 minutes to every 30-60 minutes.

Further notes on Maastricht category 4 donors:

- Addition of 5 minute stand-off in 4 donors
- Possibility of delivery of UW down aortic root if heart fibrillates.
- No need to ligate neck vessels for TA-NRP

3.6 Version 1.4.2, dated 29th June

Addition of section 9 regarding documentation of NRP on organ passport.

3.7 Version 1.5, dated 7th February 2022

Removal or requirement for cultures during NRP

4 Introduction

Abdominal in situ normothermic regional perfusion (A-NRP) is a technique to restore the circulation to the abdominal organs following circulatory arrest for the purpose of transplantation. This involves establishing a localised, abdominal perfusion Extracorporeal Membrane Oxygenation (ECMO) circuit, perfusing the organs with oxygenated blood at 37°C for a period of typically 2 hours.

This will allow:

1. Recovery from warm ischaemia and replenishment of ATP reserves
2. Assessment of organ function and quality
3. A less hasty retrieval.

NRP has been shown to increase the utilisation of all abdominal organs, and significantly improve the outcomes of liver and kidneys, with no adverse effects on the pancreas. For the liver it is associated with better transplant survival and a very low incidence of cholangiopathy when compared to conventional DCD donor livers; for the kidney it is associated with better renal function at 12 months.

This protocol details the technical aspects of the procedure. It is written with regard to the current legislation and observes the Academy of Royal Medical Colleges (AoRMC) code of practice for the diagnosis and confirmation of death that forms the basis of organ donation from deceased donors.

5 Composition of Organ Retrieval Team for A-NRP

With the addition of NRP to clinical practice, the core membership of the abdominal retrieval team should consist of:

- A theatre practitioner scrubbing for organ retrieval
- Organ preservation practitioner: A theatre practitioner competent in organ perfusion/preservation techniques:
- Advanced Perfusion and Organ Preservation Specialist (APOPS): A senior theatre practitioner competent in operating the NRP machine and monitoring the perfusion
- Two surgeons, at least one of whom has been accredited as competent to perform A-NRP

Thoracic and/or cardiac surgeons may attend for retrieval of thoracic organs; separate protocols exist for recovery of those organs and adaptations of this protocol in those circumstances are discussed at the end of this document.

6 Mobilisation

Mobilisation for A-NRP is the same as for any DCD retrieval, arriving 1 to 2 hours or more before the proposed withdrawal time. For combined procedures with cardiothoracic teams the scheduled arrival should be 2 hours ahead of proposed withdrawal time to enable full communication and rehearsal of the steps involved in retrieval to ensure a successful outcome for both teams. It should be noted that the abdominal team will need at least 2 additional vascular clamps or equivalent when a combined retrieval is planned with the cardiothoracic team. These clamps, applied to large vessels to permit heart, and/or lung retrieval, will remain in place one the cardiac team have gone and must therefore be carried by the abdominal NRP team.

7 Setting up the equipment for A-NRP

7.1 Ordering blood

The SNOD should order packed red cells cross-matched to the organ donor so they are available prior to the abdominal retrieval team arriving. The blood should be in the theatre suite *before* withdrawal of treatment. The amount ordered depends on circumstances:

- Standard abdominal NRP: 4 units
- Abdominal NRP with heart or lungs are being recovered by direct retrieval and cold perfusion: 8 units
- Thoraco-abdominal NRP: 6 units

7.2 Donor hospital handover

Once at the donor hospital the abdominal retrieval team should inform local theatre staff of the planned procedure. They should also discuss the relevant protocols for removal of heart or lungs with the respective retrieval team. Protocols are clearly defined for combined procedures and must be followed (see odt.nhs.uk microsite).

Someone should be identified who can perform blood gas analyses if a point of care device is not available in the donor theatre. Similarly, someone should be identified to run samples to the biochemistry laboratory if a point of care device is not available. However, each team should have the necessary equipment, fully maintained and quality assured, and be independent of laboratories for such assays.

The standard pre-retrieval handover should be undertaken, including verification of information in the core donor data form by reviewing the case notes, and checking the blood group and virology results.

7.3 Timing and Place of Withdrawal of Life Supportive Therapy (WOLST)

The normal practice at the donor hospital regarding the place of treatment withdrawal may be observed. The time of withdrawal, the time systolic BP falls under 50mmHg, and the time of circulatory arrest are recorded according to current practice. In addition, the time NRP is commenced and stopped will also need to be recorded.

As soon as death is verified, the patient is transferred to the operating theatre.

The acceptable duration between WOLST and death for retrieval of specific organs should be discussed with the implanting team, but these are likely to be longer than considered with DCD donors in whom NRP is not performed. As per NORS guidance the retrieval team is expected to stay for 3 hours. There should be no need for premature stand down since viability will be tested during NRP.

7.4 Pre-retrieval preparation

This involves several steps:

a) The scrub practitioner will setup the operating environment in a similar fashion to the current practice for DBD retrieval. This includes setting up the diathermy machine (as available in the host theatre) and the power saw for thoracotomy (where available).
b) Two 60 ml catheter tip syringes filled with heparinised saline should be prepared. They will be used to flush the aortic and IVC lines prior to connecting to the NRP circuit.
c) The aortic and IVC cannulas should be identified.
d) The connections between the proposed cannulae and the sash are checked to make sure they are compatible and no additional connectors are required.
e) Once the potential donor’s systolic pressure has fallen below 50mHg or after circulatory arrest, the sterile part of the NRP circuit (the “sash”) should be handed to the scrub practitioner.
f) Both limbs of the sash should be clamped approximately 10cm from the ends of the red and blue line tubes and divided.
g) The arterial and venous cannulae should be opened at this stage; opening them earlier may result in wastage if the donor does not die.
h) If a heart team is planning to recover the heart by direct recovery and perfusion in the cold, they will need 1.5L of donor blood to prime the OCS heart machine. This must be recovered before NRP commences. This is best facilitated by having a Y-connector on the blue venous return pipe above the reservoir that can be connected to their blood receptacle. This is best placed before priming if one is not already built into the circuit (figure 1).

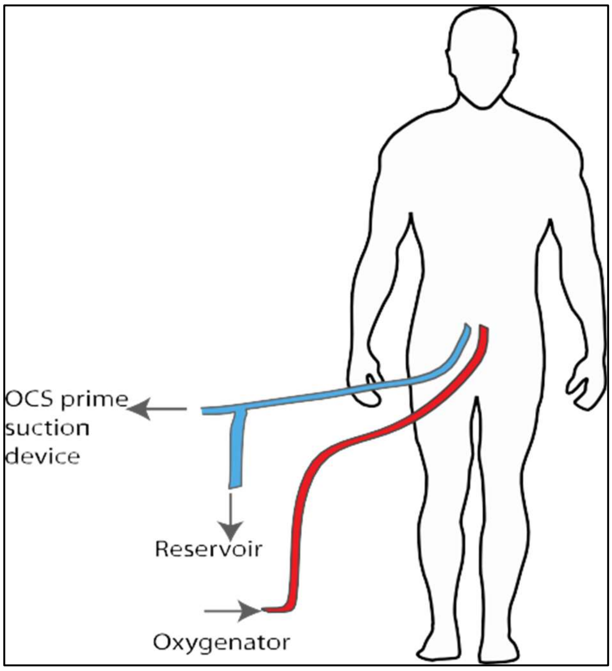

Figure 1 Y-connector to facilitate blood being removed to prime the OCS device.

7.5 NRP setup

The NRP setup depends on the machine used.

7.5.1 The heater

Maquet's Cardiohelp: The heater is separate and should be topped up with water and switched on with the temperature set at 37°C.

Organ Assist's Donor Assist: switching on the heater is part of the automated setup.

7.5.2 Heparin in the donor before circulatory arrest

Maastricht 3 donors: No pre-mortem interventions are currently allowed in the UK.

Maastricht 4 donors (donor is already certified dead by brain stem criteria): heparin can be given. A suggested dose is 300units/kg (around 25000 units for a 80kg person) given just prior to withdrawal of treatment

7.5.3 Preparation of cold perfusion fluids

Two one-litre bags of cold University of Wisconsin solution should be prepared with 300u/kg (around 25000 units for an 80kg man) of heparin added to *each*, as for a standard DCD, and run through a large bore 'Y' giving set so they can be used immediately should NRP fail to be established or problems are encountered during perfusion and rapid conversion to a standard technique is required. The giving set may be pre-connected to the NRP circuit and once primed the UW bags should be replaced in ice until needed. When cold perfusion starts it is

imperative a clamp is placed on the arterial line proximally to prevent back flow into the pump/reservoir.

7.6 Composition of circuit priming fluid

7.6.1 Standard prime

- Bicarbonate 8.4%, 1ml/kg
- Hartmann's 2000 mls
- Heparin 50,000 u
- Methylprednisolone: 1 gram
- Phentolamine 5mg
- Pancuronium 12 mg – to prevent abrupt diaphragmatic contraction when phrenic nerve is divided which can cause distress to attending teams and host staff.
- Fluconazole: 400 mg
- Antibiotics to be added into the prime:
    - 200mg teicoplanin
    - 120mg gentamicin
    - 500mg metronidazole

7.6.2 Anaemic donor (Hb<70gm/L)

If the donor is anaemic a unit or more of packed red cells may be added to the reservoir in place of some of the Hartmann's solution. Typically if less then 6gm/L, add 2 units packed red cells; if between 6 and 8gm/L, add 1 unit packed red cells.

7.6.3 Small donor or paediatric donor

If the donor is small the dilution effect of the prime solution will be large. Therefore:

- *For donors >30kg but <50kg,* add 2 units of blood to the prime and only 1000ml of Hartmann's.
- *For donors ≤30kg*, use 3 units of blood in the prime with 500mls Hartmann's.

Flow rates will be proportionately lower in smaller donors.

7.6.4 Additional fluids during retrieval

During perfusion it is usually necessary to add more volume, in which case Gelofusine or blood are appropriate. *DO NOT ADD Hartmann's* once perfusion has started as it contains lactate and makes the lactate result impossible to interpret.

7.6.5 Additional heparin during retrieval

During perfusion an ACT *may* be checked or, in the absence of this, additional heparin *may* be given at 90 min using a dose of 150 u/kg.

Heparin should also be added if severe haemorrhage occurs, e.g. during cardiac retrieval, and is replaced by a lot of bank blood, since this will dilute out any existing heparin with the risk of clotting. This is not usually required in A-NRP only.

8 Surgical Protocol for DCD NRP

Before withdrawal of treatment the operating surgeon and pump operator should check how the chosen cannulae are connected to the circuit, and whether any additional connectors are necessary (see above).

8.1 Cannulation

Cannulation can either be in the groin using the femoral vessels, or in the abdomen with direct or indirect access to the aorta and IVC. Surgeons need to be familiar with all techniques. It is helpful to have the perfusion practitioner call out the time taken at 10, 15, and 20 minutes following knife to skin to inform the surgeon. Cannulation should not take longer than 20 minutes, and will typically take around 10 to 15 minutes.

8.1.1 Femoral cannulation

This is a simple and rapid technique, and is most appropriate in the following circumstances:

- for younger donors with little chance of occlusive ilio-femoral arterial disease
- when thoracic surgeons wish rapid access to the chest
- where access to the abdominal vessels may be delayed (e.g. previous surgery; ankylosing spondylitis where the patient may not be flat on the operating table)
- for known vascular anomalies seen on available cross-sectional imaging (e.g. known lower polar vessels coming off distal abdominal aorta/iliacs; retro-aortic left renal vein originating at confluence of iliac veins; left sided IVC, horseshoe or pelvic kidney).

A transverse or oblique incision is made immediately below the inguinal ligament. When the heart is being retrieved, the vein must be cannulated first to allow rapid blood drainage to prime the OCS machine; otherwise it may be easier to cannulate the artery first.

The femoral vein is isolated, the distal end either clamped or ligated. A venous cannula is passed up the femoral vein and secured either with a ligature or snugger. The cannula is then connected to the venous limb of the sash, and the sash's venous clamp removed to allow drainage into the reservoir.

The femoral artery is isolated, distal end ligated, and cannulated. The cannula is secured with a ligature or snugger, and connected to the arterial limb of the sash with care taken to exclude air.

The choice of cannulae for the femoral vessels varies according to the patient's size:

- *Femoral artery:* typically a 19F French cannula (e.g. Medtronic Biomedicus 19F), although alternative 15Fr, 16Fr or 18Fr cannulas may be required in small patients or for diseased arteries.
- *Femoral vein*: a 25 French (e.g. Medtronic Biomedicus 25F/38cm) cannula

It is possible that difficulties arise during femoral vein or arterial cannulation and the surgeon needs to be prepared to switch to abdominal cannulation in such eventualities. Typically the extra time taken is compensated by being able to restore a circulation to the abdominal organs before cold storage.

8.1.2 Aorto-iliac cannulation

A midline incision from xiphoid to pubis is made. The right colon and small bowel mesentery are mobilised and retracted to the left by the assistant. The aorta or right common iliac artery may be cannulated first in preference to the IVC, since any venous bleeding will make subsequent identification of the artery difficult.

The distal infrarenal aorta or right common iliac artery is identified and slung using a vascular snugger. The distal aorta is cross clamped or ligated. The aortic cannula is inserted, checking the proximal position of the tip is approximately 2 to 5cm above the cannulation point. The cannula is secured in place with the vascular snugger or ligature. The arterial limb of the circuit (the tube with the red line) is then connected to the cannula, with care taken to eliminate all air bubbles (alternatively, this can be pre-connected and the perfusion practitioner can forward flush slowly during cannulation).

The infrarenal IVC (or right common iliac vein) is dissected and encircled using a ligature or vascular snugger. The distal end is clamped or ligated. The venous cannula is inserted into the IVC. The cannula should be adjusted so its tip sits just below the diaphragm to allow the clamping of the suprahepatic IVC without compromising the venous return in the circuit. The venous limb of the circuit (the tube with the blue line) is then connected to the cannula and the clamp released. Blood should flow back into the reservoir.

The choice of cannulae for the intra-abdominal vessels varies less with the patient's size than when cannulating in the groin. The same cannulas as used for a femoral approach may be used, or alternative larger cannulas may be used:

- *Aorta or common iliac artery:* a 20 or 24Fr cannula (e.g. DLP Medtronic cannula).
- *IVC or common iliac vein*: a multistage cannula of 25 French or more (e.g. 36/46 Fr 40 cm Edwards Lifesciences Q3 Trim-Flex two or three Stage).

8.1.3 The contralateral external iliac artery

If the cannulation has been into a femoral artery or common iliac artery, the contralateral common external iliac artery may be ligated or clamped to optimise abdominal organ perfusion. Care should be taken not to reduce the length available for subsequent vascular reconstructions.

8.2 Controlling the thoracic aorta

A rapid sternotomy is carried out using either a power saw or Gigli saw. If cannulation was in the groin the abdomen does not need to be opened first. The thoracic aorta is clamped below the level of the left subclavian artery close to the diaphragm. A stab incision is made in the ascending thoracic aorta with a number 11 blade and a 24G cannula (the hole left by an 11

blade inserted to its hilt is the same size as the 24G cannula) or a Medtronic DLP Aortic Root Cannula (avoiding the need for a prior stab) is inserted and left open to atmosphere to allow monitoring of pressure and flow in the aorta and intracranial arterial supply. Typically, there is a small column of dark deoxygenated blood 1 to 5cm. This aortic vent should have no flow within it; if there is flow the pump should be stopped and the thoracic aortic clamp repositioned. The aortic vent can be connected to the reservoir if the column of blood reaches the top of the cannula, so long as it does not represent oxygenated blood from the NRP circuit.

An alternative approach is to insert an aortic endo-clamp (e.g. Cook 32/37mm, 120cm Coda LP balloon catheter) in the descending thoracic aorta. It is critical to check the length prior to insertion to ensure that the balloon is positioned in the descending aorta, and not in the left subclavian or anomalous right subclavian artery. An ascending aortic vent cannula still needs to be inserted, after which the NRP circuit can be started. This approach would allow the cardiothoracic team to undertake the sternotomy and mobilise the lung and clamp the descending aorta (if simultaneous lung retrieval).

NOTE: The ascending thoracic aorta **MUST** always be vented before starting NRP including in Maastricht 4 donors, and the vent kept open for the duration of NRP.

8.3 Maastricht 4 controlled DCD donors

Maastricht 4 donors, which are brain stem dead donors going through the DCD pathway usually at the request of the next of kin, pose a number of issues worth noting.

i. The donor can be heparinised before ventilation is discontinued
ii. The National Organ Donation Committee have agreed that a no touch period of 5 minutes following circulatory arrest be respected before the donor is transferred to the operating room and surgery commenced.
iii. An aortic vent should always be placed in a category 4 donor
iv. Since the next of kin have concerns about the beating heart, the donor team should consider infusing UW solution (or cardioplegia) into the aortic root via the vent cannula, with a temporary clamp distal to this, to cause a cardioplegic arrest and suppress fibrillation if this occurs.
v. In cases of thoraco-abdominal NRP in category 4 donors, there is no need to ligate or separately vent the neck vessels.

8.4 Establishing NRP

- The pump must only be started once the circuit is completely connected, the thoracic aorta is cross-clamped/occluded and the aorta above the clamp is vented.
- The heater temperature should be 37°C.
- The air/O_2 mixer should be set to deliver gas flow at 2 litres/minute with a starting FiO_2 of 21% (air). High FiO_2 should be avoided. Changes to the oxygen/air mixture may be required subsequently depending on the blood gas analysis. High oxygen concentrations may generate reactive oxygen species and can exacerbate reperfusion injury to the organs.

- The preferred NRP duration is two hours.

8.5 Haemodynamic and biochemical goals

The following parameters are suggested:

- Pump flow 2-3 litres/minute
- Temperature 35.5°C - 37.5°C
- Air / O_2 to maintain a venous O_2 saturation (SvO_2) 60-80%
- Arterial pH 7.35-7.45
- Haematocrit > 20%
- Gas flow to maintain arterial pCO_2 4.5 to 6.0 kPa.

8.6 During NRP

8.6.1 Haemostasis

Once the NRP is established, meticulous haemostasis must be ensured from the abdominal wound edges, sternotomy and retroperitoneal tissues disrupted during aortic and IVC cannulation. Bleeding is not usually troublesome for the first 60 minutes or so.

As there is a potential for significant blood loss, volume replacement (blood or colloid) should be readily available. If volume is lost from the circuit, the two most common sites are in the chest where the thoracic aorta is clamped, and where an intercostal or vertebral branch may have been avulsed, and around the aortic and caval cannulae. Volume loss is also observed routinely with vasodilation consequent on loss of sympathetic tone, without overt blood loss, and volume replacement will be required during the NRP procedure.

8.6.2 Direct retrieval and perfusion of lung or heart

There is potential for significant bleeding when lungs / or heart are retrieved and therefore there is a separate protocol for combined A-NRP with cold thoracic retrieval (see odt.nhs.uk microsite). The supra-hepatic IVC is clamped at the cavo-atrial junction in the chest and therefore it is important to ensure that the tip of the venous cannula is below the level of the diaphragm to avoid compromising the venous return. The thoracic surgeon must ligate the azygous vein, the SVC, and leave a clamp across the descending thoracic aorta.

8.6.3 Additives to the perfusion fluid

- Heparin may be added every 90 minutes (150 u/kg). More frequent heparin boluses will be required if a large quantity of bank blood (or cell saved blood) are added as may happen in cardiac retrievals.
- Bicarbonate should be added according to the initial blood gas results: if the pH<7.0 after starting NRP give 25ml of 8.4% immediately. Correction is seldom necessary once

NRP is established, since functioning liver and kidneys correct the acidosis quickly, and gas flow across the oxygenator can also be used to regulate pH.

- Subsequent volume replacement should either be red cells or gelofusine

8.7 Surgical dissection

Once NRP is established, the surgeon should perform a full laparotomy and a macroscopic evaluation of the abdominal organs, in particular the liver, pancreas and kidneys. At the start of NRP the liver will appear congested and feel stiff. As time passes the liver should feel less firm and a normal colour return. The bile duct should be divided early and the gallbladder incised, emptied and flushed with normal saline (0.9%), care being taken not to flush thick gallbladder bile into the common duct. A careful examination of small bowel, the blood supply to the cut end of the bile duct and the appearance of the gallbladder mucosa should be undertaken (indicative of ischaemia). If the lungs are not being retrieved the contents of the thoracic cavity should be inspected thoroughly looking for lung and oesophageal neoplasms and other pathology.

After these initial procedures it is often sensible to scrub out for 60 minutes to avoid unnecessary blood loss, leaving one person scrubbed in case of emergencies. In addition, dissection around the liver causes haemodynamic instability at a time when you are trying to allow the organs to recover from warm ischaemia.

In the face of excessive blood loss, it is preferable to stop NRP early, rather than to persist with transfusions for the full 2 hours.

8.8 Cold perfusion

The cold phase dissection is carried out as for DBD retrieval. The abdomen may be filled with ice slush just before cold perfusion commences. The cold inflow may be attached to a suitable port or Y-connector on the arterial side of the circuit distal to the oxygenator if not already attached – do not run the cold perfusate through the oxygenator as this will warm it up. Alternatively, the arterial line can be clamped and the cold UW giving set connected to tubing just proximal to the arterial cannula. The standard quantity of UW is infused into the aorta as for any normal retrieval.

The cannulation of the portal vein in the cold phase is at the discretion of the retrieving surgeon. The portal vein ***must*** be extensively flushed with UW on the back table if it is not cannulated and perfused *in situ*.

The venous cannula may be pulled back to aid drainage of the kidneys, as the cava will collapse on the cannula potentially impairing venous drainage. The venous effluent may be collected into the circuit's reservoir or separate drainage bag (depending on the NRP machine and circuit used), or it may be allowed to collect in the chest from where it can be sucked out.

8.9 Biochemical Evaluation

Serial samples (gases and ALT/AST every 30 to 60 minutes) are taken to assess the organs and to stay within the parameters described above; more frequent testing is appropriate where shortened periods of perfusion may occur, such as when cardiothoracic teams are involved. Volume replacement to support flows, whether blood or colloid, will dilute the biochemical markers of damage and function; this should be borne in mind when interpreting them.

Biochemical assessment should be done on machines that are quality assured (Human Tissue Authority requirement). This may be by the retrieval centre's own biochemistry department.

8.9.1 Liver

The following biochemical parameters are important:

- *Transaminases as liver damage markers.* There is no international consensus on the degree of rise in ALT/AST which represents a usable liver. Current UK practice is to accept livers with a rise in ALT ≤ 500iu/L over 2 hours. Cases have been described in Italy where PNF occurred with the terminal ALT>1000iu/L.
- *Lactate as a function marker.* The lactate should fall over the course of two hours, but may not reach normal values due to venous return from the upper body and non-perfused limbs. Clamping the intrathoracic IVC may be associated with a greater fall in lactate measured in the circuit.

A routine **liver biopsy** before or after NRP (or both) is supported for quality assurance purposes where the NRP team feel it is appropriate. Biopsy sites in the liver should be sutured and noted on the A form

8.9.2 Pancreas

Amylase measured on the near patient analyser may indicate pancreatitis, but visual appearance is more useful.

8.9.3 Kidneys

Urine output falls off and may stop completely during NRP. There is no useful biochemical marker.

8.10 Post NRP

The appropriate paperwork should be filled in and a copy sent with each organ.

8.11 Failure to establish NRP

If NRP cannot be established, initial cold perfusion should follow the standard DCD protocol. If NRP is successful, cold perfusion should follow the DBD protocol. Back-table preparation should follow the current protocols.

9 Documentation

It is important that the perfusion characteristics during NRP, the blood gases, the fluids used and the timings, are all captured on the approved NHSBT NRP organ passport, and a copy sent

with each organ for the recipient surgeons. A copy should also be retained by the NOORS team and a further copy sent to NHSBT.

10 Troubleshooting NRP

10.1 Communication

Many of the issues occurring during NRP can be avoided by a good team brief and handover before the procedure so that everyone knows what is expected of them. This is particularly important when a cardiothoracic team is present.

10.2 Volume loss

Volume losses occur for the following reasons:

- Relaxation of alpha-adrenergic vasoconstriction occurring prior to death and loss of sympathetic tone;
- Bleeding, typically in the chest near the thoracic aortic clamp, or in the pericardium if lung and/or heart have been removed. In the abdomen it is often around the venous/arterial cannulae;
- Occlusion of the venous cannula, usually due to pressure or manipulation on the liver or bowel;
- Increasing the flow rate may reduce the reservoir volume.

Sudden loss of venous return can rapidly exhaust the circuit reservoir resulting in failure of the circuit: a one litre volume in the reservoir will disappear in 20 seconds at a flow rate of 3L/min. The perfusion practitioner should keep careful watch on the reservoir level, caution the surgeon and replenish the reservoir as the volume falls.

If volume is lost suddenly it may be because the wall of the cava has been sucked onto the cannula and occludes the holes due to manipulation of the organs; temporarily stopping organ manipulation, and manoeuvres such as reducing the height difference between donor and pump, or partial occlusion of the venous return tubing may also be effective. Alternatively, this is remedied by stopping the pump, waiting a couple of seconds, then restarting at a lower flow rate.

10.3 Air in the circuit

Air in the arterial limb can embolise and impair perfusion of organs. It is important to de-air the cannula and circuit when establishing the circuit at the beginning to prevent air entry. Small amounts of air in the venous side will run off into the open reservoirs of currently used circuits and cause no harm. Large volumes of air, as may occur if the cannula becomes dislodged, will cause an airlock which will block blood returning to the reservoir. This airlock needs to be walked along the tube by holding the tube distal to the airlock upwards to allow the tube to fill from below, and displace the air.

10.4 Clots in the circuit

A clot in the circuit can embolise into the organs. Fresh clot is most likely to occur first on large surface areas such as the oxygenator and leucocyte filter, and will impede flow. Clots in the circuit occurs for the following reasons:

- Failure to add any or sufficient heparin to the prime solution
- Failure to circulate heparin around the circuit before starting perfusion
- A long delay between connecting the venous cannula and draining blood into the reservoir, and starting NRP. Non-heparinised blood in the venous line will clot; that in the reservoir should not as it mixes with heparinised prime solution.
- Excessive bleeding requiring replacement with large volumes of non-heparinised fluids
- Pre-existing venous clots associated with lines

10.5 Troubleshooting Cs for poor flows

The following Cs are worth remembering when trouble-shooting (courtesy of James Richards):

- **C**annulas: check position, check vein not collapsing on cannula (if so increase volume)
- **C**lamps: check cross-clamp on thoracic aorta is on aorta, and all clamps on circuit released? Check position of supra-hepatic caval clamp
- **C**ava: are you compressing on it in your dissection or is it collapsing?
- **C**ircuit: anyone or anything compressing the circuit
- **C**hest: a common site for bleeding
- **C**lots: venous or atrial clots preventing venous return; clots on leucocyte filter or oxygenator
- **C**OLD: if you can't resolve issue in timely fashion then go cold and salvage the organs

11 Appendix 1 Protocol for direct recovery and perfusion of the heart and A-NRP

Due to the complexity of the procedure and the risk to the abdominal organs this combined technique is only appropriate for use where the heart is being retrieved for transplant purposes.

11.1 Cardiac team requirements for successful cardiac recovery

The following are required:

- Senior surgeon who is experienced in DCD heart retrieval
- A cell saver, to enable blood to be washed plus disposables
- The *ex situ* normothermic heart perfusion machine.
- Technician to operate the *ex situ* perfusion machine and the cell saver
- The necessary sterile tubing and adapters to connect to the NRP circuit ($^3/_8$ and ½ inch tubing).
- An appropriately staffed and equipped lung retrieval team if the lungs are also being retrieved

11.2 Abdominal team requirement for successful cardiac recovery during A-NRP

- Senior surgeon who is experienced in NRP
- The NRP disposable circuit
- NRP heater/cooler and pump (e.g. Cardiohelp)
- Experienced NRP perfusion practitioner
- 2 x long vascular clamps for descending aorta and IVC clamping (e.g. long straight and curved DeBakey)
- 2 Roberts clamps, one for the SVC and one for the ascending aorta

11.3 SNOD requirements

8 units of bank blood, 4 to be added to prime

11.4 Circuit

11.4.1 Preparation

The NRP circuit needs to have a Y attachment on the venous return limb just above the reservoir, and needs to be fitted prior to arrest if it is not already present on the circuit. This needs to be connected to the cell saver to allow for donor blood drainage needed for *ex situ* heart perfusion, but clamped initially.

11.4.2 Prime solution

- 4 units packed red cells (approx. 1200mls)
- 1.5 litre Hartmann's solution
- 50000 units heparin
- Phentolamine 5mg
- Pancuronium 12 mg – to prevent abrupt diaphragmatic contraction when phrenic nerve is divided which can cause distress to attending teams and host hospital staff.
- 1ml/kg 8.4% sodium bicarbonate (=1mmol/kg)

- 1gm Methyl prednisolone
- Fluconazole: 400 mg
- Antibiotics to be added once perfusion begins:
  - 200mg teicoplanin
  - 120mg gentamicin
  - 500mg metronidazole

Figure 1. Drainage of blood for priming the OCS device

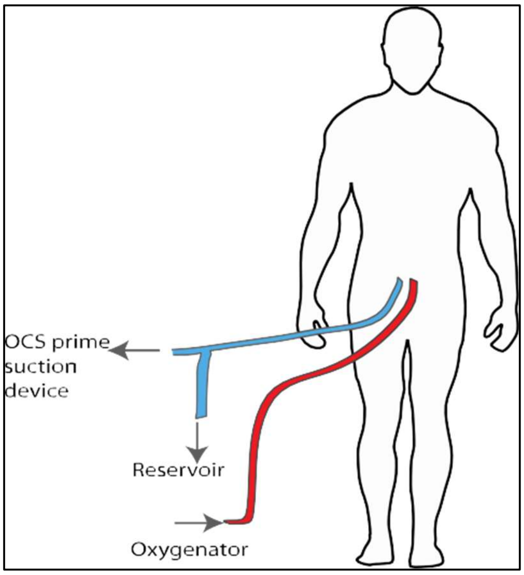

The NRP circuit is primed with 1.5 litres of Hartmann's, to which are added 4 units of red cells. The circuit needs to be set up before withdrawal of treatment, and warmed to 37°C by circulating through the oxygenator/heat exchanger.

Ideally, a pump sucker is connected to the reservoir for blood loss recovery during NRP, and the reservoir placed under negative suction. This is the preferred standard with teams working towards this, until then existing practice will prevail. This will only be used to recover blood from the pericardium if heart retrieval only, or to recover blood from pericardium and pleural space if combined heart-lung retrieval. Blood should not be recovered from the pleural space in the presence of chest sepsis. Additional care must be taken to avoid any perfusion fluid/saline being recovered using this sucker.

This additional sucker will not be used in cases of pericardial, mediastinal or systemic infection. Careful haemostasis should be performed in the chest even in the event of having a sucker available.

Two long DeBakey vascular clamps will be ready to use by the cardiothoracic team prior to WLST to clamp descending aorta and IVC. Two Roberts clamps will also be ready to clamp SVC and ascending aorta. It has been agreed that clamps will be provided by the abdominal team as they need to stay in place once the Thoracic team has left the operating theatre.

Due to the complexity of the technique, cardiothoracic organs will be retrieved only for transplantation purposes whilst the donor undergoes NRP. The heart for valves may be retrieved after NRP is concluded.

11.5 Operative procedure

Following verification of death 5 minutes after circulatory arrest, the patient is transferred to the operating table.

IT IS MANDATORY TO FOLLOW THIS STEP SEQUENCE

11.5.1 Abdominal team procedure

1. The circulating pump is stopped, and the sash is clamped and divided; the arterial cannula may be attached and primed at this point.
2. Once the donor is in theatre, the abdomen and right groin are prepared and draped.
3. The venous cannula is placed in the right common femoral vein common (or iliac vein or IVC) and connected to the venous limb of the sash, with care to exclude air. Care should be taken not to insert too much length of cannula to prevent it going into the right atrium.

   IF there is problem with achieving venous cannulation the thoracic team may choose to cannulate the let atrial appendage; this cannula should be removed and the appendage ligated before starting NRP or else air will be entrained in the circuit and NRP fail. For this reason atrial cannulation is a last resort.
4. Clamps are removed and 1.5L venous blood drained out and diverted into the collecting receptacle for the heart Organ Care System (OCS) (such as the cell saver system used by Harefield).
5. The Y-connector is then clamped and venous return blood now diverted to drain back into the reservoir (see figure 1)
6. The arterial cannula is placed in the right femoral artery, common iliac artery or aorta while the venous drainage occurs.
7. Once the cardiac team have clamped the descending thoracic aorta and stated that clearly for both teams to hear, and the 1.5L venous OCS prime has drained, the NRP pump is started aiming for flows over 2.5L/min. The time that the descending thoracic aorta is clamped will be recorded on the National DCD Heart Passport.

Abdominal NRP must not start until both teams have confirmed for all to hear that the descending aorta is clamped.

Once the heart is removed it is important to check the security of the supra-hepatic IVC clamp – this may need to be sutured in place to avoid inadvertent unclamping or slipping from the cut IVC. The cut ends of the pulmonary vessels and SVC may be oversewn with 3/0 Prolene at this stage also. While the cardiac surgeons should ensure haemostasis in the chest, in reality it is the abdominal surgeons who are usually free at this stage and can stop large vessel bleeding.

There should be no major bleeding.

11.5.2 Cardiac procedure

The chest is opened in the midline and sternum split while the abdomen or groin is opened for cannulation. The cardiothoracic team will apply a clamp across the descending thoracic aorta, and announce to theatre when this has been done. They will then place a DLP cannula, open to air, as vent in the ascending aorta to monitor for possible brain perfusion

Once the DLP cannula is in place and open to air, the cardiothoracic surgeon announces that the aortic arch is vented, at which point the NRP pump may begin. The time will be recorded on the National DCD Heart Passport. If there is copious arterial bleeding from the DLP cannula, the NRP pump must stop and the clamp on the descending aorta must be re-positioned to occlude the aorta. Only then can the NRP pump re-start.

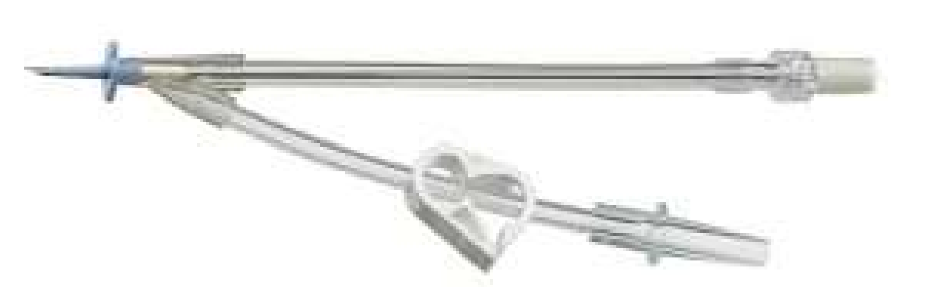

Figure 2. DLP cannula

The details of heart and lung retrieval are in the separate cardiothoracic protocol for DCD donors "*Direct retrieval and perfusion (DPP) of DCD heart and lungs with or without A-NRP to ex situ normothermic perfusion*" at odt.nhs.uk microsite

11.5.3 Procedure for lung retrieval alone without heart while on A-NRP

See separate protocol for "*Direct retrieval and perfusion (DPP) of DCD heart and lungs with or without A-NRP to ex situ normothermic perfusion*" at odt.nhs.uk microsite.

The thoracic team will stand back for 30 minutes after cold in-situ perfusion of the lungs. Dissection of the lungs will then begin, which can be assisted by the abdominal surgeons as appropriate. This should be carried out with care and detail, to enable stability to be established on A-NRP, and to maintain stability during and after the lungs have been removed.

12 Appendix: UK NRP implementation group members involved in drafting the protocol

Marius Berman	Co-chair, novel technologies implementation group and Consultant Surgeon, Royal Papworth Hospital, Cambridge
Andrew Butler	Consultant Surgeon, Addenbrookes Hospital, Cambridge
Ian Currie	Clinical lead for Organ Retrieval, NHSBT and Consultant Surgeon, Edinburgh Transplant Centre
Diana García Sáez	Consultant Surgeon, Harefield Hospital
Fiona Hunt	Advanced Perfusion and Organ Preservation Specialist, Edinburgh Transplant Centre
Mubbasher Husain	Consultant Surgeon, Harefield Hospital
Satheesh Iype	Consultant Surgeon, Royal Free Hospital, London
Anand Jothidasand	Consultant Surgeon, Harefield Hospital
Debbie Macklam	Senior Commissioning Manager, NHSBT
Gabriel Oniscu	Consultant Transplant Surgeon, Edinburgh Transplant Centre
Ulrich Stock	Professor and Consultant surgeon, Harefield Hospital
Chris Watson	Chair. Also Co-chair, novel technologies implementation group and Professor of Transplantation, Cambridge

UK National Protocol for direct retrieval and perfusion (DRP) of DCD Hearts and Lungs with or without abdominal NRP (A-NRP) to Ex-situ Normothermic perfusion

Version number 2
Date: 1 November 2021

Responsible author; Marius Berman, co-chair of the Novel Technologies Implementation Group

Diana García Sáez, Mubbasher Husain, Anand Jothidasan, Ulrich Stock, Vipin Mehta, Rajamiyer Venkateswaran, Phil Curry, Majid Mukadam, Jorge Mascaro, Stephen Clarke, Jen Baxter, Simon Messer, Steven Tsui, Stephen Large, Pradeep Kaul, Mohamed Osman, Antonio Rubino, Marian Ryan, Jacob Simmonds, Julie Whitney, Dale Gardiner, Chris Watson, Ian Currie, Debbie Macklam, Jeanette Foley, Marius Berman

DGS, MH, AJ, US – Harefield Hospital

VM, RV – Wythenshawe Hospital

PC – Golden Jubilee National Hospital, Glasgow

MM, JM – Queen Elizabeth hospital, Birmingham

SC – Freeman Hospital, Newcastle

SM, ST, SRL, PC, MO, PK - Royal Papworth

AR – Regional CLOD, DCD JIF working group 3

MR – Regional manager, ODT NHSBT

JS – Great Ormond Street Hospital

JB – Novel Technology Implementation Group – OCS operator

JW – Head of Service Delivery, ODT Hub

DG – UK National Clinical Lead for Organ Donation, NHSBT

CW – Chair, abdominal NRP working group

IC – UK Lead Organ Retrieval, NHSBT

DM – Head of Service Development – ODT NHSBT

JF – Head of Clinical Governance, NHSBT

MB – Associate UK Lead Organ Retrieval, NHSBT

Preface

This protocol was produced by a combination of clinicians covering donation, organ retrieval and transplantation, NHSBT and acquired experience for the past years. It is acknowledged that some of the details might have local variation, but, this is the overall framework we recommend to adhere to.

Index:

DCD Heart Donor selection	4
DCD Heart Assessment	5
Withdraw of life sustaining treatments	5
Functional warm ischaemia and stand down criteria	7
Surgical Protocol – NO ABDOMINAL NRP	9
Surgical Protocol – WITH ABDOMINAL NRP	13
Set up of Transmedics OCS and use of Cell Saver	21
OCS perfusion parameters during transport and Final decision	21
Safety check list	22
Scrub Trolley discipline	23
CT surgeons synchrony	26

Protocol amendments

1. Version 1.0, date 24/03/2021 Final version approved
2. Version 1.1, date 01/04/2021 Updated clarification re DCD Heart Assessment, Section 2; minor typos
3. Version 2. Date 1/11/2021;
    - Delete NRP priming and refer to updated relevant protocol
    - Open OCS AFTER assessment for coronary artery disease p 8
    - Reference to Papworth OCS and Cell Saver training manuals p21
    - OCS perfusion parameters and final acceptance criteria. p21
    - Diagram of OCS management - deleted

1. DCD HEART DONOR SELECTION

Donor Inclusion Criteria

- Controlled DCD (Maastricht Category 3 and 4)
- Age ≤ 50 years
- Weight ≥ 50 Kg.
- Weight ≥30 kg – if suitable paediatric recipient at GOSH or Newcastle, discuss directly with Papworth on call retrieval consultant. Refer to DCD paediatric protocol. Protocol will be updated in future once perfusion technology available for < 30kg donors.
- Consent/authorisation obtained from next of kin/ organ donor register

Donor Exclusion Criteria

- Previous cardiac surgery
- Previous midline sternotomy
- Valvular heart disease
- Congenital heart disease
- Significant coronary artery disease
- Chronic atrial fibrillation
- Insulin dependent diabetes
- Virology: HIV+
- Current IV drug abuse.
- Tumour with high risk of transmission according to SABTO guidelines

NORS team Mobilisation

- Cardiac NORS team to arrive up to 2 hours before the planned withdrawal of treatment time
- Abdominal team to arrive 1 hour before withdrawal of treatment time.
- If NRP being used, both teams must arrive at the same time as the Cardiac team (2 hours before planned withdrawal time)

2. DCD HEART ASSESSMENT

- A transthoracic Echocardiogram (TTE) will be performed for all donors and be available at the time of the offer. All efforts should be made to transfer the images for review by the implanting team prior to mobilization of the NORS DCD Heart team. If that is not possible, the retrieval team will review the images (only if they are available on arrival at the donor hospital) and communicate with the implanting team prior to withdrawal of life sustaining treatment (WLST).
- If there are no images available but a full detailed TTE report is available, the retrieval should proceed if the recipient centre is happy with the findings.
- If an echocardiogram has not been performed prior to offering, there should be no more than 3h delay in performing and conveying the results.
- If no formal echo available, explore if the ICU team will be willing to perform a Focused Cardiac Ultrasound (FCU – previously known as FICE) though this is not mandatory for the ICU. This will serve as a screening step.
- Formal TTE will be performed by donor hospital, or, trained member of the retrieval team. NO TOE (transoesophageal) echocardiography will be performed at any stage!
- If no FCU or TTE is available, heart should still be offered for transplantation. It is the responsibility of the lead transplanting surgeon to discuss with the lead retrieval surgeon regarding the offer without an ECHO. The decision to accept or decline must be made within the standard offering timeframe.
- Echo main criteria: EF > 50%, no valvular pathology, PW and/ or IVS < 15mm

DCD HEART Withdrawal of life sustaining treatments

- Withdrawal of life sustaining treatments should ideally be undertaken in the anaesthetic room / theatre complex by the local hospital intensive care team.
- If it is not local practice to withdraw in the anaesthetic room / theatre complex then it may need further discussion between retrieval and donor hospital teams, aiming to withdraw support as close to theatre as possible, in order to minimize the ischaemic time during transfer to theatre. The place of withdrawal should be agreed before the NORS team is mobilised to avoid disagreement at retrieval.

- Height of donor table should be as the same as theatre table. This is done simply by marking the height of the donor bed by tape of the SNOD trousers and match this with the theatre table height.

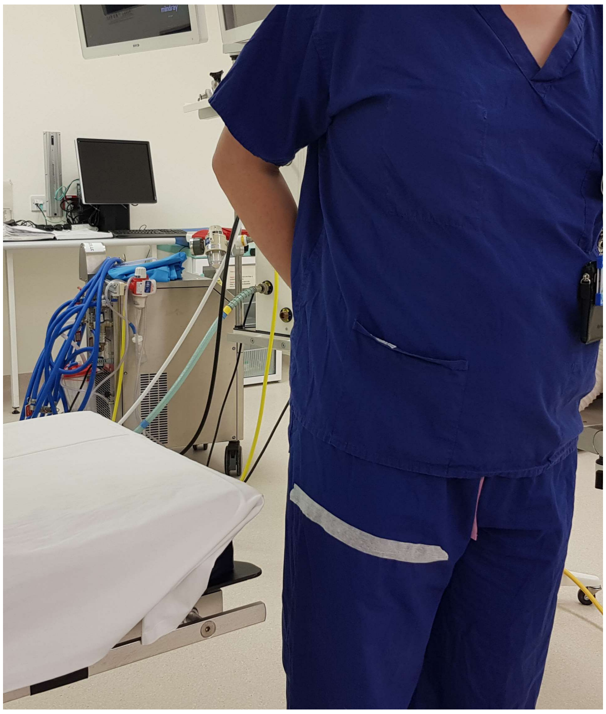

- SNOD – If Heart is not suitable for transplantation, please explore pathway for research approved project or valves.
- **It is recommended that the donor is transfused to Hb of >= 100 g/L.** Timing of transfusion - once CT NORS team is mobile.

- **Heparin in the donor before circulatory arrest**

Maastricht 3 donors: No pre-mortem interventions are currently allowed in the UK

Maastricht 4 donors (donor is already certified dead by brain stem criteria): heparin can be given. A suggested dose is 300units/kg (around 25000 units for a 80kg person) given just prior to withdrawal of treatment

- **SNOD – prepare units of packed red blood cells (cross matched to donor) :**
  - **4 units – no NRP**
  - **8 units - abdominal NRP**

DCD HEART Functional Warm ischaemia and Stand Down Criteria

- After withdrawal of treatment, regular contact will be maintained with the SNOD regarding blood pressure and arterial saturations on the donor.
  - When Harefield is implanting team and the ICU team in the donor hospital are agreeable; once arterial saturation is <80% an arterial blood gas should be taken to confirm donor hypoxia, according with local hospital policy.
  - Functional warm ischaemia begins when systolic blood pressure falls below 50mmHg.
  - 30 minutes from beginning of functional warm ischaemia until cold cardioplegia is delivered will be tolerated before standing down.
  - Essential for the team diagnosing death to be familiar with the Academy of Medical Royal Colleges 2008 Code of Practice for the Diagnosis and Confirmation of Death.
  - If cardiac arrest does not occur within 120 minutes from withdrawal of treatment, consider standing down DCD heart retrieval at this stage, unless death is likely to be imminent
  - We recommend having a discussion between retrieval and recipient centres after 60 min from withdrawal.

If the donor meets criteria the OCS module should be opened at that point and the priming process started

Transfer to operating theatre

- When the donor is brought into the operating room, the SNOD shows the patient name band to confirm donor identity. If withdraw adjacent to operating theatre, if SNOD and both NORS teams are in agreement, consider confirming demographics and name band prior to withdrawal.

- **ONLY IF LUNG RETRIEVAL IS TAKING PLACE AS WELL.** Once the donor is transferred to the operating table, an endotracheal tube size above 8 is inserted. At a point no earlier than 10 minutes after the onset of mechanical asystole, the lungs are re-inflated with a single breath of oxygen-enriched air. Lung ventilation

will commence once ascending aorta is clamped, as per described in the National Standards for Organ Retrieval.

- The thoracic and abdominal surgeons will prepare the skin with an alcohol-based skin preparation solution and apply 4 drapes.
- A midline sternotomy is performed with a retractor to spread the sternal tables placed upside down. The abdominal surgeon will open the abdomen simultaneously.

Composition of the DCD CT Retrieval Team

- A theatre practitioner scrubbing for organ retrieval
- Organ preservation practitioner (OPP)
- Advanced Perfusion and Organ Preservation Specialist (APOPS)
- Two surgeons, at least one has been accredited as competent DCD cardiothoracic organ retrieval
- If aNRP, it is recommended to seek additional surgeon from Harefield or Papworth

For all retrievals, First do assessment for coronary disease and only afterwards, give clear instruction to open and prime the OCS.
The assessment can take place while draining blood to prime the OCS.

3. Surgical protocol – NO ABDOMINAL NRP

Preparation

- Prepare St Thomas cardioplegia - Add the following medication to 500ml bag of Ringers:

    - 2,500iu of Epoetin Alfa
    - 50mgs GTN
    - 3mls Sodium bicarbonate 8.4% (840mgs in 10ml amp)
    - 10mls cardioplegia concentrate
    - Add heparin 300u/kg

    (Solution to be put back into the ice box but easily accessible for use when donor arrives in theatre)

- Prepare St Thomas cardioplegia for back at implant site – Add the following medication to 1L bag of Ringers:

    - 5000iu of Epoetin Alfa
    - 100mgs GTN
    - 6mls Sodium bicarbonate 8.4% (840mgs in 10ml amp)
    - 20mls cardioplegia concentrate

    (Solution to be put back into the ice box for use when heart is at implant site))

- Blood collection – see also Appendix 2: Minimum of 1.2-1.5L to be collected with a raised table in head down position. It is crucial to ensure that no preservation solution is given until donor blood is drained, and no vasoconstrictor bolus is given at this stage. This should take no more than 60secs.
  There are several variations across units;
    - Insertion of a 2-stage venous cannula connected to a blood collection bag with Heparin 25,000 IU. If this is the case, 25,000 IU of heparin are injected into the right atrium and 25,000 IU of heparin into the pulmonary trunk prior to blood drainage.

    - Insertion of a drainage cannula connected to a sucking device or sterile reservoir. Blood is drained under suction and simultaneously mixed with the

OCS priming solution containing 60000 IU of heparin

- During donor blood collection the cardiothoracic surgeon will clamp the descending aorta above the diaphragm, as low as possible. The cardiothoracic surgeon will announce this clamp is in place and the time will be recorded on the National DCD Heart Passport.

- A clamp is placed across the ascending aorta and a DLP cannula inserted into the ascending aorta for cardioplegia delivery and the heart excised in the standard fashion for heart retrieval.

- If the lungs are to be retrieved, the local hospital anaesthetist or the NORS team donor care practitioner will reintubate the donor during sternotomy as per DCD Lung retrieval.
    - Care must be taken to leave the posterior wall of PA carina when removing the heart. As soon as the heart is removed, ante-grade pneumo-plegia is delivered through these cannulae followed by retrograde pneumo-plegia via the pulmonary veins. Fibre optic bronchoscopy is performed and lungs are retrieved in standard fashion for DCD lung retrieval.

Preparation of the DRP-DCD heart prior to Ex-Situ perfusion

- The heart is immediately placed into a basin of ice cold sterile saline solution.

- Dissection made to free the aorta from the pulmonary artery placing and securing the appropriately sized perfusion connector for the Organ Care System (OCS) with the supplied cable tie. Teflon pledgeted aortic stitches are used to further secure the aorta to the OCS so reducing the risk of disconnection during travel to the recipient hospital.
- The heart is placed and de-aired onto the primed OCS.

- Insert and secure LV vent through the left atrium into organ chamber.

Place ventricular pacing wires in case pacing is required at a later stage.

PA cannula (Protocol difference)

Harefield implanting or retrieval – PA cannula secured and connected. (SVC and IVC - sutured) and connect blue flow probe – follow Transmedics protocol.

Manchester/Papworth retrieval – PA cannula NOT connected, allowing free drainage.

Three teams agreed cross over protocol at JiF DCD meeting on 28/1/2020

OCS perfusion parameters during transport:

Commence OCS perfusion of donor heart aiming for:

- Mean AOP 55-70 mmHg
- Aortic flow of 900-1100 mL/min-
- Coronary flow 650-750 ml/min
- Heart rate 70-90 BPM with V-pacing
- Once heart rhythm and perfusion are stable consider to synchronise perfusion depending on discussion with implanting team.

Acquire simultaneous AV blood samples. Perfusate targets are:

- Hct >15%
- Calcium 1.0-1.3 mmol/l
- Bicarbonate 22-29 mmol/l
- Ph – 7.3-7.45

Video clip to be transferred to implanting center at 30min reperfusion on the rig

Transport

Ensure to travel with a safety ice box and roadside bag which will include;

- Ice, cardioplegia, giving set + pressure bag, 8 litres of cold saline

Roadside bag – sterile instruments, sterile gloves different size, sterile gowns, 3 packing bags for heart.

Cardioplegia at recipient site (agreed telecom 2.9.20)

Once implanting team are happy to receive the heart,

- The retrieval team have set up to administer cardioplegia.
- All 3 teams will administer St. Thomas at retrieval and implant site when retrieving for any 6/7 UK centers.
- Harefield will carry both Custadiol and St. Thomas and will have a choice of Custadiol or St. Thomas when retrieving for Harefield.

(Refer to St. Thomas preparation on page 7)

Trouble shooting;

- Check placement heart on the rig (twist, impaired drainage..)
- Syringe drives
- Flow probes and sensors
- Module position within the rig
- Redo medication preparation

4 Surgical protocol – WITH ABDOMINAL NRP

For the most up to date version, please refer to;

https://www.odt.nhs.uk/retrieval/policies-and-nors-reports/

Circuit
The NRP circuit needs to have a Y attachment on the venous return limb just above the reservoir, and needs to be fitted prior to arrest. This needs to be connected to the cell saver to allow for donor blood drainage needed for *ex situ* heart perfusion, but clamped initially.

-

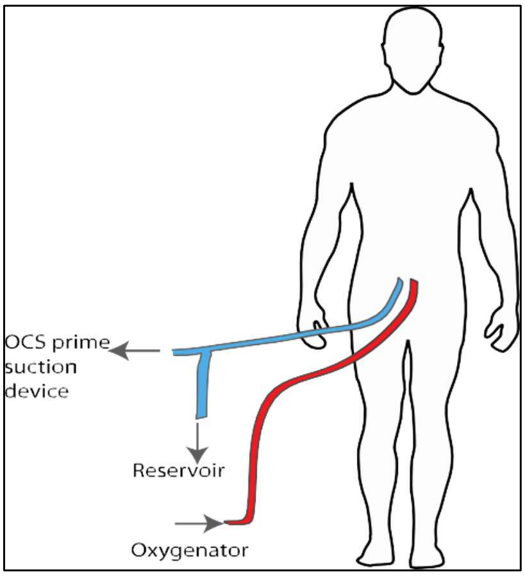

The NRP circuit is primed with 1.5 litres of Hartmann's, to which are added 4 units of red cells. The circuit needs to be set up before withdrawal of treatment, and warmed to 37°C by circulating through the oxygenator/heat exchanger.

A pump sucker will be connected to the reservoir for blood loss recovery. (This is the preferred standard with teams working towards this, until then existing practice will prevail). This will only be used to recover blood from the pericardium if heart retrieval only, or to recover blood from pericardium and pleural space if combined heart-lung retrieval. Blood should not be recovered from the pleural space in the presence of chest sepsis. Additional care must be taken to avoid any perfusion fluid/saline being recovered using this sucker.

THIS ADDITIONAL SUCKER WILL NOT BE USED IN CASES OF PERICARDIAL, MEDIASTINAL OR SYSTEMIC INTECTION. CAREFUL HAEMOSTASIS SHOULD BE PERFORMED IN THE CHEST EVEN

IN THE EVENT OF HAVING A PUMP SUCKER AVAILABLE.

Two long DeBakey vascular clamps will be ready to use by the cardiothoracic team prior to WLST to clamp descending aorta and IVC. Two Roberts clamps will also be ready to clamp SVC and ascending aorta. It has been agreed that clamps will be provided by the abdominal team as they need to stay in place once the CTh team has left the operating theatre.

Due to the complexity of the technique all cardiothoracic organs will be perfused and retrieved only for transplantation or valve donation purposes.

Operative procedure

Following verification of death 5 minutes after circulatory arrest, the patient is transferred to the operating table.

IT IS MANDATORY TO FOLLOW THIS STEP SEQUENCE

Abdominal procedure

1 The circulating pump is stopped, and the sash is clamped and divided; the arterial cannula may be attached and primed at this point.

2 Once the donor is in theatre, the abdomen is opened through a midline incision.

3 The venous cannula is placed in the right common femoral vein (or iliac vein or IVC) and connected to the venous limb of the sash, with care to exclude air. Care should be taken not to insert too much length of cannula to prevent it going into the right atrium.

IF there is problem with achieving venous cannulation the thoracic team may choose to cannulate the right atrial appendage; this cannula should be removed and the appendage ligated before starting NRP or else air will be entrained in the circuit and NRP fail. For this reason atrial cannulation is a last resort.

4 Clamps are removed and 1.5L venous blood drained out and diverted into the collecting receptacle for the heart Organ Care System (OCS) (such as the cell saver system used by Harefield).

5 The Y-connector is then clamped and venous return blood now diverted to drain back into the reservoir (see figure 1). Please ensure having the correct connectors – 3/8, and ½.

6 The arterial cannula is placed in the right femoral artery, common iliac artery or aorta while the venous drainage occurs.

7 Once the cardiac team have clamped the descending thoracic aorta and stated that clearly for both teams to hear, and the 1.5L venous OCS prime has drained, the NRP pump is started aiming for flows over 2.5L/min. The time that the descending thoracic aorta is clamped will be recorded on the National DCD Heart Passport.

Abdominal NRP must not start until both teams have confirmed for all to hear that the descending aorta is clamped and aortic arch is vented via a DLP cannula.

8 Once the heart is removed it is important to check the security of the supra-hepatic IVC clamp – this may need to be sutured in place to avoid inadvertent unclamping or slipping from the cut IVC. The cut ends of the pulmonary vessels and SVC may be oversewn with 3/0 Prolene at this stage also. While the cardiac surgeons should ensure haemostasis in the chest, in reality it is the abdominal surgeons who are usually free at this stage and can stop large vessel bleeding.
There should be no major bleeding.

Heart Retrieval

The chest is opened in the midline and sternum split while the abdomen is being opened.
Pericardiotomy

Heart retrieval only

1. The left pleural space is opened and DESCENDING THORACIC AORTA IS CLAMPED above the diaphragm to isolate abdominal NRP. Priority will be given to ensure absence of brain reperfusion via NRP system. The act of clamping the descending aorta should be announced loud enough for all to hear and the time will be recorded on the National DCD Heart Passport

2. PLACEMENT OF DOUBLE LUMEN DLP CANNULA IN THE ASCENDING AORTA, as high as possible. Initially, used to drain the ascending aorta blood. Later in the sequence, it can be used for cardioplegia delivery.

   Once the DLP cannula is in place and open to air, the cardiothoracic surgeon announces that the aortic arch is vented. The time will be recorded on the National DCD Heart Passport. If there is copious arterial bleeding from the DLP cannula, the NRP pump must stop and the clamp on the descending aorta must be re-positioned to occlude the aorta. Only then can the NRP pump re-start.

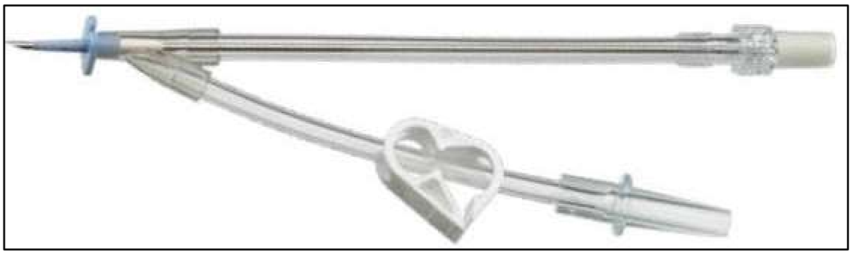

3. The SVC and azygous vein are dissected to ensure enough length.

4. The IVC is dissected around. If the tip of the cannula is inside the right atrium, the abdominal team should be asked to pull the cannula back below diaphragm to allow for IVC clamping at a later stage. Check to ensure the venous cannula does not encroach into the right atrium.

The heart is assessed for any visible anomalies, palpable coronary artery disease, left ventricular hypertrophy, trauma, congenital disease etc.

5. Once 1.3-1.5L of donor blood has been received into the receptacle / cell saver for the OCS prime, CLAMPS ARE PLACED ACROSS THE IVC ABOVE THE DIAPHRAGM, AND THE SVC *CAUDAL* TO THE AZYGOS. The SVC is transected caudal to clamp, placed below azygos vein.
6. The ascending aorta is clamped, in addition to the descending thoracic aortic clamp.

7. IVC is opened just cranial to the clamp for venting and left atrium is opened at level of pulmonary veins for pulmonary return.

8. Cardioplegia supplemented with 25000 IU heparin, EPO and 50 mg of GTN is administered via a large bore needle PROXIMAL to the cross clamp. The previously placed DLP cannula, distal to the cross clamp, at the level of the arch will remain in situ and open to air.

9. Once cardioplegia is finished, the large bore cannula is removed.

10. The heart is then excised leaving all previously placed clamps in situ to minimize blood loss.

11. Establish with the abdominal team and identify team member who is going to secure potential bleeding points. – stich 3/0 stump IVC (with or without the clamp), SVC, azygos and pulmonary veins.

12.The heart graft is prepared at the back table and re-perfused with ex situ normothermic perfusion technology in the usual manner.

Heart and Lung retrieval

Once the donor is transferred to the operating table, an endotracheal tube size above 8 is inserted. At a point no earlier than 10 minutes after the onset of irreversible asystole, the lungs are re-inflated with a single breath of oxygen-enriched air. Lung ventilation will commence once ascending aorta is clamped, as per described in the National Standards for Organ Retrieval. Bronchoscopy performed usually at this point if an additional surgeon is available, or later after pneumoplegia completion.

1. The left pleural space is opened and DESCENDING THORACIC AORTA IS CLAMPED. The act of clamping the descending aorta should be announced loud enough for all to hear and the time

will be recorded on the National DCD Heart Passport.

2. Placement of double lumen DLP cannula in the ascending aorta, and cannula opened to air; This cannula is used initially to ensure absence of brain perfusion and later used for cardioplegia delivery.
Once the DLP cannula is in place and open to air, the cardiothoracic surgeon announces that the aortic arch is vented. The time will be recorded on the National DCD Heart Passport. If there is copious arterial bleeding from the DLP cannula, the NRP pump must stop and the clamp on the descending aorta must be re-positioned to occlude the aorta. Only then can the NRP pump re-start.

3. SVC, IVC dissection and donor blood drainage, as per heart only retrieval technique, is performed.

4. Clamps are placed across the IVC above the diaphragm and the SVC caudal to the Azygos. The IVC is opened just proximal to the clamp for venting and the left atrial appendage is vented widely.

5. The ascending aorta is clamped, proximal to DLP cannula, only during cardioplegia delivery. Cardioplegia supplemented with 25000 IU heparin, EPO and 50 mg of GTN is administered via a large bore needle PROXIMAL to the cross clamp. The previously placed DLP cannula, distal to the cross clamp, at the level of the arch will remain in situ and open to air.

6. Once cardioplegia is finished, the large bore cannula is removed.

7. After cardiectomy, antegrade pneumoplegia is completed according to National protocol. Simultaneously, the pleurae are opened widely and lungs inspected and palpated, ensuring adequate delivery of flush and topical cooling with copious volumes of 4°C saline.

8. If significant collateral flow from pulmonary veins, consider delivering retrograde pneumoplegia into the pulmonary veins

9. After completion of antegrade pneumoplegia, wait for aNRP to reach 30 min, prior to starting dissection.

10. After 30 min, inform everyone in theatre that CT surgeons start lung dissection.

11. If the lungs are suitable and accepted for transplantation a competent lung retrieval surgeon will complete rest of dissection while abdominal NRP continues, by dividing the descending thoracic aorta and taking this along with the lung bloc. This would involve ligaclipping all the intercostal arteries (L and R) up to the arch, being careful to avoid bleeding.

The care and detail required to retrieve lungs whilst NRP is running is the same as would be required in a living patient. The abdominal organs may be lost if the lung retrieval is performed in haste. If we are to build a future with novel technologies, both teams need to support

maximal organ retrieval and utilization.

Consider applying a second clamp on the descending thoracic aorta, just distal to left subclavian artery, in order to minimize bleeding. We recommend removing the lung block with the thoracic aorta, however, some might feel more comfortable dissecting in front of the aorta, in particular if there is no prospect of using EVLP. Need to bear in mind the left pulmonary artery when dissecting in this plane.

12. **The azygos vein must be ligated twice and cut in between**. This can be done easily in the right pleural space.
The rest of the lung dissection can be completed with diathermy and by using surgical Liga-clips aiming to minimize blood loss.

13. The trachea is stapled and cut leaving a clamp or staple line on the top end.

14. Retrograde pulmonary venous flush of the lungs is performed on the back-table at the donor site and lungs are packed as per National protocol.

Lung retrieval only

Once the donor is transferred to the operating table, an endotracheal tube size above 8 is inserted. At a point no earlier than 10 minutes after the onset of irreversible asystole, the lungs are re-inflated with a single breath of oxygen-enriched air. Lung ventilation will commence once ascending aorta is clamped, following all steps as per the National Standards for Organ Retrieval.
Bronchoscopy performed usually at this point if an additional surgeon is available, or later after pneumoplegia completion.

1. The left pleural space is opened and DESCENDING THORACIC AORTA IS CLAMPED.
   The act of clamping the descending aorta should be announced loud enough for all to hear and the time will be recorded on the National DCD Heart Passport.

2. Placement of double lumen DLP cannula in the ascending aorta to ensure absence of brain perfusion
   Once the DLP cannula is in place and open to air, the cardiothoracic surgeon announces that the aortic arch is vented. The time will be recorded on the National DCD Heart Passport. If there is copious arterial bleeding from the DLP cannula, the NRP pump must stop and the clamp on the descending aorta must be re-positioned to occlude the aorta. Only then can the NRP pump re-start.

   Place pnemoplegia cannula into the PA.

   Clamp proximal PA .

Cut LA appendage.

3. Antegrade pneumoplegia as per UK National guidelines is administered. Simultaneously, the pleurae are opened widely and lungs inspected and palpated, ensuring adequate delivery of flush and topical cooling with copious volumes of 4°C saline.

   CT WILL WAIT after delivering antegrade pneumoplegia to complete 30min aNRP perfusion prior to carry on any further dissection. CT NORS might choose to use this time to repeat FOB or assess in more detail the lungs.

   THIS WILL ALLOW ESTABLISH OF aNRP flows for at least 30min, period crucial to liver recovery. Risk of bleeding is minimal at this stage.

4. Ascending aorta is clamped proximal to DLP cannula, and cannula should be open to air to ensure absence of brain perfusion.

5. SVC, IVC dissection is performed. Clamps are placed across the IVC above the diaphragm and the SVC caudal to the Azygos. The IVC is opened just proximal to the clamp for venting and the left atrial appendage is vented widely.

6. Heart -lung retrieval will be carried en-bloc. This is to minimize potential catastrophic blood loss.

7. Cardiectomy performed leaving **a long IVC cuff above previously placed IVC clamp**. Ascending aorta and SVC are both cut caudal to clamps, which stay in place to avoid bleeding.

8. If the lungs are suitable and accepted for transplantation the rest of dissection will be completed while abdominal NRP continues, being careful to avoid bleeding.

   The care and detail required to retrieve lungs whilst NRP is running is the same as would be required in a living patient. The abdominal organs may be lost if the lung retrieval is performed in haste. If we are to build a future with novel technologies, both teams need to support maximal organ retrieval and utilization.

9. **The azygos vein must be ligated twice and cut in between.** This can be done easily in the right pleural space.

10. The rest of the heart-lung bloc dissection can be completed with diathermy and by using surgical Liga-clips aiming to minimize blood loss.

11. The trachea is stapled and cut leaving a clamp or staple line on the top end

12. Secure major remaining stumps – IVC, SVC, arch vessels and any other source of bleeding.

13. Retrograde pulmonary venous flush of the lungs is performed on the back-table at the donor site and Lungs are packed as per National protocol.

14. Heart to be returned into the chest and document this action.

The cardio-thoracic surgeon should ensure haemostasis in the chest during and at the end of retrieval, before leaving the donor hospital. Excess bleeding may result in an unusable liver, pancreas and kidneys.

Requirements to undertake DRP and NRP

The following are required for the successful removal of the heart during NRP

From the cardiac team

- Senior surgeon who is experienced in DRP retrieval
- The *ex situ* normothermic heart perfusion machine. Technician to operate the *ex situ* perfusion machine and the cell saver
- The necessary sterile tubing and adapters to connect to the NRP circuit (3/8 and ½ inch tubing). An appropriately staffed and equipped lung retrieval team if the lungs are also being retrieved

From the abdominal team

- Senior surgeon who is experienced in NRP
- The NRP disposable circuit
- NRP heater/cooler and pump (e.g. Cardiohelp)
- Experienced NRP perfusion practitioner
- 2 x long vascular clamps for descending aorta and IVC clamping

Set up of Transmedics OCS and use of Cell Saver

Papworth have developed an OCS training manual for DCD hearts and an OCS blood collection with cell saver manual. These are available for reference on the NHSBT ODT microsite here [Policies and NORS reports - ODT Clinical - NHS Blood and Transplant]

OCS perfusion parameters during transport:

In general, it is recommended to maintain the OCS in manual rather than automatic mode.

Changes to flow and pacing have an immediate effect whereas changing the infusions of epinephrine or maintenance fluid may take minutes to take effect.

Commence OCS perfusion of donor heart aiming for:
- AOP: 55-70 mmHg
- Aortic flow: 800-1100 mL/min
- Heart rate: 70-90 BPM with V-pacing
- Aim CF: 650-750 ml/min
- Once heart rhythm and perfusion are stable consider synchronising perfusion depending on discussion with implanting team.

Acquire simultaneous AV blood samples. Perfusate targets are:
- Hct: >15%
- Calcium: 1.0-1.3 mmol/l
- Bicarbonate:22-29 mmol/l
- pH: 7.30-7.45

Parameters to consider prior to final decision

- **AOP 55-75 mmHg with Maintenance fluid <30 ml/Hr**
- **Aortic flow 800-1100 ml/min**
- **HR**
- **Total lactate trend decreasing over time**
- **Lactate consumption profile i.e. Lac_{Art} > Lac_{Ven}**
- **Contractility**
- **Prescence of superficial petechia and/or oedema**
- **FWIT < 30min i.e. Time from SBP<50mmHg to start of OCS perfusion**
- **OCS perfusion time + all the above + predicted preparation of implant (for example; if OCS > 4hours and redo surgery with predicted additional 2-2.30 hours OCS perfusion) need to asses all the above real time**
- **If in doubt, call on-call retrieval consultant surgeon at Royal Papworth Hospital for advice**

SAFETY CHECKLIST FOR DIRECT RETRIEVAL OF THE HEART/ HEART AND LUNGS AND *IN SITU* NORMOTHERMIC REGIONAL PERFUSION OF THE ABDOMINAL ORGANS

TO BE COMPLETED AT HANDOVER	***CTH SURGEON***	***ABDO SURGEON***
1 Protocol reviewed prior to WLST	☐	☐
2 Debrief completed prior to WLST	☐	☐
3 CTh team equipment ready	☐	
(Cell saver, Clamps, OCS, Fluids for perfusion)		
4 Abdominal team equipment ready		☐

Leading surgeon; Full name and signature

TO BE COMPLETED PRIOR TO START ABDOMINAL NRP

(Time to be noted and signed by Abdominal team Perfusionist)

1 Descending Aorta x clamp time

Scrub trolley discipline:

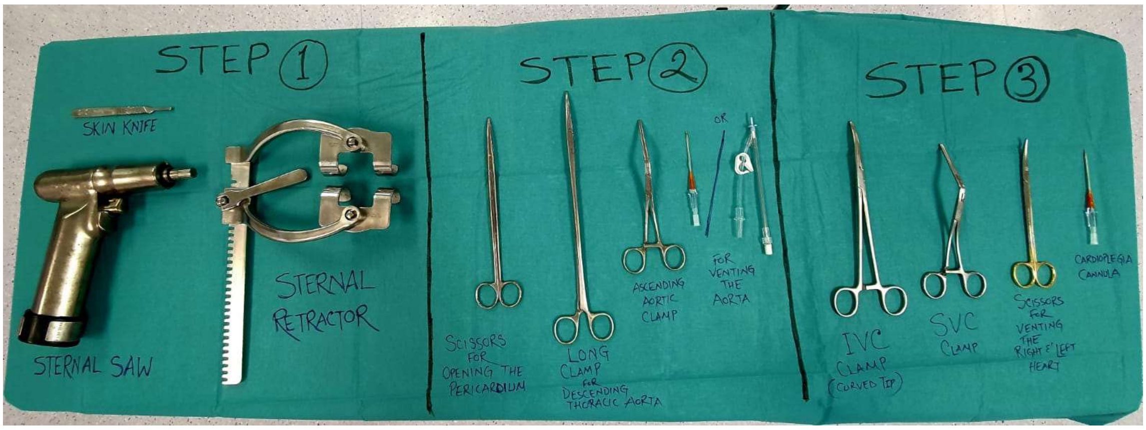

The above figure demonstrates the scrub trolley discipline, which correlates with the surgical steps, and this can be very helpful in the DCD with A-NRP retrieval specially at the very beginning of the process till the start of the antegrade cardio and pneumoplegia. This is not only helpful but also comfortable for the surgeons and the scrub to work in harmony and to prevent unwanted events as well as to maintain sterility in a hasty procedure.

Some teams would prefer a second trolley is prepared for the assistant and will be on the left of the donor

The trolley contains: SEE PHOTO

- Two suckers (Cell saver and wall sucker (Cell saver marked by a black tie to distinguish from wall sucker and not be used once cardioplegia started)
- Clamp for the SVC
- Two Dunhill clips.
- One Abdo pack
- Heparin syringe
- Two forceps
- Chest retractor
- Cardioplegia and pneumoplegia lines.

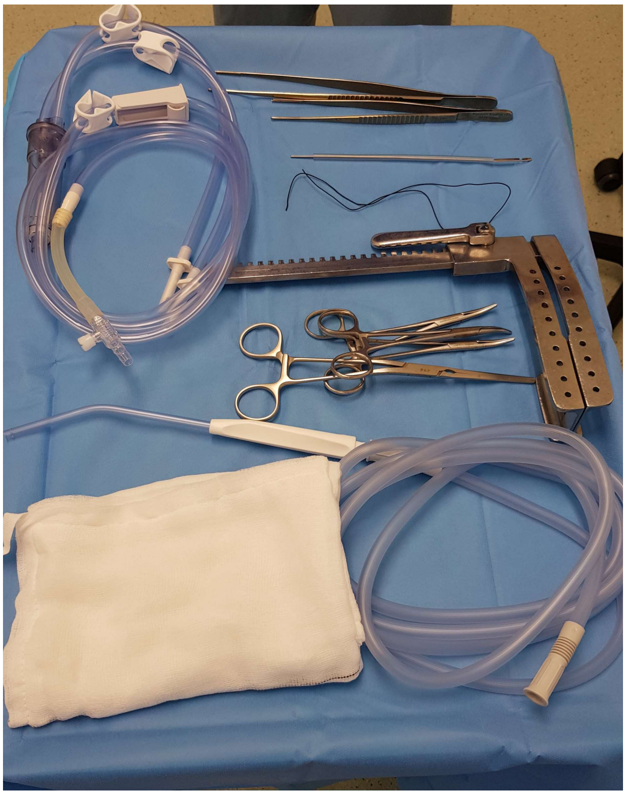

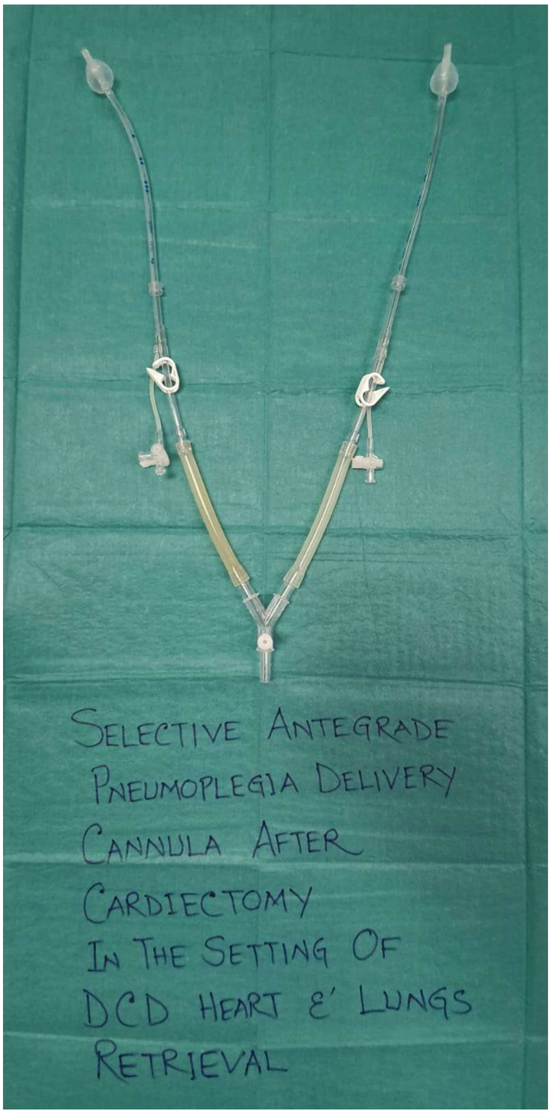

In the setting of DCD heart and lungs retrieval cardioplegia delivery finishes before pneumoplegia. But, if one has to wait for the pneumoplegia to finish before starting procurement of the heart, several precious minutes will be lost. To avoid that as soon as cardioplegia delivery finishes, antegrade pneumoplegia delivery can be paused for procurement of the heart. After that through the cut end of main pulmonary artery the selective antegrade pneumoplegia delivery cannula (shown in the figure) can be used to complete the rest of the pneumoplegia.

Cardiothoracic Synchrony between the Surgeons

**SURGEON 1**	**SURGEON 2**
• Skin Incision<br>• Sternotomy	Handle the suckers and the plegia lines
	• Placing the Sternal retractor (Not fully opened in order not to stretch the pericardium
• Opening of the pericardium<br>• Opening of the Left pleura<br>• Retracting the Left lung to expose the descending thoracic aorta	
• Inject heparin in right atrium	Inject heparin in PA
	• Clamping the Descending Thoracic Aorta with a long clamp
• Incising right atrial appendage and collection blood for OCS	
• Ascending Aortic clamp<br>• Insertion of venting needle distal to the clamp	
	• Securing the venting needle/cannula
~~• Rule out CAD~~	
• Venting the Right (Clamping the IVC in the pericardium and Flush cutting) and Left Heart (through LAA or LSPV)<br>• Inserting with wide bore cannula (medicut) **and holding it in place** proximal to the ascending aortic clamp to deliver antegrade cardioplegia	• SVC clamp caudal to Azygos away from SA node
	• Connecting the cardioplegia line to the cannula

	• Surface cooling with cold saline
• At the completion of the cardioplegia, careful procurement of the heart (after securing the Azygos and ensuring adequate SVC length)	• Helping the Surgeon 1
• Heart out and preparing it for OCS in the back table	
	• Securing bleeding points and ensuring haemostasis for a smooth A-NRP run

National Organ Retrieval Service

Perioperative Competence Programme

Abdominal Organ Preservation

Normothermic Regional Perfusion Competencies

Authors: Fiona Hunt, Gabriel Oniscu, Edinburgh Transplant Centre ©. Version 4: September, 2021. Review September 2022.

Aims and learning outcomes of programme

The aims of the programme are two fold:

1. To equip the practitioner with the necessary skills and knowledge base to practice in a competent manner as part of the National Organ Retrieval Service (NORS).
2. To provide a standard of practice against which existing practitioners in NORS can measure and develop their levels of competence.

The learning outcomes of the framework have been devised to enable the practitioner to:

1. Demonstrate best practice throughout the organ retrieval procedure
2. Demonstrate competence in a range of skills relevant to their role with the NORS
3. Demonstrate competence in the application of appropriate knowledge and understanding pertaining to the work of the NORS team and their role within the team.

Programme outline

This programme utilises a mixture of learning and teaching approaches to enable the practitioner to complete the competencies. The main emphasis is on self-directed learning as this recognises the different levels of experience, abilities and motivation of the practitioners undertaking the programme (O'Shea 2003).

The practitioner will be assigned a clinical mentor who will provide support and facilitate learning opportunities until such time as the practitioner has successfully completed the programme. Existing practitioners can request the appointment of a mentor who is willing to take on the role and has, ideally, completed an accredited mentorship course.

The formative assessments can be signed off by a suitably experienced registered practitioner in organ retrieval and final summative assessment and declaration of competence will be undertaken by the lead perioperative practitioner within your NORS team.

The practitioner's achievement of the competency framework will be assessed through a portfolio of evidence. Portfolio-based learning has become a standard method of assessing competence-based education as it enables a valid and true assessment of the complex repertoire of knowledge and skills required in the modern healthcare system (Redfern *et al* (2002).

Five constituents for the acceptability of evidence are outlined in the *Portfolio of Core Competencies for Anaesthetic Assistants* (NHS Education for Scotland 2007). These are as follows:

1. Validity: Does the evidence meet the needs of the indicator it is being used towards?
2. Authenticity: Can the evidence be attributed to the practitioner?
3. Sufficiency: Is there enough evidence to infer achievement?
4. Currency: Is the evidence up to date and relevant?
5. Reliability: Does the evidence accurately reflect the skills and knowledge required?

Types of admissible evidence for this programme include: quantifiable evidence i.e. skills check lists, e-learning and private study i.e. journals/books, reflective pieces, internal courses and, peer teaching.

Formative assessment can be undertaken by any competent practitioner but final, summative assessment must be undertaken by the Senior Perioperative Practitioner for the NORS.

Practitioners will also be expected to maintain a record of the organ retrievals attended during their training programme and beyond. The logbook should record the dates, locations, and types of retrievals attended. Additional comments could include activities undertaken during the retrieval as well as positive and negative reflections on the experience. The objective of the logbook is to offer evidence of the practitioner's experiences and development throughout the training programme and evidence of competence maintenance.

References

NHS Education for Scotland (2007) *Portfolio of Core Competencies for Anaesthetic Assistants.* NES: Edinburgh

O'Shea, E. (2003) Self-directed learning in nurse education: a review of the literature. *Journal of Advanced Nursing* 43 (1) pp. 62-70

Redfern, S. Norman, I. Calman, L. Watson, R. Murrells, T. (2002) Assessing competence too practice in nursing: a review of the literature. *Research Papers in Education* 17 (1) pp. 51-77

Competency: **Normothermic Regional Perfusion (NRP)**

Supervised practice and final competence assessment MUST only be undertaken following successful completion of theoretical component.

NHS Lothian Normothermic Regional Perfusion (NRP)

Theoretical component:

Training session with Maquet Getinge Group

- Configuration and data setup for Cardiohelp
- Normal use
- Trouble-shooting

Training Sessions with Registered Practitioner competent in Normothermic Regional Preservation (NRP)

- Fluency in all steps of the Surgical protocol
- Correct selection and preparation of equipment
- Assembly and priming of circuit
- Establishing NRP
- Discontinuation of NRP and conversion to Cold Preservation
- NRP troubleshooting
- Clinical and biochemical organ function monitoring
- Disassembly and cleaning
- Data monitoring and collection

**Competence**	Assessor Initials (formative)	Assessor Initials (formative)	Assessor Initials (formative)	Assessor Initials (formative)	Assessor Initials (summative)
Can discuss rationale behind NRP and can identify the potential benefits of NRP.					
Demonstrates a good working knowledge/understanding of the DCD/NRP National Protocol.					
Demonstrates an understanding of the constitution of an Organ Retrieval team for NRP in accordance with National Protocol..					
Demonstrates ability to identify and select equipment for DCD/NRP retrieval.					
Can discuss the importance of good communication and building a rapport between the multidisciplinary team and other members of the NORS teams.					
Demonstrates ability to liaise appropriately with staff from the donor hospital and identify additional equipment required.					
Can clearly demonstrate knowledge of what is required to prepare theatres for DCD/NRP retrieval.					
Can demonstrate a good working knowledge of specific NRP equipment and consumables.					
Can clearly identify the fluids/drugs prescribed for the composition of the circuit priming fluid.					
Can prepare priming solution in accordance with National protocol and Health Board or Trust IV additive policy.					
Demonstrates knowledge and correct selection of the appropriate cannulae required to cannulate for abdominal or femoral cannulation.					

**Competence**	Assessor Initials (formative)	Assessor Initials (formative)	Assessor Initials (formative)	Assessor Initials (formative)	Assessor Initials (summative)
NRP system set-up: *Demonstrates the ability to assemble and prepare the NRP console and pump in line with manufacturer's instructions in preparation for use.*					
Assembly of Circuit: *Demonstrates the ability to assemble the circuit in line with manufacturer's instructions accounting for the requirements to maintain sterility within the surgical field.*					
Priming & De-airing: *Can clearly demonstrate the ability to correctly prime the circuit in accordance with national protocol whilst adhering to manufacturer's guidelines.*					
Establishing NRP: *Can demonstrate ability to establish NRP circuit when instructed by lead surgeon – increasing flow rate, releasing venous clamp, ensuring adequate venous drainage, releasing arterial clamp & increasing blood flows within agreed parameters.*					
During NRP: *Demonstrates ability to control and maintain NRP circuit within the parameters set out in the National Protocol, can show awareness of circuit problems(clots, bubbles, loss of volume) and discuss and perform NRP troubleshooting measures correctly.*					
Demonstrates the ability to perform biochemical, haematological & blood gas analysis as per protocol.					
Demonstrates an awareness of clinical and biochemical evaluation of the abdominal organs.					
Can show clear understanding and discuss the haemodynamic and biochemical goals suggested in the National Protocol and can provide the rationale behind giving certain prescribed additives to perfusion fluid.					
Post NRP*: Demonstrates the ability to cease NRP and convert to cold perfusion as per surgical protocol in both planned and emergency scenarios.*					

**Competence**	Assessor Initials (formative)	Assessor Initials (formative)	Assessor Initials (formative	Assessor Initials (formative	Assessor Initials (summative)
Demonstrates understanding of the necessity of accurate documentation in NRP prescription chart and dissemination NHSBT NRP passport.					
Demonstrates knowledge of the additional blood and tissue samples to be collected.					
Demonstrates an understanding of the importance of equipment cleaning and maintenance according to Manufacturers Protocol and Local Health Board /Trust Policy.					
Discuss the importance for re-stocking consumables on return from retrieval.					

edinburgh transplant centre

National Organ Retrieval Service

Certificate of Competence

Has successfully completed:

Abdominal Organ Preservation

Normothermic Regional Perfusion Competencies

Candidate
NAME: ______________________________________
SIGNATURE: __________________________________
DATE: _____________________

Lead Perioperative Practitioner
NAME: ______________________________________
SIGNATURE: __________________________________
DATE: _______________________

Authors: Fiona Hunt, Gabriel Oniscu, Edinburgh Transplant Centre ©. Version 4: September, 2021. Review September 2022.

Abbott iStat Alinity operation

Purpose and Scope of Procedure

This document outlines the procedures to follow for blood gas measurement on the Abbott iStat Alinity.

**Safety Critical Level Statement**	
**This SOP contains safety critical steps within**	
**This SOP does not contain safety critical steps within**	**X**

Term	Definition

This is a controlled document: This copy is valid on day of print only, after which the user must ensure that this is the correct version by comparing against the current document details in Q-Pulse

Contents

Hazards and Precautions....3
Risk Assessment....3
Identification of key hazards....3
Tools, Reagents and Equipment....3
Pre Start Conditions (including information on patient preparation, sample types and containers, if applicable)....4
Procedural steps (including Quality Control steps if applicable)....4
Appendix....6
General and COSHH Risk Assessments Information....6
Principle and method....7
Performance Characteristics....7
Calibration (including metrological)....7
Quality Control procedures....7
Interferences....7
Principle of result calculation....7
Biological Reference Intervals....7
Reportable interval....8
Instructions for determining quantitative results....8
Alert/Critical Value....8
Laboratory Clinical Interpretation....8
Sources of Variation....8
References and related documents....8

This is a controlled document: This copy is valid on day of print only, after which the user must ensure that this is the correct version by comparing against the current document details in Q-Pulse

Hazards and Precautions

Risk Assessment

Care should be taken to ensure any used sharps are handled and disposed of in line with NHS Lothian policy. All used cartridges should be disposed of in a sharps bin/ clinical waste bin.
Any waste produced from the analysis of a blood sample should be disposed of safely in a clinical waste bin.
Hands should be washed immediately with soap and water and thoroughly dried using paper towels after all waste has been disposed of and testing has ended.

Cleaning the analyser: clean the display and case with a gauze pad moistened with a non-abrasive cleaner, detergent, soap and water or alcohol or. Rinse with another moistened pad with water and dry.

Current Overall level of Risk [insert overall risk matrix level and colour box here]

Yellow

Identification of key hazards

Hazard	Risk	Precaution
Biological	Exposure to infectious biological material	PPE

Tools, Reagents and Equipment

Abbott iStat Alinity analyser

Consumables	Part No.	Storage Temp°C
G4+ Cartridge	O3P85-25	2 - 8
Chem8+ Cartridge	09P31-25	2 - 8
Control level 1	05P71-01	2 - 8
Control level 3	05P73-01	2 – 8

This is a controlled document: This copy is valid on day of print only, after which the user must ensure that this is the correct version by comparing against the current document details in Q-Pulse

Pre Start Conditions (including information on patient preparation, sample types and containers, if applicable)

Withdraw blood from sampling line into a plain plastic sterile syringe.

Procedural steps (including Quality Control steps if applicable)

Performing a patient test

Testing personnel MUST be properly trained by the Abbott company representative. Training records should be filed and maintained on site

Take the sample to the i-STAT

1. Turn the i-STAT on using the bottom right hand button.
2. Press 2 to select i-STAT cartridge.
3. Enter operator ID by scanning the barcode present on your ID badge, by pressing and holding the scan button. Analyser will bleep when barcode has been read. If you do not have a barcode on your badge, you can type in your name instead
4. Enter the patients ID by manually entering the ODT number
5. Enter the cartridge lot number by scanning the barcode on the cartridge packaging (located at the bottom of the individual packet).
6. The insert cartridge screen should be visible. This will remain present for 15 minutes.
7. Place the analyser on a flat surface, ready for testing.
8. Carefully remove the cartridge from the packaging, holding it at the sides only, ensuring that no pressure is exerted over the centre of the cartridge and that nothing comes into contact with the electrodes.
9. Place the cartridge next to the analyser in a convenient location.
10. Wash hands and wear apron and gloves in line with infection control procedure.
11. Obtain a sample in a plain syringe
12. Immediately transfer the sample into the cartridge well, slowly filling until the fill mark is reached.
NB it is possible to under and overfill the cartridge so ensure volume is correct before entering the cartridge (sample volume 95µl).
13. Carefully fold the snap closure over the sample using the far edge, ensuring pressure isn't exerted directly over the well.
14. Holding the cartridge by its edges, introduce it in to the bottom of the analyser until it clicks into place.
15. **Do not attempt to remove the cartridge while the cartridge locked message is displayed at the bottom of the screen, this will result in expensive damage.**
16. If required, use the left and right arrow keys to scroll through results and enter extra information. The amount of oxygen the patient is using should be entered as a percentage (e.g 21 - room air, 28 - 28%) for those on a venturi enter as litres (eg 2, 4, etc) for those on nasal cannulae, etc.
17. Results will be displayed in approx 2 minutes.
18. Once results have been displayed and cartridge locked message disappears, remove cartridge and dispose into a sharpsafe container.
19. Use the left and right arrow keys to scroll through results and enter extra information

This is a controlled document: This copy is valid on day of print only, after which the user must ensure that this is the correct version by comparing against the current document details in Q-Pulse

20. Make a note of the results in the blood gas record table ensuring all fields are completed. Results must also be recorded in the patient notes.
21. Remove apron and gloves and wash hands
22. To print the results, switch the printer on, align the infra red windows on the printer and analyser and press and hold the print button until complete.
23. The blood gas results are documented onto the NRP paperwork. This is stored with the retrieval paperwork as per protocol.

Quality Control

Internal simulator
The internal simulator test will be performed every time a cartridge is inserted prior to sample analysis. PASS results will not be displayed on the analyser screen but will appear on analyser stored results.

External Simulator
This should be performed every **24hours** and results recorded in the QC log. The external simulator also needs to be performed when:
1. The internal simulator test fails
2. When a new lot of cartridges are received
3. If the instrument is dropped

How to run external simulator:
1. Turn the analyser on
2. Press the menu key to access the Administrative Menu
3. Press the 3 key for Quality Tests
4. Press the 4 key for Simulator
5. Scan/enter Operator ID
6. Scan/enter the Simulator ID (serial No)
7. Insert the simulator in to the cartridge port
8. View results on the screen
9. If **PASS** is displayed, continue to use the analyser
10. If **FAIL** is displayed, re-insert the simulator and run the check again
11. If **FAIL** is displayed a second time **DO NOT** use the analyser and contact the **POINT OF CARE TEAM –** labspoctteam@nhslothian.scot.nhs.uk

This is a controlled document: This copy is valid on day of print only, after which the user must ensure that this is the correct version by comparing against the current document details in Q-Pulse

Both levels of QC must be performed monthly

1. Remove QC ampoule from the fridge and leave at room temperature for a minimum of 4 hours
2. Turn on the iStat
3. Press the menu key to access the Administrative menu
4. Press 3 for quality tests
5. Press 1 for control
6. Scan / enter operator ID
7. Scan / enter control lot number
8. Scan / enter cartridge lot number
9. Shake the ampoule for 5 – 10 seconds. Break the ampoule at neck. Immediately transfer the solution from ampoule to a caplllary tube or plain syringe and then immediately transfer solution to the cartridge. Seal the cartridge and insert into the cartridge port
10. View the results on the analyser display. Compare the results obtained with the target ranges on the assignment sheet. **Ensure the lot number on the sheet corresponds with the lot number of the QC used,** Record the results on the QC log sheet
11. Remove and discard the cartridge when the cartridge locked message disappears
12. Press 1 for test options and 1 for next level for testing another level of QC. Repeat steps 7 – 11.

Appendix

General and COSHH Risk Assessments Information

**Biological Risk**	**Precautions**	***Any pertinent other***

This is a controlled document: This copy is valid on day of print only, after which the user must ensure that this is the correct version by comparing against the current document details in Q-Pulse

		Reference material
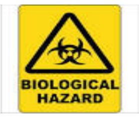	There is a risk of contact with blood borne viruses and other infective agents when handling patient samples. .	

Principle and method
See i-STAT user manual

Performance Characteristics

Calibration (including metrological)
CLEW software updates every 6 months

Quality Control procedures
(If not included above)

Interferences

Principle of result calculation

Biological Reference Intervals

pH	7.35 – 7.45
pCO2	4.7 – 6.4 KPa
pO2	11.1 – 14.4
Actual Bicarbonate	21 – 29 mmol/L

Document printed from Q-pulse 09/03/2022 12:23:00 by Gabriel Oniscu

This is a controlled document: This copy is valid on day of print only, after which the user must ensure that this is the correct version by comparing against the current document details in Q-Pulse

Reportable interval

Instructions for determining quantitative results

Alert/Critical Value

Laboratory Clinical Interpretation

Sources of Variation

References and related documents

NHS Lothian Policy on Point of Care Testing

Management and use of IVD point of Care Test Devices, Medical Device Agency MDA DB2010(02)

Guidelines on Point-of-Care Testing, The Royal College of Pathologists, 2004

Guidelines on near to patient or point of care testing. Joint Working Group on Quality Assurance 1999

This is a controlled document: This copy is valid on day of print only, after which the user must ensure that this is the correct version by comparing against the current document details in Q-Pulse

TRAINING PLAN FOR	i-STAT Alinity POCT Analyser

Name	

Level of Training	Training Start Date	Training Completed<br>The above named member of staff is considered competent in performing the above task/procedure to the level to which they have been trained			Review Date
		Date	Trainer signature	Trainee Signature	
**A:** The staff member will have a limited understanding of theory and practice principles that underpin the task/procedure. They will work under close but not continuous supervision.					
**B** The staff member will have a broad understanding of theory and practice principles that underpin the task/procedure. They will work under indirect supervision.					
**C:** The staff member will have theory and practice principles equivalent to that required of a registered practitioner. They will work under indirect supervision					
**D**: The staff member will have specialist understanding of theory and practice principles that underpin the task/procedure. They will work autonomously and have the underpinning knowledge to participate in out of hours.					
**E:** The staff member will have advanced knowledge in a specialist area and have specialist understanding of theory and practice principles that underpin the task/procedure. They will work autonomously and have the underpinning knowledge to participate in out of hours.					

This is a controlled document: This copy is valid on day of print only, after which the user must ensure that this is the correct version by comparing against the current document details in Q-Pulse

The following standard operating procedures/ reference documentation relate to this Task/Procedure

Standard operating procedure/ reference documentation	Q-Pulse Number
**i-STAT Alinity Procedure**	

I can confirm I have read and, where appropriate, have acknowledged these on Q-Pulse			
**Trainee**		**Date**	
**Trainer**		**Date**	

Knowledge Evaluation Questions / Case Studies / Exercises

LEVEL A

Demonstrate the following

- **All materials for sample collection brought to the test area**
- **Patient identified correctly**
- **Specimen collection prepared correctly**
- **Operator and patient ID scanned correctly**
- **Cartridge barcode scanned correctly**
- **Cartridge handled correctly, filled and inserted correctly**
- **Instrument remains on level, non vibrating surface during testing**
- **Cartridge removed and contaminated items disposed of correctly after completion of test**

OBJECTIVE EVIDENCE
Demonstrate performing a test under supervision attach printout

LEVEL B

- **Demonstrates understanding of cartridge storage requirements and cartridge expiration date policies**
- **Demonstrates understanding of Quality Control requirements, including verifying correct acceptable ranges, verifying results within acceptable ranges, and correctly responding to out of range results**

OBJECTIVE EVIDENCE

Print out evidence that you have run a QC on the analyser

LEVEL C

- **Instrument rechargeable battery recharged correctly**
- **Cleaning and decontamination of the instrument performed correctly with approved cleaning method**

OBJECTIVE EVIDENCE
Demonstrate maintenance procedure under supervision

LEVEL D

- **Policy and procedure for reporting critical results followed correctly**
- **Correctly demonstrates ability to recall stored results**

OBJECTIVE EVIDENCE
Demonstrate how to recall stored results

LEVEL E

- **Operator demonstrates understanding of action to be taken in response to Quality Check Failures**
- **Operator demonstrates understanding of correct response to instrument icons indicating low battery**

OBJECTIVE EVIDENCE
Demonstrate changing the QC ranges under supervision

This is a controlled document: This copy is valid on day of print only, after which the user must ensure that this is the correct version by comparing against the current document details in Q-Pulse

I can confirm that the above questions/ case studies/exercises have been answered satisfactorily to the level to which they have been trained.

These questions have been asked orally YES/NO

The model answers to these questions can be found:

**Trainee**		**Date**	
**Comment**			
**Trainer**		**Date**	
**Comment**			

This is a controlled document: This copy is valid on day of print only, after which the user must ensure that this is the correct version by comparing against the current document details in Q-Pulse

Training Log

Level	TASK/PROCEDURE	Training outcome						
		Observation of task		Performance under supervision		The above named member of staff is considered competent in performing the above task/procedure to the level to which they have been trained		
		Demonstrated by	Date	Supervised by	Date	Trainer signature	Trainee signature	Date
**A**	**Performing a patient sample**							
**B**	**Cleaning and quality control**							
**C**	**Maintenance procedure**							
**D**	**Recall results**							
**E**	**Troubleshooting**							

This is a controlled document: This copy is valid on day of print only, after which the user must ensure that this is the correct version by comparing against the current document details in Q-Pulse

Piccolo Xpress Chemistry Analyser Operation

The Piccolo Xpress chemistry analyser provides quantatative determinations of Alanine aminotransferase (ALT), Albumin, Alkaline phosphatase (ALP), Amylase, Aspartate aminotransferase (AST), C-reactive protein (CRP),Calcium, Creatinine,Gamma glutamyltransferase (GGT), glucose, total protein,urea and urric acid in lithium heparin whole blood or serum

Document printed from Q-pulse 09/03/2022 12:25:00 by Gabriel Oniscu

This is a controlled document: This copy is valid on day of print only, after which the user must ensure that this is the correct version by comparing against the current document details in Q-Pulse

1. TABLE OF CONTENTS

1. TABLE OF CONTENTS 2
2. HAZARDS AND PRECAUTIONS. 3
2.1. Procedure Risk Assessment 3
2.2. Chemical 3
2.3. Biological Hazard 4
2.4. Physical Hazard 4
2.5. Room Risk Assessment (if required) 4
3. CONTENT 5
A) PURPOSE OF THE EXAMINATION 5
B) PRINCIPLE AND METHOD OF THE PROCEDURE USED FOR EXAMINATIONS 5
C) PERFORMANCE CHARACTERISTICS (SEE 5.5.1.2 AND 5.5.1.3) 9
D) TYPE OF SAMPLE (E.G. PLASMA, SERUM, URINE) 9
E) PATIENT PREPARATION 9
F) TYPE OF CONTAINER AND ADDITIVES 9
G) REQUIRED EQUIPMENT AND REAGENTS 9
H) ENVIRONMENTAL AND SAFETY CONTROLS 9
I) CALIBRATION PROCEDURES (METROLOGICAL TRACEABILITY) 9
J) PROCEDURAL STEPS 10
K) QUALITY CONTROL PROCEDURES 12
L) INTERFERENCES (E.G. LIPAEMIA, HAEMOLYSIS, BILIRUBINEMIA, DRUGS) AND CROSS REACTIONS 15
M) PRINCIPLE OF PROCEDURE FOR CALCULATING RESULTS INCLUDING, WHERE RELEVANT, THE MEASUREMENT UNCERTAINTY OF MEASURED QUANTITY VALUES 15
N) BIOLOGICAL REFERENCE INTERVALS OR CLINICAL DECISION VALUES 15
O) REPORTABLE INTERVAL OF EXAMINATION RESULTS 15
P) INSTRUCTIONS FOR DETERMINING QUANTITATIVE RESULTS WHEN A RESULT IS NOT WITHIN THE MEASUREMENT INTERVAL 16
Q) ALERT/CRITICAL VALUES, WHERE APPROPRIATE 16
R) LABORATORY CLINICAL INTERPRETATION 16
S) POTENTIAL SOURCES OF VARIATION 16
T) REFERENCES 16

Document printed from Q-pulse 09/03/2022 12:25:00 by Gabriel Oniscu

This is a controlled document: This copy is valid on day of print only, after which the user must ensure that this is the correct version by comparing against the current document details in Q-Pulse

2. HAZARDS AND PRECAUTIONS.

2.1. Procedure Risk Assessment

	**Hazard**	**Risk**	**Assessment of Risk**	**Overall Procedural Risk**
**Chemical**	*Minor injury*	*Possible*	*Medium*	*Medium*
**Biological**	*Death*	*Rare*	*Low*	
**Physical**	*Minor Injury*	*Unlikely*	*Low*	
**Room**	*Death*	*Rare*	*Low*	
**Mechanical**	*N/A*	*N/A*	*N/A*	

2.2. Chemical

Please note – only chemicals which present a risk when used in this procedure need to be included in this table.

**Chemical Risk**	**Precautions**	**Storage and Discard Requirements**	**1st Aid Measures**	**MSDS reference** (Q-pulse)
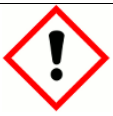 *Skin irritant lyophylised chemical beads enclosed in a plastic rotor*<br><br>Chemicals used in rotor:<br>D-Mannitol<br>Polyethylene glycol, 8000<br>Dextran, 70 USP<br>Tris(hydroxymethyl) amino<br>Polyethylene glycol, 3400<br>Polyethylene glycol, 2000<br>Sodium Chloride<br>POPSO, Disodium salt<br>Sodium Thiocyanate<br>L-Aspartic Acid<br>Lithium Hydroxide,<br>Tris, HCL	Wear gloves when handling rotor	Store in fridge and discard into a sharpsafe™	Fush exposed skin with copious amounts of water for at least 15 minutes	POCT-SDS4

This is a controlled document: This copy is valid on day of print only, after which the user must ensure that this is the correct version by comparing against the current document details in Q-Pulse

2.3. Biological Hazard

Biological Risk	Precautions	Precautions
There is a risk of contact with blood borne viruses and other infective agents when handling patient samples and quality control	Wear Gloves when handling samples and quality control	

2.4. Physical Hazard

Please note – this includes any manual handling and VDU risk assessments required and need to be included in this table.

Physical Risk	Precautions	1st Aid Measures	*Any pertinent other Reference material*
Electrical	Place the analyzer on a level surface that is free of hair, dust, and other contaminants. Do not place the analyzer near a sunny window or any other heat source.		

2.5. Room Risk Assessment (if required)

Please note – this includes any room which had special requirements for example dark rooms, CL3 laboratories.

Room Risk	Precautions	1st Aid Measures	*Any pertinent other Reference material*

Document printed from Q-pulse 09/03/2022 12:25:00 by Gabriel Oniscu

This is a controlled document: This copy is valid on day of print only, after which the user must ensure that this is the correct version by comparing against the current document details in Q-Pulse

3. CONTENT

A) PURPOSE OF THE EXAMINATION

The Piccolo Xpress chemistry system consists of a portable analyzer and disposable single-use reagent discs. Each reagent disc contains all the reagents needed to perform a panel of tests on a single sample. The Scottish Organ Retrieval Team transports the analyser to the donor hospital and the results are used to detemine the suitability of organs for transplant by analysing blood samples every 30 minutes during Normothermic Regional Perfusion.

B) PRINCIPLE AND METHOD OF THE PROCEDURE USED FOR EXAMINATIONS

The operator introduces a heparinized whole blood sample (or heparinized plasma, serum, or control) into the reagent disc. The reagent disc contains a diluent and test-specific reagent beads.
The operator then places the disc in the Piccolo Xpress chemistry analyser and enters the appropriate identification numbers.
The analyzer automatically performs the remainder of the testing protocol. The reagent disc spins and whole blood is separated into plasma and blood cells. During this time, the disc is heated to 37 ºC (98.6 ºF). Precisely measured quantities of plasma and diluent enter the mixing chamber and are mixed together. Through centrifugal and capillary forces, the diluted plasma is distributed to cuvettes on the perimeter of the disc. Reagent beads in the cuvettes are dissolved by the diluted plasma. This solution is thoroughly mixed and the resulting chemical reactions are monitored photometrically by the analyser. Optical signals generated by the chemical reactions are used to calculate analyte concentrations. Calibration data specific for the chemistries in each disc are provided to the analyzer by the bar code printed on the barcode ring

1. Alanine Aminotransferase (ALT)
   ALT catalyses the transfer of an amino group from L-alanine to α-ketoglutarate to form L-glutamate and pyruvate. Lactate dehydrogenase catalyses the conversion of pyruvate to lactate. Concomitantly NADH is oxidised to NAD+

   L-Alanine + α-ketoglutarate $\longrightarrow$ L-Glutamate + Pyruvate

   Pyruvate + NADH + H+ $\longrightarrow$ Lactate + NAD+
   The rate of change of the absorbance difference between 340nm and 405nm is due to the conversion of NADH to NAD+ and is directly proportional to the amount of ALT present in the sample

2. Albumin
   Bromocresol purple (BCP) when bound with albumin, changes colour from a yellow to blue colour. The absorbance maximum changes with the colour shift.

   BCP + Albumin $\longrightarrow$ BCP-Albumin Complex

This is a controlled document: This copy is valid on day of print only, after which the user must ensure that this is the correct version by comparing against the current document details in Q-Pulse

Bound albumin is proportional to the concentration of albumin in the sample. This is an endpoint reaction that is measured as absorbance at 600nm.

3. Alkaline Phosphatase (ALP)
   Alkaline phosphatase hydrolyses *p*-Nitrophenyl phosphate in a metal – ion buffer and forms *p*-nitrophenol and phosphate

   p-Nitrophenyl Phosphate → *p*-Nitrophenol + phosphate
   The amount of ALP in the sample is proportional to the rate of increase in absorbance between 405nm and 500nm

4. Amylase (AMY)
   The substrate, 2-chloro-*p*-nitrophenly-α-D-maltotrioside (CNPG3), reacts with α-amylase in the patient sample, releasing 2-chloro-*p*-nitrophenol (CNP). The release of CNP creates a change in colour.

   CNPG3 → CNP + D- Maltotrioside

   The reaction is measured bichromatically at 405nm and 500nm. The change in absorbance due to the formation of CNP is directly proportional to α-amylase activity in the sample

5. Aspartate Aminotransferase (AST)
   AST catalyses the reaction of L-aspartate and α-ketoglutarate into oxaloacetate and L-glutamate. Oxaloacetate is converted to malate and NADH is oxidised to NAD+ by the catalyst MDH

   L-aspartate + α-ketoglutarate → Oxaloacetate + L-glutamate

   Oxaloacetate + NADH + H+ → Malate + NAD+

   The rate of absorbance change at 340nm / 405mn caused by the conversion of NADH to NAD+ is directly proportional to the amount of AST present in the sample

6. Calcium (Ca)
   Calcium in the patient sample binds with arsenaso 111 to form a calcium dye complex

   Ca + arsenazo 111 → Ca arsenazo 111 complex

   The endpoint reaction is monitored at 405nm, 467nm and 600nm. The amount of calcium in the sample is proportional to the absorbence

7. Creatinine (CRE)
   Creatinine amidohydrolase hydrolyses creatinine to creatine. A second enzyme, creatine amidinohydrolsae, catalyses the formation of sarcosine from creatine. Sarcosine oxidase causes the oxidation of sarcosine to glycine, formaldehyde and hydrogen peroxide (H2O2). Peroxidase catalyses the reaction among hydrogen peroxide, 2,4,6-tribromo-3-hydroxybenzoic acid (TBHBA) and 4-aminoantipyrine (4-AAP) into a red quinoneimine dye. Sodium ferrocyanide and ascorbate oxidase are added to the reaction mixture to minimise the potential interference of bilirubin and ascorbic acid, respectively.

   Creatinine + H2O → Creatine
   Creatine + H2O → Sarcosine + Urea

This is a controlled document: This copy is valid on day of print only, after which the user must ensure that this is the correct version by comparing against the current document details in Q-Pulse

Sarcosine + H_2O + O_2 → Glycine + Formaldehyde + H_2O_2

H_2O_2 + TBHBA + 4-AAP → Red Quinoneimine dye + H_2O

Two cuvettes are used to determine the concentration of creatinine in the sample. Endogenous creatine is measured in the blank cuvette which is subtracted from the combined endogenous creatine and the creatine formed from the enzyme reactions in the test cuvette. Once the endogenous creatine is eliminated from the calculations, the concentration of creatinine is proportional to the intensity of the red colour produces. The endpoint reaction is measured as the difference in absorbance between 550nm and 600 nm.

8. Gamma Glutamyltransferase (GGT)

The addition of sample containing GGT to the substrates L-ɣ-glutamyl-3-carboxy-4-nitroaniline and glycylglycine (gly-gly) causes the formation of L-ɣ-glutamyl-glycylglicine (glu-gly-gly) and 3-carboxy-4-nitroaniline

L-ɣ-glutamyl-3-carboxy-4-nitroanilide + gly-gly → glu-gly-gly + 3-carboxy-4-nitroaniline

The absorbance of this rate reaction is measured at 405nm. The production of 3-carboxy-4-nitroaniline is directly proportional to the GGT activity in the sample.

9. Glucose (GLU)

The reaction of glucose with adenosine triphosphate (ATP), catalysed br hexokinase (HK) produces glucose-6-phosphate (G-6-P) and adenosine diphosphate (ADP). Glucose-6-phosphate dehydrogenase (G-6-PDH) catalyses the reaction of 6-G-P into 6-phosphogluconate and the reduction of nicotinamide adenine dinucleotide (NAD+) to NADH.

Glucose + ATP → G-6-P + ADP

G-6-P + NAD+ → 6-phosphogluconate + NADH + H+

The absorbance is measured bichromatically at 340nm and 850nm. The production of NADH is directly proportional to the amount of glucose present in the sample.

10. Total Bilirubin (TBIL)

Bilirubin is oxidised by bilirubin oxidase into biliverdin

Bilirubin + O_2 → Biliverdin + H_2O

Bilirubin is quantitated as the difference in absorbance between 467nm and 550nm. The initial absorbance of this endpoint reaction is determined from the bilirubin blank cuvette and the final absorbance is obtained from the bilirubin test cuvette. The amount of bilirubin in the sample is proportional to the difference between the initial and final absorbance measurement.

11. Total protein (TP)

The protein solution is treated with cupric (Cu11) ions in a strong alkaline medium. Sodium potassium tartrate and potassium iodide are added to prevent the precipitation of copper hydroxide and the auto- reduction of copper

This is a controlled document: This copy is valid on day of print only, after which the user must ensure that this is the correct version by comparing against the current document details in Q-Pulse

respectively. The Cu11 ions react with peptide bonds between the carbonyl oxygen and amide nitrogen atoms to form a coloured Cu-protein complex

Total protein + Cu11 → Cu-protein complex

The amount of total protein in the sample is directly proportional to the absorbance of the Cu-protein complex. The total protein test is an endpoint reaction and the absorbance is measured as the difference in absorbance between 550nm and 850nm.

12. Blood Urea Nitrogen (BUN)

In the coupled-enzyme reaction, urease hydrolyses urea into ammonia and carbon dioxide. Upon combining ammonia with α-ketoglutarate and reduced nicotinamide adenine dinucleotide (NADH), the enzyme glutamate dehydrogenase (GLDH) oxidises NADH to NAD+

Urea + H20 → 2NH3 + CO2

NH3 + α-ketoglutarate + NADH → L-Glutamate + H20 + NAD+

The rate of change of the absorbance difference between 340nm and 405nm is caused by the conversion of NADH to NAD+ and is directly proportional to the amount of urea present in the sample.

13. Uric Acid (UA)

Uricase catalyses the oxidation of uric acid to allantoin and hydrogen peroxide. Peroxidase catalyses the reaction among the hydrogen peroxide (H2O2), 4-aminoantipyrine (4-AAP) and 3,5-dichloro-2-hydroxybenzenesulphonic acid (DHBSA) into a red quinoneimine dye. Sodium ferrocyanide and ascorbate oxidase are added to the reaction to minimise the potential interference of bilirubin and ascorbic acid.

Uric acid + O2 + H2O → Allantoin + CO2 + H2O2

H2O2 + 4-AAP + DHBSA → Quinoneimine dye + H2O

This is a controlled document: This copy is valid on day of print only, after which the user must ensure that this is the correct version by comparing against the current document details in Q-Pulse

C) PERFORMANCE CHARACTERISTICS (SEE 5.5.1.2 AND 5.5.1.3)

C) TYPE OF SAMPLE (E.G. PLASMA, SERUM, URINE)

The Piccolo Xpress chemistry analyzer accepts **lithium-heparinised** whole blood, plasma, or serum samples

E) PATIENT PREPARATION

F) TYPE OF CONTAINER AND ADDITIVES

Withdraw blood from sampling line into a plain plastic sterile syringe. Decant into a sterile container and follow the steps for running a sample below.

.

G) REQUIRED EQUIPMENT AND REAGENTS

Operational Materials Catalog Number

Pipette tips, disposable, pack of 96 Abaxis #500-9007
Mini pipette, 100 ml, gray Abaxis #500-9006
Internal printer paper rolls, box of 6 Abaxis #1100-4410
Fan filter, covered Abaxis #1987-0009
Piccolo® Reagent Disc – General Chemistry 13
400-1029 (single); 400-0029 (10 pack); 400-0029-4 (4 pack)

H) ENVIRONMENTAL AND SAFETY CONTROLS

Refer to the various MSDS and kit inserts for instructions on the storage, handling and disposal of the material.
POCT-EXT6
POCT-SDS4
POCT-SDS5

I) CALIBRATION PROCEDURES (METROLOGICAL TRACEABILITY)

Refer to Piccolo traceability: ref POCT-EXT6

Document printed from Q-pulse 09/03/2022 12:25:00 by Gabriel Oniscu

This is a controlled document: This copy is valid on day of print only, after which the user must ensure that this is the correct version by comparing against the current document details in Q-Pulse

J) PROCEDURAL STEPS

Running a patient sample

Turn on the analyser by pressing the Power button on the front of the analyser
The analyser turns on then performs a self test
If the analyser needs time to warm the disc chamber to operating temperature the display shows ‘warming’

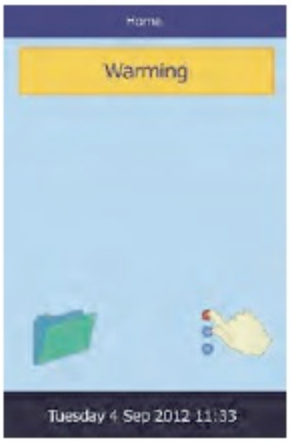

When the analyser reaches operating temperature it displays ‘analyse’

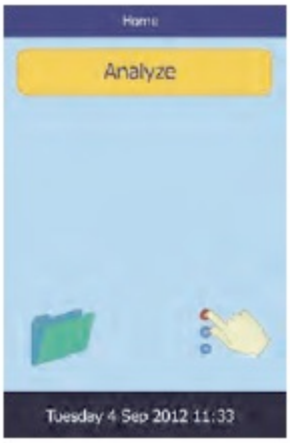

The reagent discs can be used straight from the fridge. The discs can remain in the sealed pouch at room temperature for a cummulative period of up to 48 hours

Check for tears and punctures in unopened foil pouch, use disk within 20 minutes of opening

Document printed from Q-pulse 09/03/2022 12:25:00 by Gabriel Oniscu

This is a controlled document: This copy is valid on day of print only, after which the user must ensure that this is the correct version by comparing against the current document details in Q-Pulse

Fill the sample chamber.

1. Using the Piccolo 100 µl volume pipette, firmly attach a new tip to the end of the pipette.
2. With your index finger or thumb, push the pipette button to the stop position and hold it down for sample pickup.
3. Immerse the tip 2–3 mm below the surface of the sample as shown below

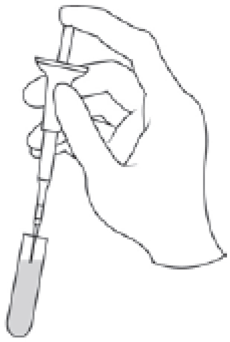

4. Slowly release the button to pick up the sample, pause then remove the pipette from the sample tube.
5. Make sure there are no air bubbles or air gaps in the pipette tip
6. Place the pipette tip into the discs sample chamber at 45° so that the entire sample flows into the sample chamber, the tip should touch the sample chamber

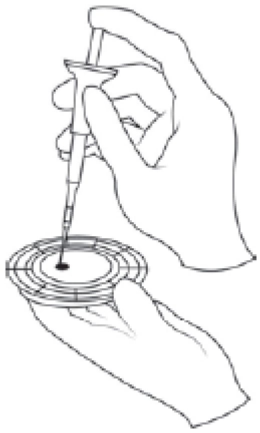

7. Push the plunger down with a slow, continuous motion. Take care not to overfill the sample chamber
8. Keep the the pipette plunger pressed down until the pipette tip is removed from the sample chamber
9. Discard the pipette tip into a sharpsafe™
10. Carry the prepared disc to the analyser. Hold the disc by it's edges and keep level to avoid spills
11. Place the disc in the recessed area in the drawer
12. Select **close**. The analyser closes the drawer

Document printed from Q-pulse 09/03/2022 12:25:00 by Gabriel Oniscu

This is a controlled document: This copy is valid on day of print only, after which the user must ensure that this is the correct version by comparing against the current document details in Q-Pulse

13. Select the sample type from patient or control
14. Enter an ID number for the sample
15. Then select done
16. The analyser checks the disc type and begins processing the sample
17. When the sample processing is complete the results will be printed automatically
18. Select **open** to open the disc drawer
19. Remove the disc from the drawer and discard into a sharpsafe™
20. When finished select **close** to close the drawer and return the analyser to standby mode

.

K) QUALITY CONTROL PROCEDURES

The control material used is Randox Abaxis chemistry controls level 1 and 2, which are supplied lyophilised.

To reconstitute:

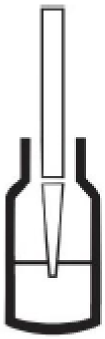

1. Carefully pipette 1ml diluent into the serum vial
   Push the pipette button to the first stop and hold down. Release slowly to pick up diluent. Push the pipette slowly to the second stop to dispense diluent into the serum vial.

This is a controlled document: This copy is valid on day of print only, after which the user must ensure that this is the correct version by comparing against the current document details in Q-Pulse

2. Close the serum vial and invert gently several times

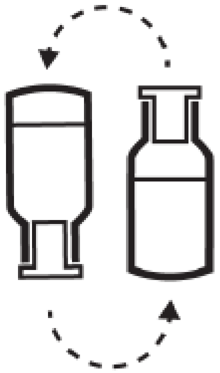

3. Allow to stand for 30 minutes before use. Ensure contents are completely dissolved by swirling gently. Avoid formation of foam. Do not shake.

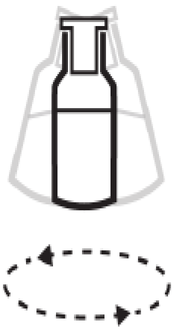

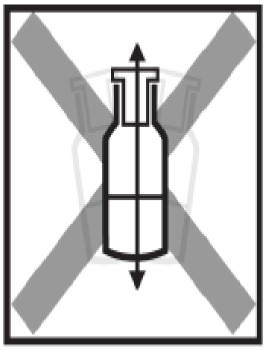

STORAGE AND STABILITY

OPENED: Store refrigerated (+2ºC to +8ºC). Reconstituted serum is stable for 8 hours at +15ºC to +25ºC or 7 days at +2ºC to +8ºC and 1 month when frozen once at -20ºC . Only the required amount of product should be removed. After use, any residual product should NOT BE RETURNED to the original vial.
UNOPENED: Store refrigerated (+2ºC to +8ºC). Stable to expiration date printed on individual vials.

Quality controls (level 1 and 2) are run before the instrument is used at the donor hospital

To run a QC

1. In the home screen, select 'analyse' to open the disc drawer

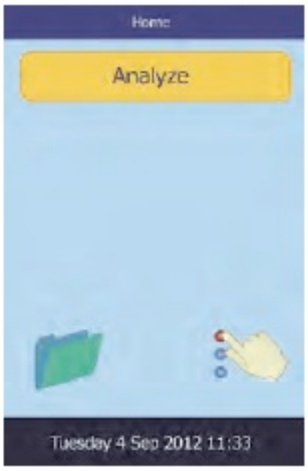

2. Select the control type to use by using the up and down arrow keys

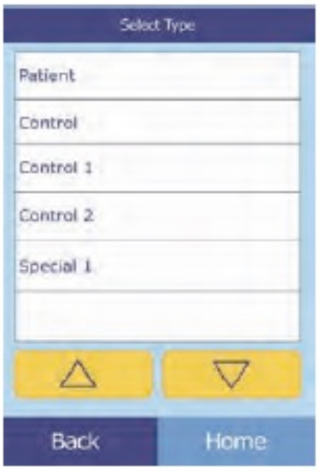

Using the reconstituted QC material analyse following steps 1 – 20 in running a patient sample

Once complete check the printout to make sure that the results are within the target range

This is a controlled document: This copy is valid on day of print only, after which the user must ensure that this is the correct version by comparing against the current document details in Q-Pulse

L) INTERFERENCES (E.G. LIPAEMIA, HAEMOLYSIS, BILIRUBINEMIA, DRUGS) AND CROSS REACTIONS

Any results that are affected by >10% interference from haemolysis, lipaemia or icterus are suppressed and HEM, LIP or ICT respectively are printed on the printout in place of the result.

For a complete list of interfering substances, please refer to the General Chemistry 13 package insert POCT-EXT6

M) PRINCIPLE OF PROCEDURE FOR CALCULATING RESULTS INCLUDING, WHERE RELEVANT, THE MEASUREMENT UNCERTAINTY OF MEASURED QUANTITY VALUES

Not applicable

N) BIOLOGICAL REFERENCE INTERVALS OR CLINICAL DECISION VALUES

Albumin	35 – 55 g/L
Alkaline Phosphatase	42 – 141 U/L
Alanine Aminotransferase	10 – 47 U/L
Aspartate Aminotransferase	11- 38 U/L
Amylase	14 – 97 U/L
Urea	2.5 – 7.9 mmol/L
Calcium	2.00 – 2.58 mmol/L
Creatinine	53 – 106 µmol/L
Gamma Glutamyltransferase	5 – 65 U/L
Glucose	4.1 – 6.6
Bilirubin	3 – 27 µmol/L
Total Protein	64 – 81 g/L
Uric Acid	2.2 – 8.0 mg/DL

O) REPORTABLE INTERVAL OF EXAMINATION RESULTS

Albumin	10 – 65 g/L
Alkaline Phosphatase	5 – 2400 U/L
Alanine Aminotransferase	5 – 2000 U/L
Aspartate Aminotransferase	5 – 2000 U/L
Amylase	5 – 4000 U/L
Urea	0.7 – 64.3 mmol/L
Calcium	1 – 4 mmol/L
Creatinine	18 – 1768 µmol/L
Gamma Glutamyltransferase	5 – 3000 U/L
Glucose	0.56 – 38.9 mmol/L
Bilirubin	1.7 – 513 µmol/L
Total Protein	20 – 140 g/L
Uric Acid	1-15 mg/dL

Document printed from Q-pulse 09/03/2022 12:25:00 by Gabriel Oniscu

This is a controlled document: This copy is valid on day of print only, after which the user must ensure that this is the correct version by comparing against the current document details in Q-Pulse

P) INSTRUCTIONS FOR DETERMINING QUANTITATIVE RESULTS WHEN A RESULT IS NOT WITHIN THE MEASUREMENT INTERVAL

When a result is outwith the measurement interval, a sample must be sent to the laboratory for confirmation

Q) ALERT/CRITICAL VALUES, WHERE APPROPRIATE

R) LABORATORY CLINICAL INTERPRETATION

Not applicable

S) POTENTIAL SOURCES OF VARIATION

Incorrect sample collection or collection into a tube with EDTA, fluoride oxalate or citrate as an anticoagulant will interfere with test results

T) REFERENCES

Piccolo Xpress User Operator's Manual POCT-EXT7
General Chemistry 13 Package insert POCT-EXT6
Piccolo Chemistry 13 SDS POCT-SDS4
Piccolo QC SDS POCT-SDS5

This is a controlled document: This copy is valid on day of print only, after which the user must ensure that this is the correct version by comparing against the current document details in Q-Pulse

TRAINING PLAN FOR	Piccolo Chemistry Analyser Training Plan

Name	

Level of Training	Training Start Date	Training Completed<br>The above named member of staff is considered competent in performing the above task/procedure to the level to which they have been trained			Review Date
		Date	Trainer signature	Trainee Signature	
**A:** The staff member will have a limited understanding of theory and practice principles that underpin the task/procedure. They will work under close but not continuous supervision.					
**B** The staff member will have a broad understanding of theory and practice principles that underpin the task/procedure. They will work under indirect supervision.					
**C:** The staff member will have theory and practice principles equivalent to that required of a registered practitioner. They will work under indirect supervision					
**D**: The staff member will have specialist understanding of theory and practice principles that underpin the task/procedure. They will work autonomously and have the underpinning knowledge to participate in out of hours.					
**E:** The staff member will have advanced knowledge in a specialist area and have specialist understanding of theory and practice principles that underpin the task/procedure. They will work autonomously and have the underpinning knowledge to participate in out of hours.					

This is a controlled document: This copy is valid on day of print only, after which the user must ensure that this is the correct version by comparing against the current document details in Q-Pulse

This is a controlled document: This copy is valid on day of print only, after which the user must ensure that this is the correct version by comparing against the current document details in Q-Pulse

The following standard operating procedures/ reference documentation relate to this Task/Procedure

Standard operating procedure/ reference documentation	Q-Pulse Number

I can confirm I have read and, where appropriate, have acknowledged these on Q-Pulse			
**Trainee**		**Date**	
**Trainer**		**Date**	

This is a controlled document: This copy is valid on day of print only, after which the user must ensure that this is the correct version by comparing against the current document details in Q-Pulse

Knowledge Evaluation Questions / Case Studies / Exercises

LEVEL A

1. **Where are the reagent disks stored?**
   Stored in fridge between 2 - 8°
2. **What is the correct operating temperature for the disks?**
   Can be used straight from the fridge, can remain in it's sealed pouch at room temperature for a cummulative period of 48 hours
3. **What checks should be made before using a disk?**
   Check for tears and punctures in unopened foil pouch, use disk within 20 minutes of opening

4. **What are the sample requirements?**
   A lithium Heparin whole blood sample

5. **Describe the sampling procedure**
   - **Attach tip to micropipette**
   - **push pipette button to stop position for sample pickup**
   - **immerse tip 2-3mm below the surface of the sample**
   - **slowly release button to pick up sample**
   - **place pipette tip into the disc sample chamber, tilt at 45° and push plunger down with a slow continous movement**
   - **discard the tip into a sharpsafe™**
   - **select patient on analyser**
   - **hold the disc by it's edges and keep level, insert disk into analyser**
   - **The results are printed after 12 minutes**
   - **Discard the used disk in a sharpsafe™**

OBJECTIVE EVIDENCE
Demonstrate performing a test under supervision

LEVEL B

1. **Describe how you would clean the instrument**
   Treat the instrument as potentially infected, contaminated with blood and bodily fluid spillages and follow the local policy for "decontamination of reusable non-invasive patient care equipment"
   See infection control manual:
   http://intranet.lothian.scot.nhs.uk/NHSLothian/Healthcare/A-Z/InfectionControl/icm/Documents/CP001.pdf

2. **How often is a QC run on the Piccolo?**
   Weekly and after moving instrument

3. **Where is the QC stored?**
   In fridge between 2 - 8°

4. **Describe the correct use of the pipette used to reconstitute the QC**
   - **Firmly attach tip**
   - **Depress pipette to first stop**
   - **Slowly release pipette to allow liquid to fill tip**
   - **Place tip at rim of vail to be reconstituted and slowly depress pipette to second stop**

5. **What are the steps to take following a QC fail result?**
   Repeat QC

OBJECTIVE EVIDENCE

Print out evidence that you have run a QC on the analyser

This is a controlled document: This copy is valid on day of print only, after which the user must ensure that this is the correct version by comparing against the current document details in Q-Pulse

LEVEL C

1. **What maintenance procedures are carried out on the analyser**
   Cleaning the filter twice per year

OBJECTIVE EVIDENCE
Demonstrate maintenance procedure under supervision

LEVEL D

1. **Can demonstrate correct packing and unpacking of the analyser**

OBJECTIVE EVIDENCE
Demonstrate correct packing / unpacking under supervision

LEVEL E

1. **Demonstrate how the quality control ranges can be changed**

OBJECTIVE EVIDENCE

I can confirm that the above questions/ case studies/exercises have been answered satisfactorily to the level to which they have been trained.

These questions have been asked orally YES/NO

The model answers to these questions can be found:

**Trainee**		**Date**	
**Comment**			
**Trainer**		**Date**	
**Comment**			

This is a controlled document: This copy is valid on day of print only, after which the user must ensure that this is the correct version by comparing against the current document details in Q-Pulse

Training Log

Level	TASK/PROCEDURE	Training outcome						
		Observation of task		Performance under supervision		The above named member of staff is considered competent in performing the above task/procedure to the level to which they have been trained		
		Demonstrated by	Date	Supervised by	Date	Trainer signature	Trainee signature	Date
A	**Performing a patient sample**							
B	**Cleaning and quality control**							
C	**Maintenance procedure**							
D	**Correct packing / unpacking**							
E	**Changing quality control ranges**							

This is a controlled document: This copy is valid on day of print only, after which the user must ensure that this is the correct version by comparing against the current document details in Q-Pulse

DCD CATIII with NRP

Equipment Checklist in addition to standard DCD

- NRP Machine
- NRP Circuit x 1
- Piccolo Xpress Analyser
- NRP bag (see bag checklist) – Femoral Cannulation Kit if req
- Yellow bin (for disposal of circuit)

NRP Bag Checklist

Green drug pouch

Pouch with memory stick, stapler & cable tie gun

Tubing clamps – 2 sets of 3

Syringes - 1ml x 12, 5ml x 2, 30ml x 2, 50ml Luer x 2

Universal container x 5

Sampling trays x 8

Green swabs

Eppendorf vials

FLUIDS

Hartmann's 1000ml x 2

Gelofusine 1000ml x 2

Gelofusine 500mls x 2

Bicarbonate 8.4% (250ml) x 2

ISTAT 1 & ISTAT 2

ISTAT printer + lead

Printer paper roll x 2

Electronic simulator

CG4+ cartridges x 15

CHEM 8+ cartridges x 15

Quick reference Guide

Extension Lead

NRP Sampling Box

NRP Folder

Quick Step Guides

Clipboard/Paperwork

-Green data and prescription charts

Inco. Pads

Floor mats

Fridge Items

Piccolo Cartridges x 10

Phentolamine 5 mgs

Pancuronium 12mgs

(3 amps)

Freezer

Level 1 Control

Level 2 Control

Surgical Extras Bag

- Coda balloon catheter
- Venous cannula – 36Fr &29Fr
- 50ml catheter tip syringe x 2
- 50ml luer lock syringe x 3
- 5ml syringe x 1
- Filter needle x 1
- Irrigation jet
- NaCl 0.9% (500ml) x 1
- Heparin 5000 units/5mls x 1
- White cap x 1
- Argyle snuggers x 2
- Aortic Root Cannula
- Cardiac Tourniquet x 1
- 30ml syringe x 1
- 3 way tap
- T tubes
- 3/0 prolene x 2

Femoral Cannulation Extras

- Venous cannula 25Fr/38cm length
- Arterial cannula 19Fr
- Vascular Dilator Kit
- Sterile Marker Pen
- Spare 3/8"x 3/8" and 3/8"x 1/2" connectors

Edinburgh Transplant Centre © October 2021. Review October 2022.

edinburgh transplant centre

NRP FLUID/DRUG CHECKLIST

Priming

- Hartmann's Solution (1000ml bag) x 2

Volume Replacement

- Gelofusine (1000ml bag) x 2
- Gelofusine (500ml bag) x 2

Boluses for Perfusate

- Bicarbonate 8.4%(250ml bottle-ml/kg required) x 2
- Heparin (5000 units/ml: 5ml vial-10ml required) x 4
- Teicoplanin 200mg vial x 1
- Gentamicin 40mg/ml x 3
- Metronidazole 500mg/100ml x 1
- Fluconazole (200 mg/100ml) x 2
- Methylprednisolone (1g box) x 1
- Pancuronium (4mg/2ml amp-12mgs required) x 3
- Phentolamine (10mg/ml-5mgs required) x 1

Hepsal

- NaCl 0.9% (500ml bag) x 1
- Heparin (5000units/5ml amp) x 1

Edinburgh Transplant Centre © October 2021. Review October 2022.

DCD CATEGORY III
NORMOTHERMIC REGIONAL PERFUSION

SNOD GUIDANCE TO BE READ IN ADDITION TO NATIONAL PROTOCOL

NB: DEFAULT POSITION FOR SURGICAL TEAM WILL BE TO REVERT TO STANDARD DCD CATEGORY III PROTOCOLS IF UNABLE TO ESTABLISH NRP

THE RETRIEVAL TEAM WILL BRING A REPLACEMENT QUOD BOX (IF AUTHORISATION) AND A SEPARATE NRP BOX SUPPORTED BY THE QUOD INFRASTRUCTURE

PRE THEATRE :

- MOBILISATION OF TEAM: Check with liver recipient co-ordinator if NRP to be used – if yes contact ODT hub and ask them to advise abdominal organ recipient centres that NRP is being used. If so, it may be necessary to add an extra 30 minutes prep/ theatre setup time (90 minutes in total). However, for well established NRP retrieval teams the usual 60 minute set up time is usually sufficient.

- LOCAL THEATRE TEAMS: Advise local teams of the potential longer set up time and also that the case itself will take 2 hours longer (more in keeping with DBD procedure). Ensure that the theatre team are aware of the theatre floor plan (ie. a large enough theatre) for DCD/NRP and the Donor Hospital Checklist, for additional equipment required by the retrieval team.

- RCC: Ensure that 4 units of RCC, cross-matched to the donor, will be available for theatre. These units of blood will be required to be released from the lab and stored within the theatre complex prior to the start of surgery. Ensure that the labs understand that the blood must be X-Matched to the patient (donor).

- LABS: Advise labs that bloods, LFTs X 2, (taken at 0hrs and 2hrs) will be sent together to the labs at the end of the 2 hour NRP procedure. They require to be tested as urgent samples. This will be to "back –up" biochemistry results from the accredited Piccolo.

THEATRE HANDOVER

- Agree with local team who will take samples at the end of NRP to the lab (a once only trip).

- Put lead surgeon in contact with thoracic surgeon to discuss NRP protocol if cardiothoracic organs being retrieved.

- Normal documentation handover and pre- theatre checks will be carried out. Handover QUOD box (if authorisation) as per protocol, ensuring completion /scanning of QUOD sheet.

DCD CATEGORY III
NORMOTHERMIC REGIONAL PERFUSION

- Discuss “timings” for organ stand down with lead surgeon and implanting centre– **NB.** This may be different to normal DCD protocol.

- Safety Brief

WITHDRAWAL

- Withdrawal and timings to theatre as normal

THEATRE

- Handover / checks as normal.
- Laparotomy, cannulation and connection to the NRP circuit.
- NRP will run for up to 2 hours.
- QUOD samples (if authorisation) taken and processed as normal with the exception of liver and kidney biopsies. These are placed in the NRP box with variances documented in the associated box paperwork. All other biopsies taken (spleen and left ureter) remain stored in QUOD box. This process is carried out by the retrieval team.
- In addition, NRP samples for service evaluation are taken from the NRP circuit at time 0hrs, 1hr and 2hrs (taken by retrieval team). These are placed in the NRP box as per national protocol (separate from QUOD box). NB. Specific authorisation is not required for these samples. Therefore, NRP samples are taken regardless of authorisation for QUOD.
- Other samples will be routinely taken for organ assessment purposes by the retrieval team.
- After 2hrs, the process will convert to cold perfusion when organ retrieval proceeds in a similar manner to DBD donation.
- Responsibility remains with surgeon to document biopsies on HOT A form and surgical team to complete QUOD worksheet.
- If authorisation, QUOD box and NRP box will be transferred back to RIE as before.

Theatre Floor Plan for DCD/NRP

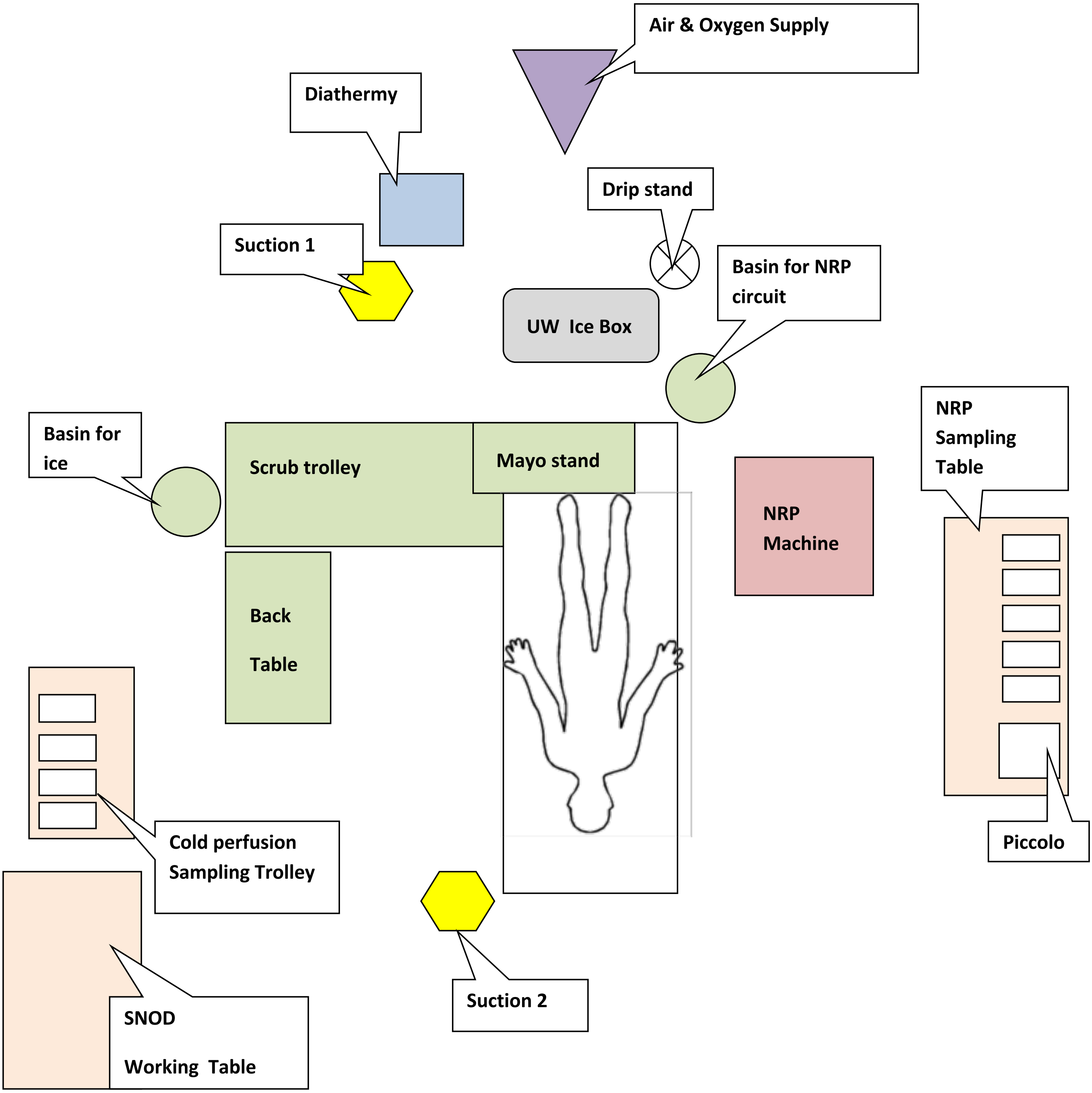

Donor Hospital Checklist

- **Blood tubes: U&Es/LFTs X 2**
- **Clinical chemistry forms X 2**
- **Blood culture bottles(aerobic & anaerobic) X 2 sets**
- **Bacteriology form X 2**

NORMOTHERMIC REGIONAL PERFUSION No. ______

DATE:	ODT No:	BLOOD GROUP:

ADDRESS LABEL

HOSPITAL:	
HEIGHT:	WEIGHT:
SNOD:	
SURGEON:	
APOPS:	

	TYPE	VOLUME DOSE	EXP DATE	BATCH NO	Prescribed by	Given by
PRIMING SOLUTION	Hartmann's Solution	1000mL				
	Hartmann's Solution	1000ml				
	Sodium Bicarbonate 8.4%	1mL / kg				
	Heparin Sodium 25,000 units / 5mL	50,000 units				
	Teicoplanin 200mg vial	400mg				
	Gentamicin 40mg/ml	120mg				
	Metronidazole 500mg/100ml	500mg				
	Fluconazole 2mg/mL	400mg				
	Methylprednisolone 1gram	1g				
	Pancuronium 4mg/2mL	12mg				
	Phentolamine 10mg/ml	5mg				
ADDITIONAL FLUIDS / DRUGS	TYPE	VOLUME DOSE	EXP DATE	BATCH NO	Prescribed by	Given by
	Gelofusine	500mL				
	Gelofusine	500mL				
	RCC	1 unit				
	RCC	1 unit				
	RCC	1 unit				
	RCC	1 unit				
	Maquet NRP Circuit	1				

NORMOTHERMIC REGIONAL PERFUSION No. ______

edinburgh transplant centre

Timings

**Withdrawal treatment:**	**Systolic BP <50mmHG:**
**Circulatory arrest:**	**Verification of death:**
**Knife to skin:**	**Start of NRP:**
**Cold in-situ flush:**	**End of procedure:**

Cannulation

**Venous cannula site:**	**Venous cannula size:**
**Arterial cannula site:**	**Arterial cannula size:**
	**Aortic arch cannula size:**

Pump Parameters

Time	Flow mls/min	Venous Sats %	HCT %	Pressures (mmHg)		Reservoir volume	Temp °C	Notes (additional fluids, drugs etc)
				Ven	Art			
0’								
10’								
20’								
30’								
40’								
50’								
60’								
70’								
80’								
90’								
100’								
110’								
120’								

NORMOTHERMIC REGIONAL PERFUSION No. ______

BLOOD RESULTS		RANGE	0	30’	60’	90’	120’
BLOOD GASES	pH	7.35-7.45					
	pCO2	4.70-6.40					
	pO2	11.1-14.4					
	HCO3	21.0-29.0					
	Sats	70-80%					
	BE	-2 - +3					
	Lact	0.4-1.4					
	Na+	136-145					
	K+	3.5-5.1					
	Gluc	3.6-5.2					
	Ca2+	1.15-1.29					
	Hct	35-40					
	Hb	115-165					
**BLOOD RESULTS**		**RANGE**	**0**	**30’**	**60’**	**90’**	**120’**
PICCOLO (GEN CHEM 13)	Gluc	5.2-7.2					
	Urea(BUN)	5.0-7.9					
	Crea	62-150					
	Uric Acid	274-363					
	Ca	1.90-2.40					
	ALB	24-40					
	Tot Prot	41-51					
	ALT	25-43					
	AST	26-41					
	ALP	64-107					
	Bili	15-29					
	GGT	28-44					
	AMY	47-87					
**BLOOD RESULTS**		**RANGE**	**0**	**30’**	**60’**	**90’**	**120’**
BIOCHEMISTRY	Bili	3-21					
	ALP	40-125					
	ALT	10-50					
	AST	10-40					
	Urea	2.5-6.6					
	Creat	50-98					
	Cultures						
**BILE RESULTS**		**RANGE**	**Point 0 @ __m**		**60’**	**120’**	
T Tube Size:	pH	7.35-7.45					
	Lact	0.4-1.4					
	Glu-	3.6-5.8					
	Volume						
	Vol total						

Edinburgh Transplant Centre ©.

NORMOTHERMIC REGIONAL PERFUSION No. ______

NOTES:

Donor Cause of Death:-

QUOD Box No:-

NRP Box No:-

Organ	Placed at time of retrieval (Y/N)	Retrieved (Y/N)	Accepting Centre
Liver			
R. Kidney			
L. Kidney			
Pancreas			
Lungs			
Heart			

Edinburgh Transplant Centre ©.

Blood Sampling

0 Hr

- Blood Gas
- U&Es/LFTs (5mls)
- Measure & sample bile
- Blood Cultures (2 X 10mls)
- Piccolo Sample (0.1ml)

NRP Sampling

- Blood tubes (3 X 5ml)
- Liver Biopsy

40mls

½ Hr

- Blood Gas
- Piccolo Sample (0.1ml)

5mls

1 Hr

- Blood Gas
- Measure & sample bile
- Piccolo Sample (0.1ml)

NRP Sampling

- Blood tubes (3 X 5ml)

20mls

1½ Hr

- Blood Gas
- Piccolo Sample (0.1ml)

5mls

2 Hr

- Blood Gas
- U&Es/LFTs (5mls)
- Measure & sample bile
- Blood Cultures (2 X 10mls)
- Piccolo Sample (0.1ml)

NRP Sampling

- Blood tubes (3 X 5ml)
- Liver Biopsy
- R. Kidney Biopsy
- L. Kidney Biopsy
- Urine sample

40mls

Edinburgh Transplant Centre © October 2021, Review October 2022

edinburgh transplant centre

Blood Sampling Checklist

0 Hr

- Blood Gas ☐
- Piccolo Sample ☐ (0.1ml)
- U&Es/LFTs (5mls) ☐
- Blood Cultures ☐ (2 X 10mls)

NRP Sampling

Taken Spun

- Blood tubes ☐ ☐ (3 X 5ml)
- Liver Biopsy ☐

40mls

½ Hr

- Blood Gas ☐
- Piccolo Sample ☐ (0.1ml)

5mls

1 Hr

- Blood Gas ☐
- Piccolo Sample ☐ (0.1ml)

NRP Sampling

Taken Spun

- Blood tubes ☐ ☐ (3 X 5ml)

20mls

1½ Hr

- Blood Gas ☐
- Piccolo Sample ☐ (0.1ml)

5mls

2 Hr

- Blood Gas ☐
- Piccolo Sample ☐
- U&Es/LFTs (5mls) ☐
- Blood Cultures ☐ (2 x 10mls)

NRP Sampling

Taken

Spun

- Blood tubes ☐ ☐ (3 X 5ml)
- Liver Biopsy ☐
- R. Kidney Biopsy ☐
- L. Kidney Biopsy ☐

40mls

Edinburgh Transplant Centre © October 2021. Review October 2022.

edinburgh transplant centre

System Setup

- Connect Cardiohelp and Heater Unit to the main AC power supply.
- Connect Air & Oxygen supply.
- Check venous probe is in the park position to allow calibration before use.
- Switch on Cardiohelp - it will go through a short self-test.
- Press the menu key d select thApp.
- Select MECC and confirm
- Activate the data record function. Press Menu File
- Check probe has been initialised successfully.
- If the probe has not initialised successfully. Place the probe back in the parking position.

  Press - the probe will calibrate again then .
- Lift up the blue protection frame by pressing both silver buttons and releasing the frame.
- Switch Cardiohelp into Global override for priming. Press and hold then touch global override on the touch screen changes to

Assembly

- Open the disposable pack. Securely close the Roberts clip on the accessory limb of the reservoir. Remove circuit and the sash pack and hang up the sash pack on the IV stand.
- Connect the oxygenator securely to the Cardiohelp drive.
- Connect the reservoir to the reservoir holder and secure.
- Place tubing clamp on tubing below reservoir.
- Fix the leukocyte filter to the holder. Connect the filter purge line to the reservoir and place tubing clamp pre-leucocyte filter.
- Connect Heater Unit water lines to the oxygenator, switch on heater unit and set temp to 38 degrees.
- Attach pressure lines (pig tail lines) to transducer holders:-

  - Connect pre- membrane pressure line (P Int./yellow line) to the luer on the back of the oxygenator (top left hand port).

  -Connect the post membrane (P Art./red line) to the luer under the red cap immediately after the oxygenator.
- Connect the 3 gang Manifold to the reservoir. Connect blue sampling line to the luer on the venous arm of the circuit (going into reservoir). Connect red sampling line to the luer port on the Y connector pre-leucocyte filter. Turn 3-way taps on sampling ports to off position.
- Connect the green gas tubing to the filter and the gas blender. Turn on oxygen to 70% oxygen 2.0 litre flow.
- Connect flow sensor to the arterial line- the arrow should point to the patient (in the direction of flow).
- Connect cold perfusion line adaptor to fast flow giving set from retrieval pack and prime with cold green UW ensuring no air in the line. Attach cold perfusion line to the Y connector luer port below the leucocyte filter.

Edinburgh Transplant Centre ©

Priming and De-airing

- Remove the larger (rubber) white cap from the top (centre) of the reservoir and connect the quick prime line.
- Close clamps on the priming line and attach priming solution bags.
- Remove the yellow vent caps on the reservoir.
- Begin gravity prime by allowing the prime fluid to fill reservoir with a minimum of 2000 ml, then release the tubing clamp post reservoir.
- When the fluid reaches the arterial line, bleed the purging port (white cap) at the upper corner of the oxygenator.
- Attach the yellow cap to open luer port on back of oxygenator next to P.Int connection.
- To initiate the active priming increase pump to 2500 RPM by turning the rotary knob.
- Once leucocyte filter gets primed retrogradely, release the pre-filter clamp and put this clamp on the leucocyte filter bypass line.
- Remove the filter from the holder. Invert and tap to ensure proper de-airing. Replace filter into holder and remove blue cap to release air. If required pinch the arterial line (post filter) to ensure proper de-airing of the top of the filter. Any further bubbles can be removed via the purge line. Clamp purge line once this is done.
- Prime both pre. and post- membrane pressure lines by releasing white caps until line is completely primed and de-aired. Zero the external pressure sensors by opening three way taps to air.
  - Press interventions key 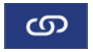
  - Press 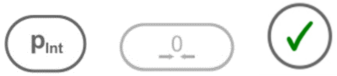

    Press 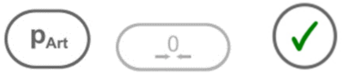

Edinburgh Transplant Centre ©
Version 6. January 2021. Review January 2022.

- Reset arterial bubble sensor. Press Art RESET
- Close the sampling manifold by turning red (arterial) and white sampling port three way taps off.
- Place the venous probe on the circuit venous probe connector.
- Ensure the circuit is warm, by monitoring the venous temperature on the Cardiohelp Screen.
- Press Blood Parameters and ensure "Tven" is between 35.5°C - 37.5°C T_{Ven} °C 36.5
- Stop Pump by reducing RPM to "0".
- Calibrate the flow sensor. Clamp either side of flow probe with tubing clamps.

  Press flow key V̇ 0 Remove clamps once zeroed and re-start pump to 2000 RPM.
- Ensure all prescribed drugs have been added as per protocol. Remove all clamps to allow perfusate to circulate, maintaining temperature until asystole.

At Asystole

- Deactivate Global Override mode. Press and hold S then touch global override on the touch screen Changes to S
- Clamp arterial line (after flow sensor).
- Clamp venous line.
- Clamp line pre-leucocyte filter.
- Pass the sash pack to the scrub nurse and secure in a sterile basin.

Going on NRP

- Release venous clamp ensuring adequate venous drainage.
- Release arterial clamp.
- Adjust RPM to achieve blood flow of 2-3 litres/minute.
- After circulating blood has mixed with priming fluid with added heparin (approx. 2 cycles) release tubing clamp from pre-leucocyte filter and place on leucocyte filter by-pass line.

End of NRP

- Prior to conversion to cold perfusion, clamp line below leucocyte filter and turn pump down to 0 and open the Roberts clip on the cold perfusion line.
- After completing the NRP case, STOP the data recording function. Press Menu 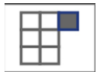 File 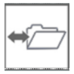 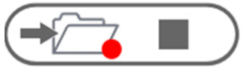
- Place the USB stick provided in the USB slot in front of the Cardiohelp.
- Open Data Recording menu 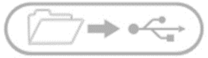 and click Data Transfer button.
- Once the data transfer is complete press 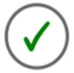 Remove USB Stick and replace black protection cap.

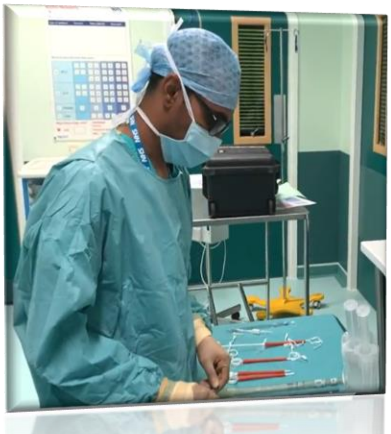

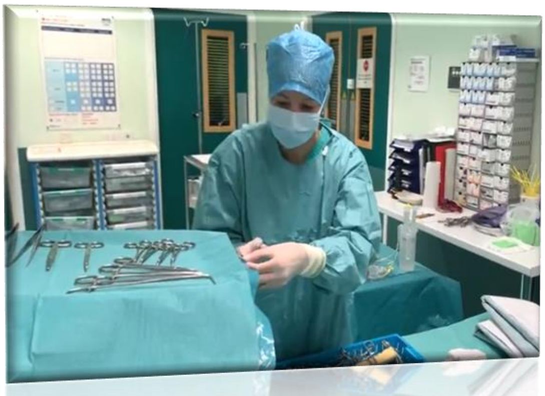

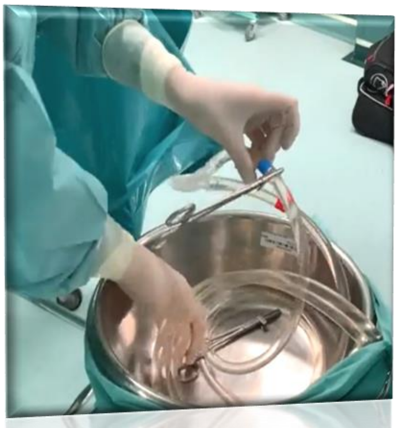

DCD ANRP SCRUB PRACTITIONER AND ORGAN PRESERVATION PRACTITIONER GUIDE

This guide is intended to provide the Scrub Practitioner (SP) and Organ Preservation Practitioner (OPP) with a basic overview of the DCD Abdominal NRP retrieval and cannulation procedure. It should be used in combination with practical sessions and the current UK National NRP Protocol.

Edinburgh Transplant Centre ©.
Version 2 March 2022. Review March 2023

Contents

1		Introduction to the Team	2
2		DCD NRP Equipment	
	2.1	Overview of Equipment	2
	2.2	Surgical Extras	3
	2.3	NRP Machine Set Up	4
	2.4	Cannulation Equipment	5
	2.5	Controlling The Thoracic Aorta and Blood Flow Above the Diaphragm	5
3		Scrub Practitioner (SP) Guide	
	3.1	Arrival at Donor Hospital and Initial Set Up	7
	3.2	At Asystole	8
	3.3	Transfer of Donor to Theatre and Laparotomy	9
	3.4	Cannulation	9
	3.4.1	Aortic Cannulation	10
	3.4.2	Venous Cannulation	11
	3.4.3	Cannulation Overview	12
	3.5	Sternotomy	12
	3.6	Establishing NRP, Haemostasis and Surgical Dissection	13
	3.7	Conversion to Cold Perfusion	13
4		Organ Preservation Practitioner (OPP) Guide	
	4.1	Arrival at the Donor Hospital and Initial Set Up	14
5		End of NRP and Start of Cold Perfusion	15
6		Summary	15

Edinburgh Transplant Centre ©.
Version 2 March 2022. Review March 2023.

1. Introduction to the Team

- The Retrieval Team for ANRP is made up of a Scrub Practitioner (SP), an Organ Preservation Practitioner (OPP) performing cold perfusion, an Advanced Perfusion and Organ Preservation Specialist (APOPS) who will operate the NRP machine, and 2 Surgeons, at least one of whom has been accredited as competent to perform abdominal NRP.

- To ensure effective teamwork, all team members should have insight into each other's roles and responsibilities.

2. DCD NRP Equipment

2.1 Overview

- The SP should ensure that both the Retrieval Tray and the small square Supplementary Retrieval Tray are taken to all DCD NRP retrievals. They should also ensure there is a dedicated stainless steel basin available to house the sterile sash of the NRP circuit.

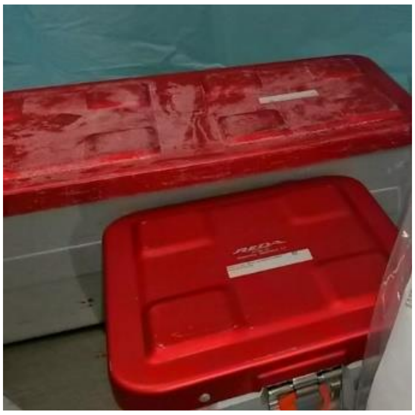

- The APOPS is responsible for checking the NRP bag and equipment, but it is important for SPs and OPPs to familiarise themselves with this equipment and especially with the surgical extras (packed in the NRP bag) which will be opened onto the surgical field.

Edinburgh Transplant Centre ©.
Version 2 March 2022. Review March 2023.

- There are 4 extra pieces of equipment for NRP in addition to a standard DCD
  - NRP Machine
  - Maquet Circuit
  - Piccolo Xpress Analyser
  - NRP bag

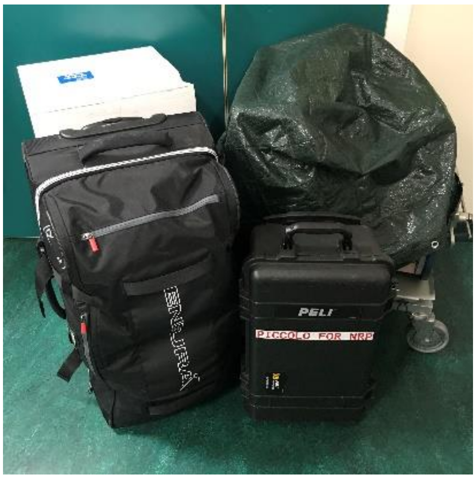

2.2 Surgical Extras

- The SP will take on the following surgical extras which are specific to DCD NRP.

Coda Balloon Catheter
Venous Cannula 1 x 29Fr 1 x 36Fr
3/0 Prolene 8522 x 2
50ml Catheter Tip Syringe x 2
50ml Luer Syringe x 2
5ml Syringe x 1
Filter Needle x 1
Irrigation Jet x 1
NaCl 0.9% 500ml x 1
Heparin 5000 units/5mls x 1 vial
Argyle Snuggers x 2 packs
Aortic Root Cannula
Medtronic Cardiac Tourniquet kit
Selection of T Tubes (9-10CH most commonly used)
30ml Luer syringe and 3 way tap

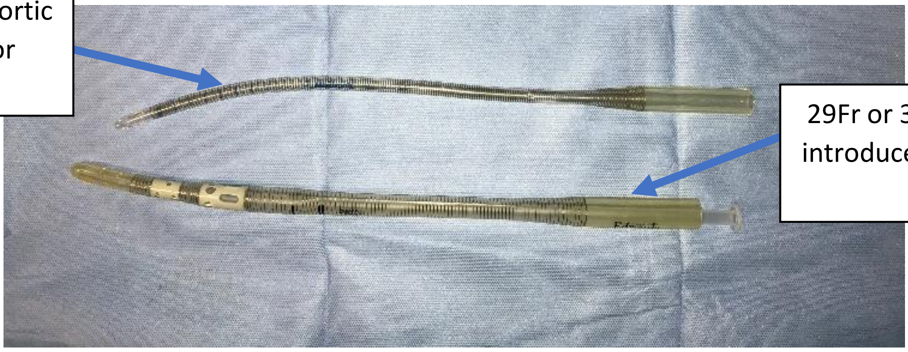

Edinburgh Transplant Centre ©.
Version 2 March 2022. Review March 2023.

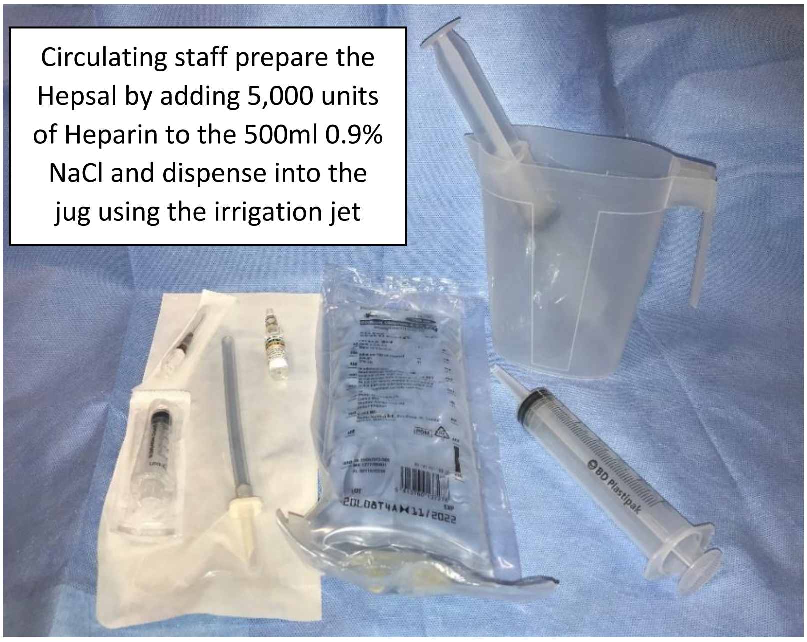

Circulating staff prepare the Hepsal by adding 5,000 units of Heparin to the 500ml 0.9% NaCl and dispense into the jug using the irrigation jet

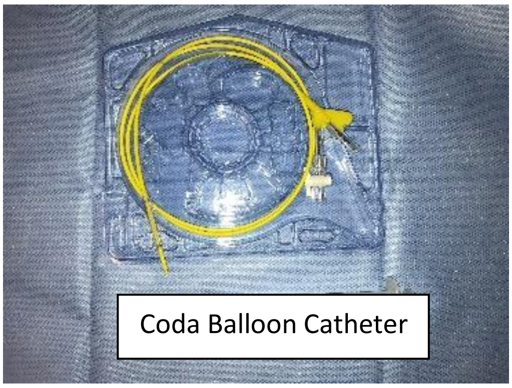

Coda Balloon Catheter

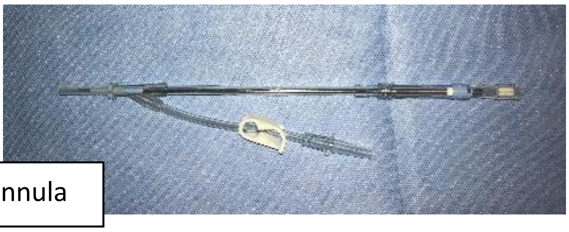

Aortic Root Cannula

2.3 NRP Machine Set Up

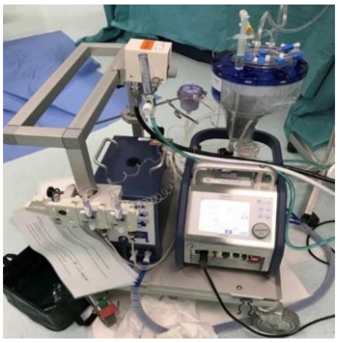

- The APOPS sets up the NRP machine at the foot of the table on what will be the right hand side of the donor. The disposable circuit is mounted and connected to the water heater, Cardiohelp and oxygen and air supply.

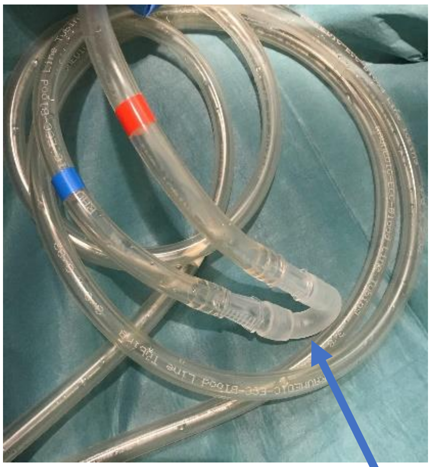

- The ‘sash’ of the NRP circuit is the sterile part of the circuit which is taken onto the sterile field by the SP.
- It consists of the arterial and venous limbs of the circuit which will bring oxygenated blood from the pump and then return deoxygenated blood to the reservoir. It is clearly marked with red and blue markings to identify the arterial and venous limbs.
- The connectors at the ends these limbs are different sizes and are specifically designed to fit **EITHER** the aortic **OR** the venous cannula.
- There is a detachable U-shaped connector which connects the two limbs of the circuit. This maintains the closed loop of the circuit to allow priming. It is not removed until just before cannulation is performed, at which time there are clamps placed on both limbs to ensure no air enters the circuit.

Edinburgh Transplant Centre ©.

2.4 Cannulation Equipment

- Cannulation can be either in the groin using the femoral vessels or in the abdomen using the abdominal aorta and IVC. We will focus here on the abdominal technique which is most frequently used by the ETC surgical team.

- A standard aortic cannula is used to cannulate the abdominal aorta, size is donor dependent but most frequently 22-26Fr.

- A three stage venous cannula, either 29Fr or 36Fr, is used to cannulate the IVC. The venous cannula is much larger than the aortic cannula and has an introducer which must be removed before use. It should be noted that the 29Fr cannula has an integral hard shell connector which must be removed to allow connection to the sash.

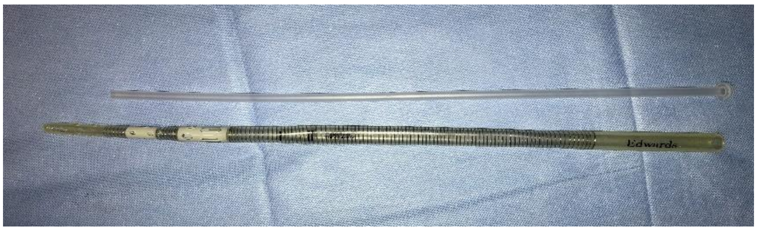

2.5 Controlling the Thoracic Aorta and Blood Flow Above the Diaphragm

- A large aortic clamp is used to clamp the thoracic aorta.

- The Aortic Root Cannula is used to monitor pressure and flow in the aorta and guard against intracranial arterial supply which could potentially occur if the thoracic aortic clamp was not placed correctly. It is inserted into the ascending thoracic aorta and is left open to atmosphere. The protective tip should be removed just prior to use and the introducer should be handled with care once it has been removed by the Surgeon.

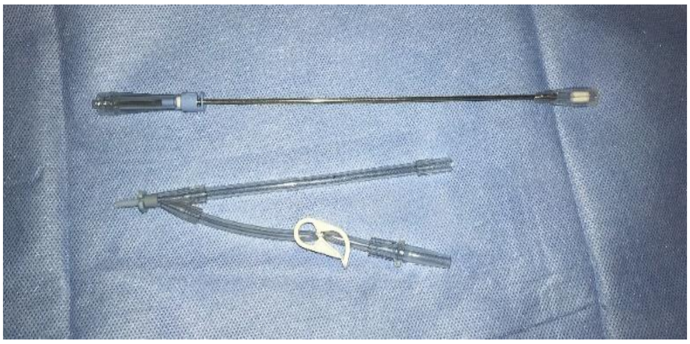

- Further clamps may be used to occlude the IVC and/or SVC once NRP is established. These clamps are obtained from the Supplementary Retrieval Tray.

Edinburgh Transplant Centre ©.
Version 2 March 2022. Review March 2023.

- The Coda Balloon Catheter can also used to occlude the aorta. It is inflated using 30mls Hepsal. Once inflated, the tap is rotated 90 degrees.

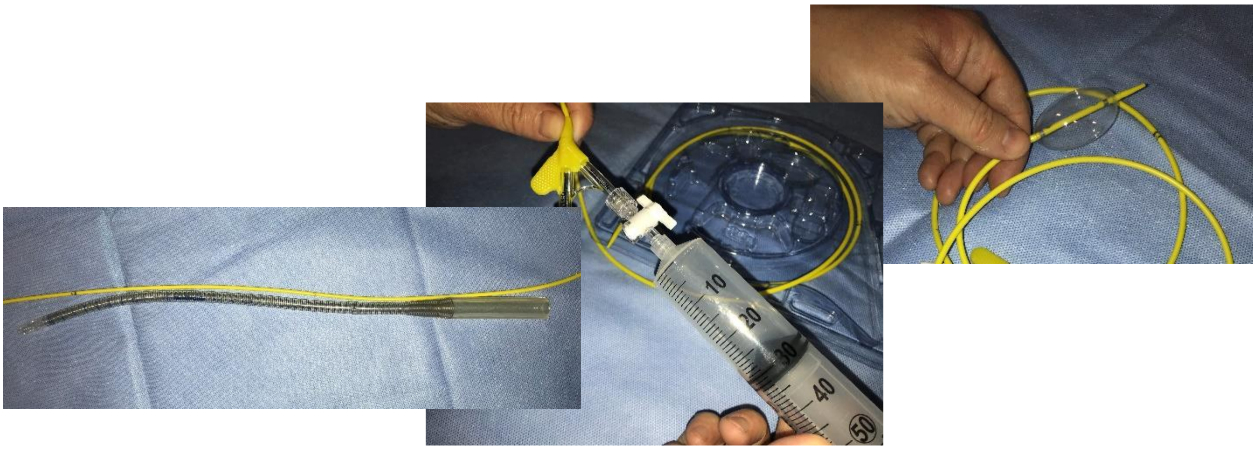

- Potential blood flow above the diaphragm can also be monitored via the distal port on the Coda Balloon Catheter. Left open to air, no blood should flow from the port.

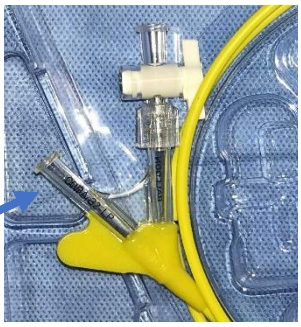

- This is the basic overview of the extra equipment required for NRP. The lead Surgeon will talk through the cannulation process with the SP. As there are various options available to occlude blood flow above the diaphragm, the chosen method should be made clear in advance and the SP should use this opportunity to clarify any queries they may have.

Edinburgh Transplant Centre ©.
Version 2 March 2022. Review March 2023.

3. Scrub Practitioner Guide

3.1 Arrival at Donor Hospital and Initial Set Up

- On arrival at the donor hospital the SP should set up their scrub equipment promptly, opening both Retrieval trays. However, it is important to remember that, unlike a standard DCD, diathermy will be required as circulation will be returned to the abdominal organs during NRP and haemostasis must be maintained during the 2 hours of NRP.

- It may be the case that the venous cannula and Coda Aortic Balloon Cannula (if requested) are not opened until asystole, for reasons of cost, however these should be clearly identified and accessible to any circulating staff with clear instructions on when to be opened.

- The T-tube will be selected when required, once NRP is established, the size will be donor dependent. A selection of sizes should be available.

- The SP should hand off a Fast Flow Giving Set to the OPP so that the cold perfusion line can be primed ready for connection to the NRP circuit. It is essential that this is done promptly as the NRP machine set up cannot be completed until the cold perfusion line is primed and connected.

- The Hepsal will be prepared and decanted into the jug using the irrigation jet and both catheter tipped syringes should be available for use.

- Fully drape a basin stand to receive the sash and place 2 tubing clamps and a towel clip in the basin.

- The lead Surgeon will go over the steps from knife to skin, clearly identifying the following:

    - the required size of aortic and venous cannula

    - which vascular clamps might be required to occlude the SVC and IVC, once NRP is established

    - if the Coda Balloon Catheter will be used in addition to the Aortic Root Cannula

- **The SP should not hesitate to clarify any points with the Surgeon at this stage.**

Edinburgh Transplant Centre ©.

3.2 At Asystole

- The APOPS will open the sash cassette and the SP should carefully remove the sash from the sterile packaging and secure in the basin with the towel clip. Care should be taken as the tubing is non-sterile below the white foam guard.

The sash limbs are non sterile beyond this point

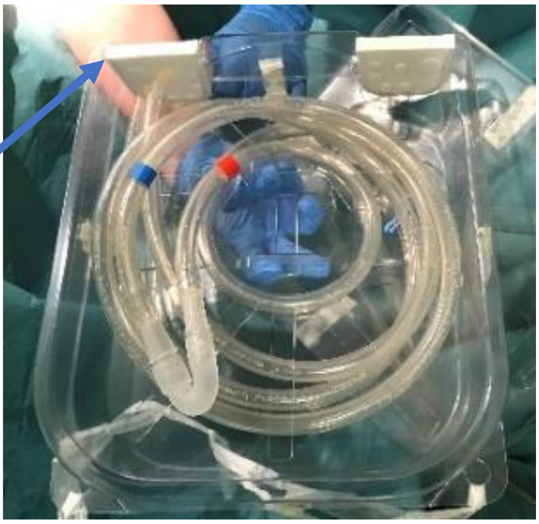

- Although the sash is usually opened at asystole, this may be done earlier if it is deemed necessary to inspect the security of the u-shaped connector. The SP should then cover the basin and contents with a drape to maintain sterility and allow the APOPS to safely move the basin stand.

- At asystole, the tubing clamps should be placed on both the arterial and venous limbs of the sash, just beyond the red and blue markers at around 10 cm from the end of each limb.

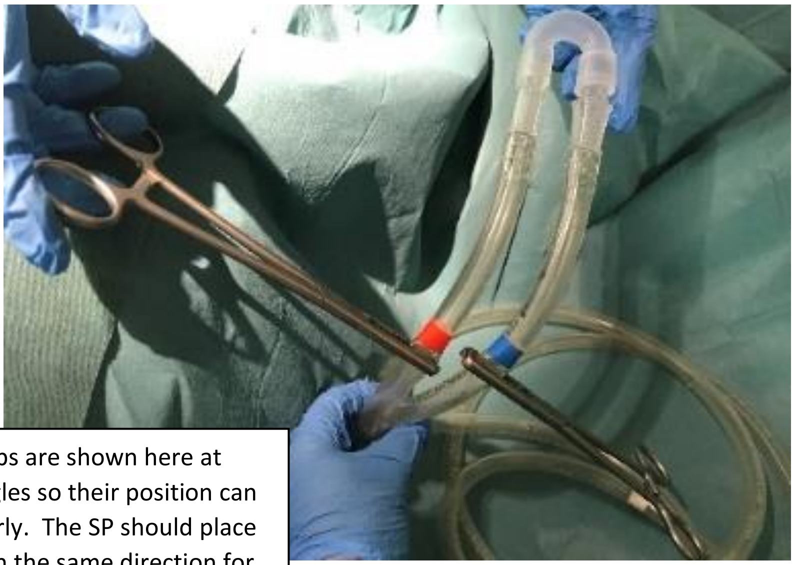

The clamps are shown here at opposite angles so their position can be seen clearly. The SP should place the clamps in the same direction for ease of attachment and removal.

- **THESE CLAMPS REMAIN IN PLACE UNTIL REMOVED BY THE LEAD SURGEON.**

3.3 Transfer of Donor to Theatre and Laparotomy

- The APOPS will keep the basin stand and sash safe during transfer of the donor and move it in reach of the SP once draping is complete.

- The SP should set up both suctions quickly as normal and secure to the drapes – it is worth taking the time to secure these as it is far more disruptive if they fall off the table and must be replaced. The diathermy is also set up, though remember it will not be required until NRP is established.

- The SP should lift the sash out of the basin in co-ordination with the APOPS who will take control of the towel clip and secure to the drapes below the foam guard. The SP should further secure the limbs on the table.

- The u-shaped connector will be removed prior to connection of the cannulas.

- The Surgeons will perform a rapid laparotomy.

3.4 Cannulation

- The SP should have heavy ties, Argyle Snuggers and Crilles available to assist with the cannulation process.

- The cannulas and Coda Balloon Catheter(if used) should be kept safe on the scrub trolley until required.

- The 50ml catheter tip syringes should be filled with Hepsal.

- The u-shaped connector should be removed from the sash.

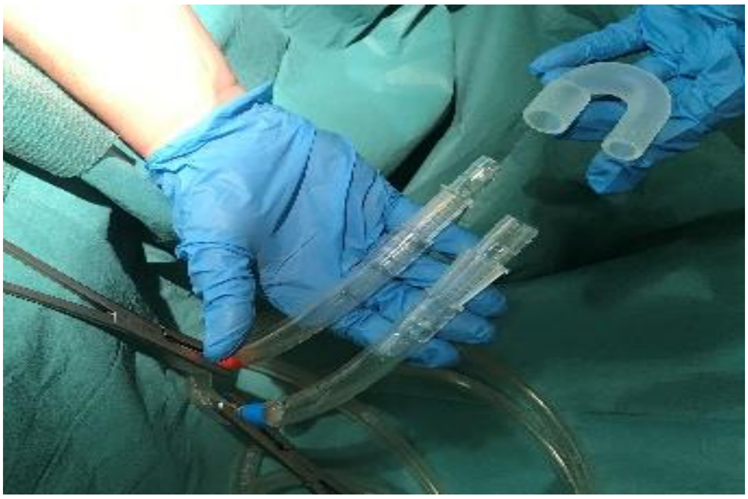

Edinburgh Transplant Centre ©.
Version 2 March 2022. Review March 2023.

3.4.1 Arterial Cannulation

- Blood flow to the lower limbs during NRP is prevented by clamping or ligating the distal aorta just above the point where iliac arteries bifurcate.

- The infra-renal abdominal aorta is then identified and slung with an Argyle snugger which will be used to secure the cannula once inserted.

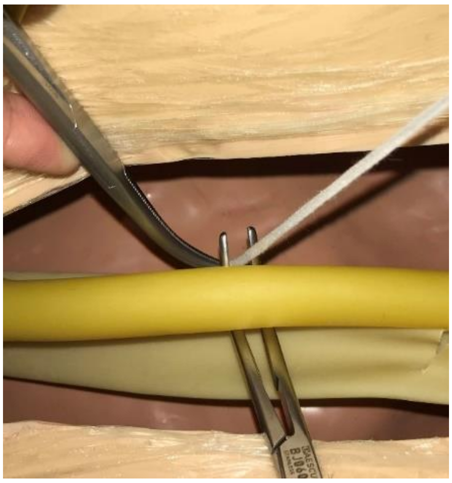

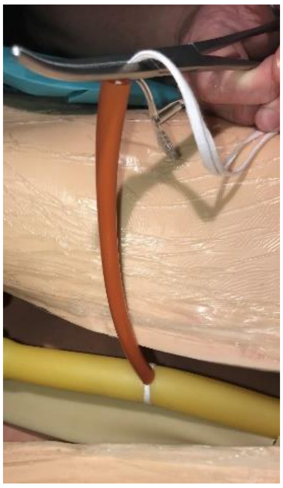

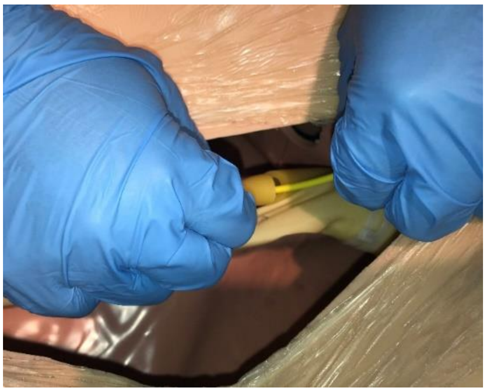

- If the Coda Balloon Catheter is being used this will be inserted into the aorta first. The Surgeons will make an incision in the aorta and feed the Coda Balloon Catheter into place.
- The luer lock 50ml syringe containing 30ml Hepsal should already be attached to the end of the catheter and the SP will be directed to inflate the balloon.
- The tap is rotated 90° to keep the balloon inflated.

- The Surgeon will then insert the aortic cannula, back flow of blood is controlled by placing a tubing clamp near to the end of the cannula. The cannula is secured in place with snuggers.

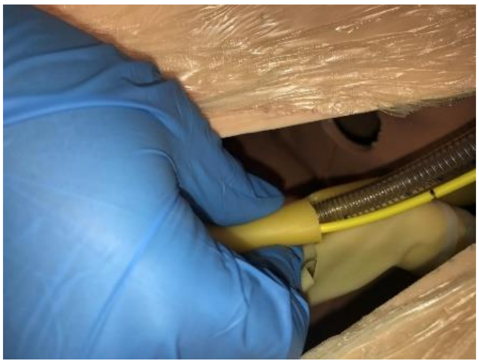

- If the balloon is not being used, the Surgeon will go directly to insertion of the aortic cannula.

Edinburgh Transplant Centre ©.
Version 2 March 2022. Review March 2023.

- To connect the aortic cannula to the arterial limb of the circuit, the Surgeon will bring the two ends close together, holding them vertically to encourage air bubbles to escape.

- The Surgeon allows blood to flow by releasing the tubing clamp on the aortic cannula and the SP and/or Assistant Surgeon will fill the space in the end of the arterial limb with Hepsal.

- It is **essential** to eliminate air bubbles at this stage. If the SP spots any air bubbles that may have been missed, they should bring this to the attention of the Surgeons.

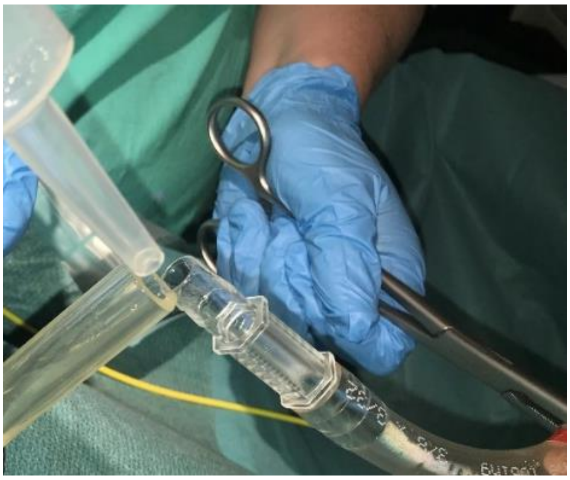

3.4.2 Venous Cannulation

- The Surgeon will clamp or ligate the distal vena cava.

- Two Crilles forceps should be available so the Assistant Surgeon can 'tent up' the IVC to allow an incision to be made safely

- The same steps as above are then taken to sling, incise, cannulate and connect the venous cannula to the venous limb of the circuit.

- The Surgeon may need to lower the venous cannula to a more horizontal position to encourage back flow of blood, however the venous limb of the circuit should remain upright.

- Once again, the SP will fill the empty space in the end of the venous limb with Hepsal so that the two ends can be connected.

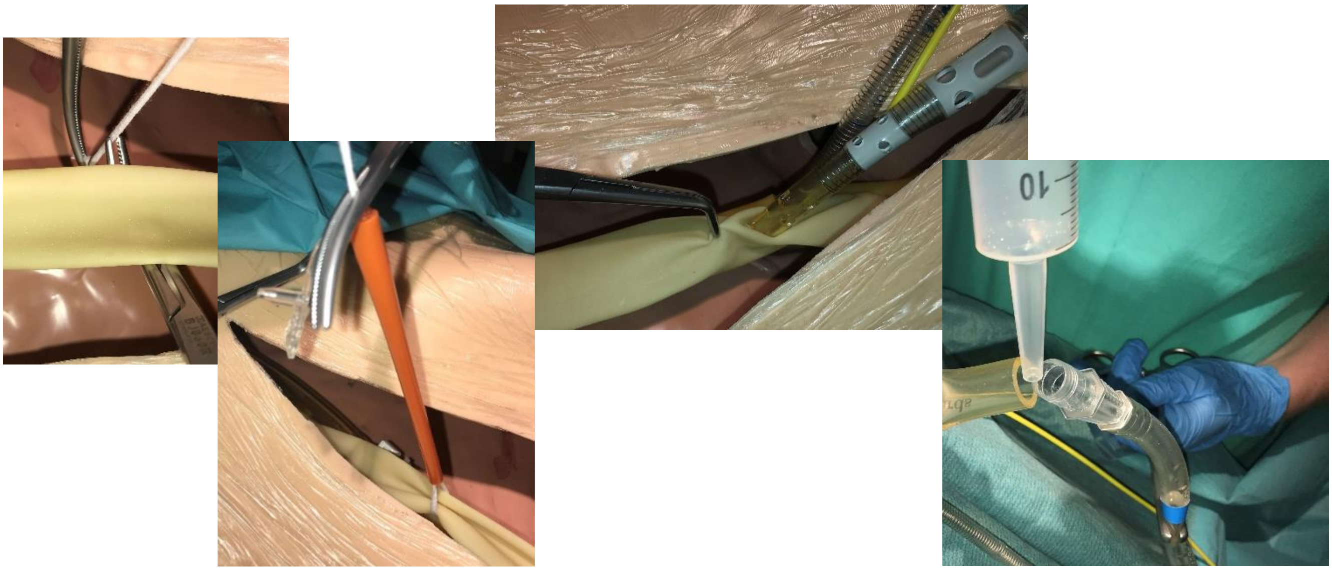

Edinburgh Transplant Centre ©.
Version 2 March 2022. Review March 2023.

3.4.3 Cannulation Overview

- The final positions of the arterial and venous cannula with snuggers and clamps in place are shown here.

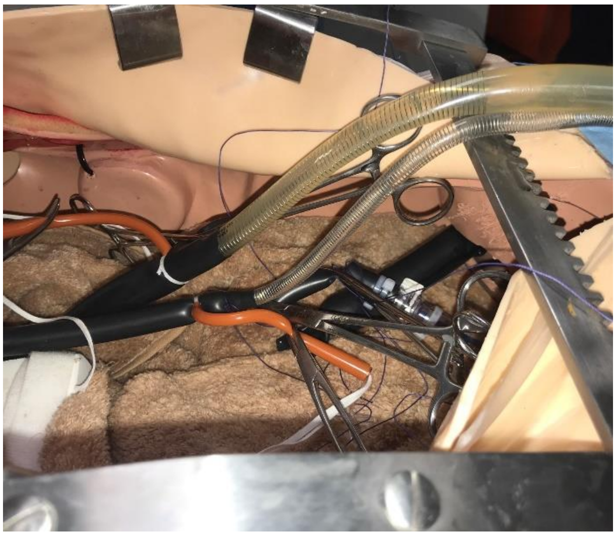

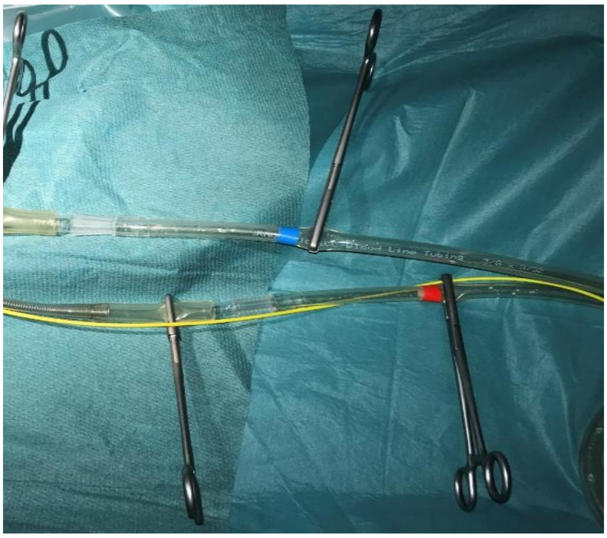

- **THE TUBING CLAMPS ON THE ARTERIAL AND VENOUS LIMBS OF THE CIRCUIT SHOULD ONLY BE REMOVED BY OR AT THE DIRECTION OF THE SURGEON.**

3.5 Sternotomy

- The Surgeon will carry out a rapid sternotomy using the Stryker saw.

- The large Debakey Aortic Clamp will be placed as a cross clamp on the thoracic aorta close to the diaphragm.

- The Aortic Root Cannula will be inserted into the aortic arch and left open to air. This will be secured using a 3/0 Prolene and a Medtonic Cardiac Tourniquet. The surgeon will return the cannula introducer to the SP – care must be taken with the sharp end of this.

Edinburgh Transplant Centre ©.

3.6 Establishing NRP, Haemostasis and Surgical Dissection

- The NRP pump should only be started once
    - Cannulation is successful and the circuit complete
    - The thoracic aorta is cross-clamped/occluded
    - The aorta above the diaphragm is vented

- At this point the lead Surgeon will coordinate with the APOPS to release clamps and start NRP.

- Once NRP is begun, the SP should be prepared to assist with haemostasis of the abdominal wound edges, sternotomy and retroperitoneal tissues. They should remain attentive to the security of the arterial and venous limbs throughout.

- NRP will continue for 2 hours. In the first hour the Surgeons will perform a full laparotomy and evaluate the organs.

- The gallbladder will be flushed as normal, and a t-tube inserted into the bile duct and secured in place. The SP will provide a 30ml syringe with barrel removed and a 3 way tap. This will be secured to the drapes and the t-tube will drain into it to allow for collection and sampling of bile.

- During the 2 hours of NRP, at least one person should remain scrubbed at the table.

3.7 Conversion to Cold Perfusion

- Conversion to cold perfusion follows at the end of the 2 hour period of NRP and the retrieval proceeds with the cold phase of a standard DBD retrieval.

- The SP should be aware that conversion to cold perfusion may need to happen sooner than 2 hours if there is excessive blood loss and should be prepared for this with plenty ice available.

Edinburgh Transplant Centre ©.

4 Organ Preservation Practitioner (OPP) Guide

4.1 Arrival at the Donor Hospital and Initial Set Up

- On arrival, the OPP will proceed as normal with basic set up of necessary equipment.

- The cold preservation fluids are prepared as for standard DCD. If there are issues establishing NRP or maintaining haemostasis or reservoir volumes during NRP, it may be necessary to convert to cold perfusion prior to the 2 hour mark. This means that the cold perfusion fluids must be available as for a standard DCD.

- The key task that the OPP must do to allow smooth progress of the NRP is to swiftly prime the cold perfusion line so that this can be connected to the NRP circuit and the APOPS can complete priming of the circuit. The fast flow giving set required for the cold perfusion is currently packed on the exterior of the scrub pack and there is always a second fast flow set packed separately.

- The APOPS will provide the OPP with a cold persuion adaptor to attach to the cold perfusion line which allows for connection to the NRP circuit. The OPP and APOPS should coordinate to prime the line to the tip of this adaptor and the APOPS will connect it to the appropriate port on the NRP circuit – this is where the cold perfusion will flow into the abdominal organs at the end of NRP.

- The APOPS and OPP must ensure that all clamps on the cold perfusion line are securely closed until cold perfusion commences.

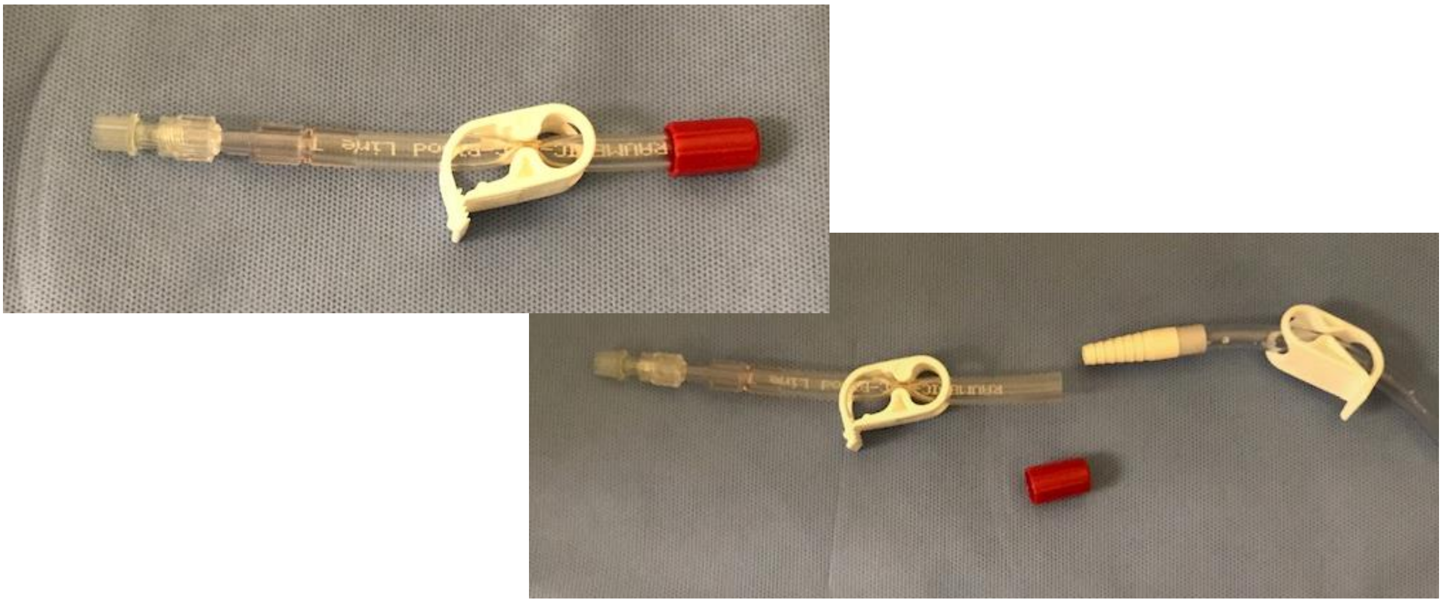

- During the 2 hours of NRP, the OPP can be a valuable support to the APOPS as they manage the NRP reservoir volume and perfusate sampling.

Edinburgh Transplant Centre ©.
Version 2 March 2022. Review March 2023.

5 End of NRP and Start of Cold Perfusion

- Successfully making the transition from NRP to cold perfusion requires careful teamwork. The APOPS, OPP, SP and surgeons should all be ready to perform their roles in a coordinated manner.
- The Surgeon and SP will clearly identify the venous limb of the circuit and prepare to clamp and cut.
- The OPP will hang the cold perfusion fluids and be ready to control the flow from the pressure bags to carry out cold perfusion.
- The APOPS will coordinate with the OPP to ensure all clamps on the cold perfusion line are released and take control of the final clamp on the cold perfusion adaptor immediately prior to the connection port on the NRP circuit.
- A yellow waste bin is placed to the right of the lead surgeon to receive the cut venous limb.
- When all members of the team are ready, take the following coordinated steps
    - the surgeon will clamp and cut the venous limb, directing the cut end towards the yellow bin.
    - The APOPS will place a clamp at an appropriate point on the arterial limb of the circuit and open the clamp on the cold perfusion adaptor
    - The OPP will begin the cold perfusion
    - The SP will provide ice slush to fill the abdomen
- The retrieval will now proceed cold phase dissection as in DBD.

6 Summary

- At all points during the NRP process, good communication between all members of the team is essential.
- Good teamwork is particularly important when commencing NRP and when converting from NRP to cold perfusion.
- All team members are encouraged to ask any questions needed to clarify their role or steps in the process at any time.

Edinburgh Transplant Centre ©.

<u>Femoral Cannulation for NRP</u>

Femoral cannulation is an alternative approach for establishing NRP. It is most commonly used either when there are concerns around ease of access to the abdomen, e.g. if the donor has had multiple laparotomies or has known abdominal adhesions or as a preferred approach when the cardiothoracic retrieval team are present.

1.1 Anatomy of the femoral triangle

- The cannulas will be inserted into the femoral artery and vein, shown here
- The right groin will be used as the default but it may be necessary in some circumstances to use the left groin e.g. if invasive lines are already present on the right
- The order of the vessels is the same on both sides – moving from the outside of the hip in to the midline– nerve, artery, vein.

N.A.V.Y.

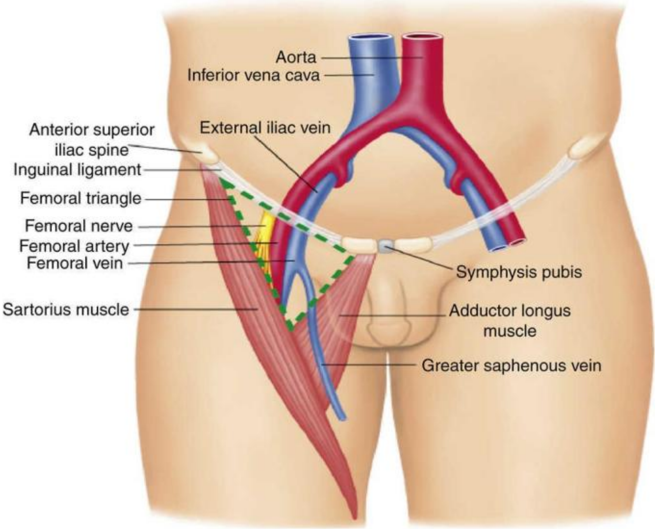

Dial Traspl. 2012;33:120–1

1.2 Cannulation Technique

- The groin should be prepped adequately and the drape positioned to expose the groin
- The Supplementary Retrieval Tray has 2 Travers Retractors to provide clear access to the groin
- The extra kit required will be provided by the APOPS - arterial and venous cannulas and vascular dilator kit.

- The cannulas used may vary in size and have a hard shell connector at the end like those on the arterial and venous limbs of the sash – as shown on the right.
- To connect the cannulas to the limbs of the sash, the hard connectors on the sash need to be removed. To do this, ensure tubing clamps are securely in place and gently manipulate the connectors until they separate from the sash tubing. The connectors should be retained in the sterile field, use a blue sterile marker pen to clearly identify the venous connector.

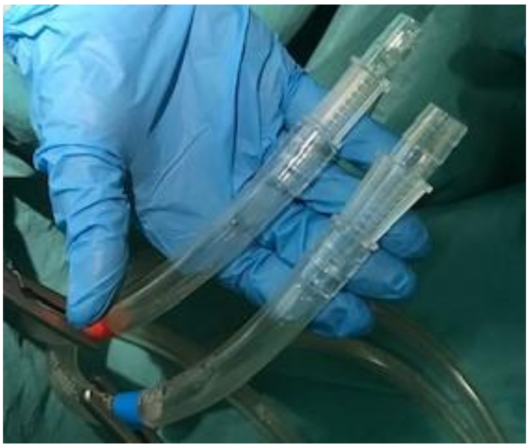

- See **1.3 Connecting Different Types of Cannulas** for further details.

- The **femoral artery** is cannulated first with the following steps (if CTR team are present, the vein will be first)
    - dissect the femoral artery
    - right angle followed by heavy tie to occlude the lower end of the artery
    - right angle and snuggers to secure the cannula
    - incision in artery
    - arterial cannula introduced – wet the cannula with HepSal
    - snug in place
    - connect the cannula to the arterial limb of sash, topping up with HepSal to ensure no bubbles

Edinburgh Transplant Centre ©.
Version 2 Aug 2021. Review Aug 2022

- The **femoral vein** is cannulated using the Seldinger technique – the same method the anaesthetists use to insert invasive access lines
- This requires the vascular dilator kit which contains a guidewire, dilators and a pink needle (the blade is not required)

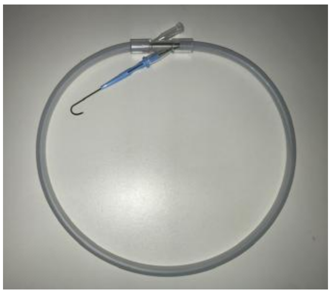

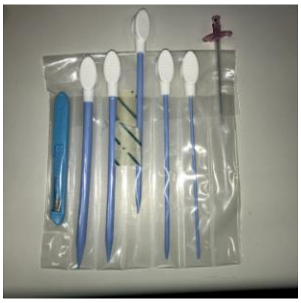

  - the anterior surface of the femoral vein is exposed
  - the pink needle from the dilator kit is inserted into the vein

  - pull the guidewire a short distance out of the plastic holder, advance the blue tip forward so that it covers the curly end of the guidewire. Present to the surgeon

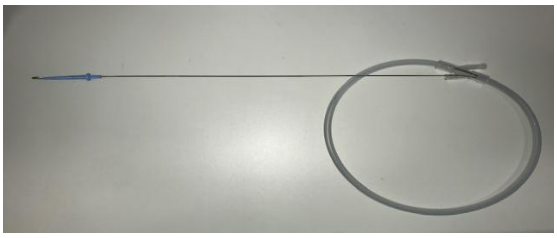

  - the blue tip connects to the needle and the guidewire is then fed through the needle into the vein. Once in place the needle and blue tip are slid backwards off the guidewire while the guidewire is held carefully in place

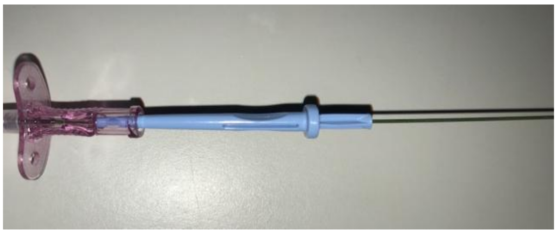

  - the selected dilator – usually the 16CH which is in the middle of the pack – is then slid over the end of the guidewire and advanced. Wet the dilator with HepSal

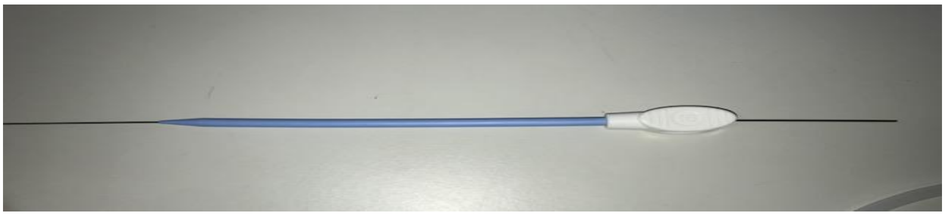

  - once the vein has been carefully dilated, the dilator is removed and the venous cannula is fed over the guidewire into the vein. Wet the cannula with HepSal. Alternatively, the surgeon may just use the tip of the cannula introducer to dilate the vein
  - connect the cannula to the venous limb of sash, topping up with HepSal to ensure no bubbles
  - The cannula can be secured with a silk stitch

- The surgeons will now perform a thoracotomy and place the usual clamps and DLP cannula in the chest before NRP begins.

- Once NRP is established the surgeons will begin the laparotomy and the retrieval will proceed as normal.

Edinburgh Transplant Centre ©.

1.3 Connecting Different Types of Cannulas

- The **standard femoral cannulas** currently in use are a 25Fr/38cm length venous and a 19Fr arterial. The connector ends of the cannulas are shown here with the introducers pulled back slightly to show the connectors clearly. Note that the arterial cannula has a side port which should be hand tightened on opening.

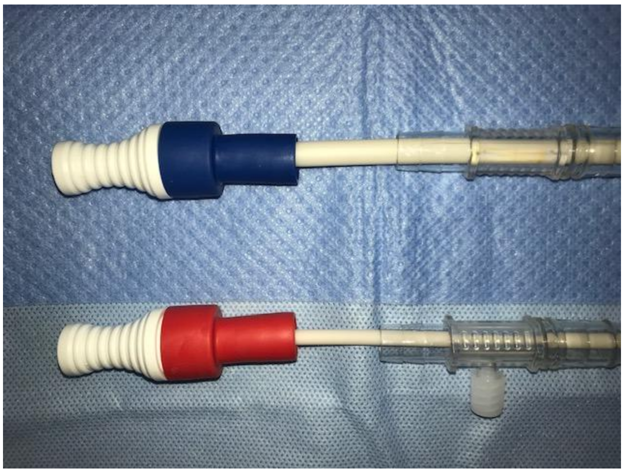

- The following sequence shows how the femoral cannulas can be easily connected to the sash

  - Take the sash from the sterile packaging

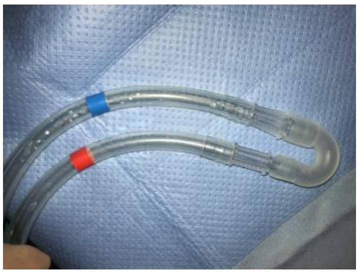

  - Apply the tubing clamps and carefully manipulate the sash connectors to remove them from the tubing

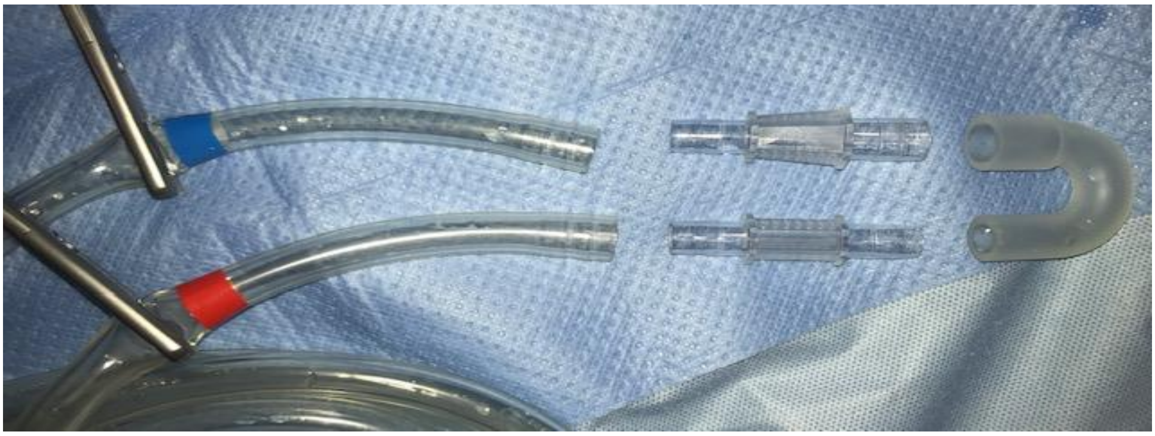

  - The sash connectors should be kept in case they are required to be used for abdominal cannulation should the femoral approach fail.
  - Mark the venous connector for easy identification

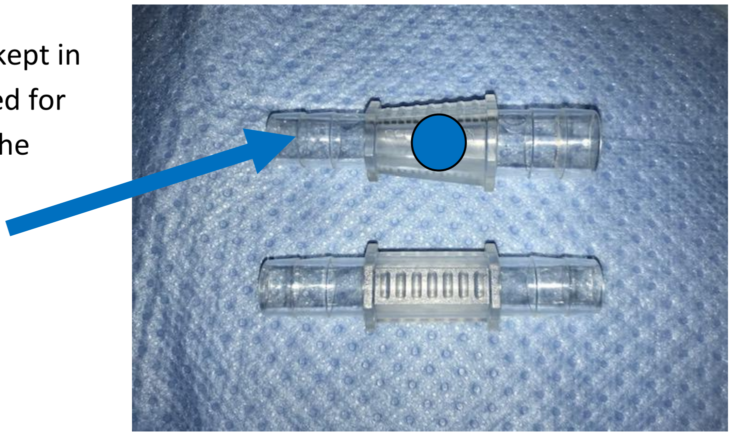

- The femoral cannulas will now attach directly onto the sash tubing as demonstrated here

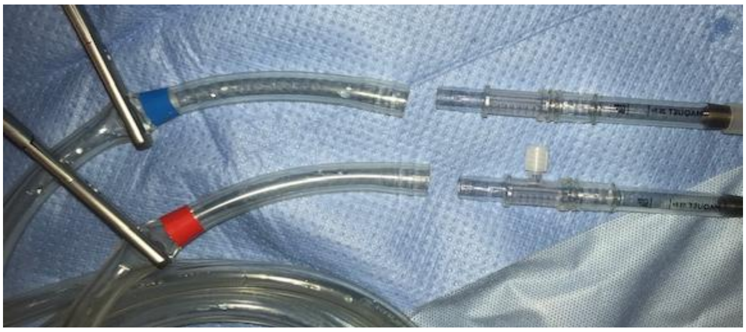

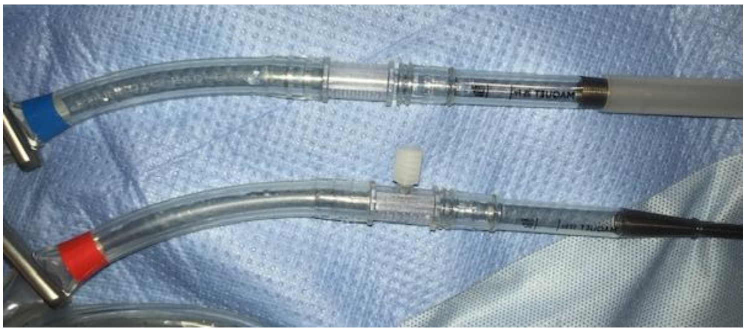

- If femoral cannulation is unsuccessful and the decision is made to use **abdominal cannulas** in the abdominal cannulation, the retained sash connectors should be reattached to the sash tubing to allow for connection as shown below.

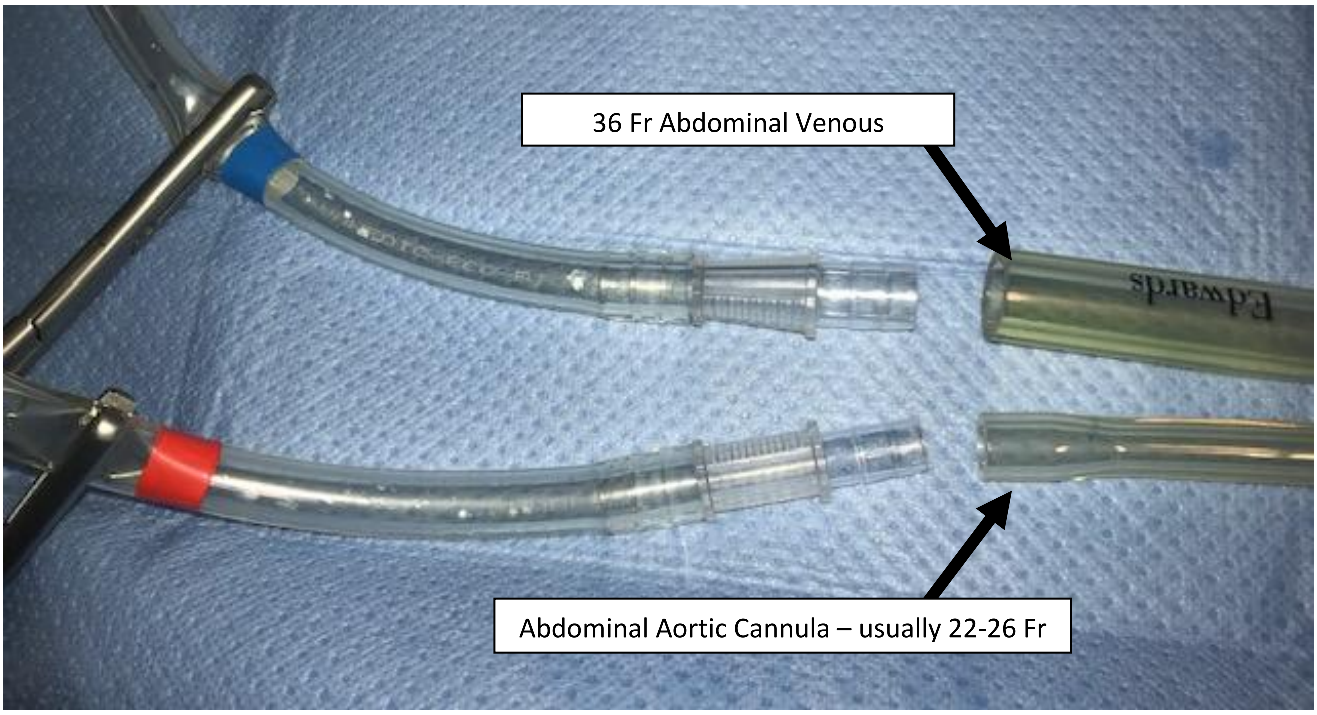

Edinburgh Transplant Centre ©.

- There is now a smaller abdominal venous cannula available – a 29Fr. This comes with a hard connector and this would need to be removed to allow for connection to the sash. The cannula is cut at the point shown – just after the 's' of 'Edwards'.

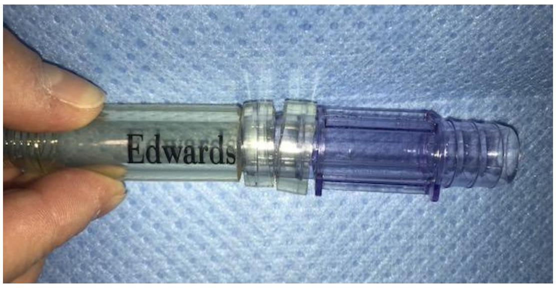

- This is the only cannula where the hard connector has to be cut off

- The 29Fr cannula will then connect easily to the hard connector of the sash. It is shown here with the larger 32Fr cannula for comparison

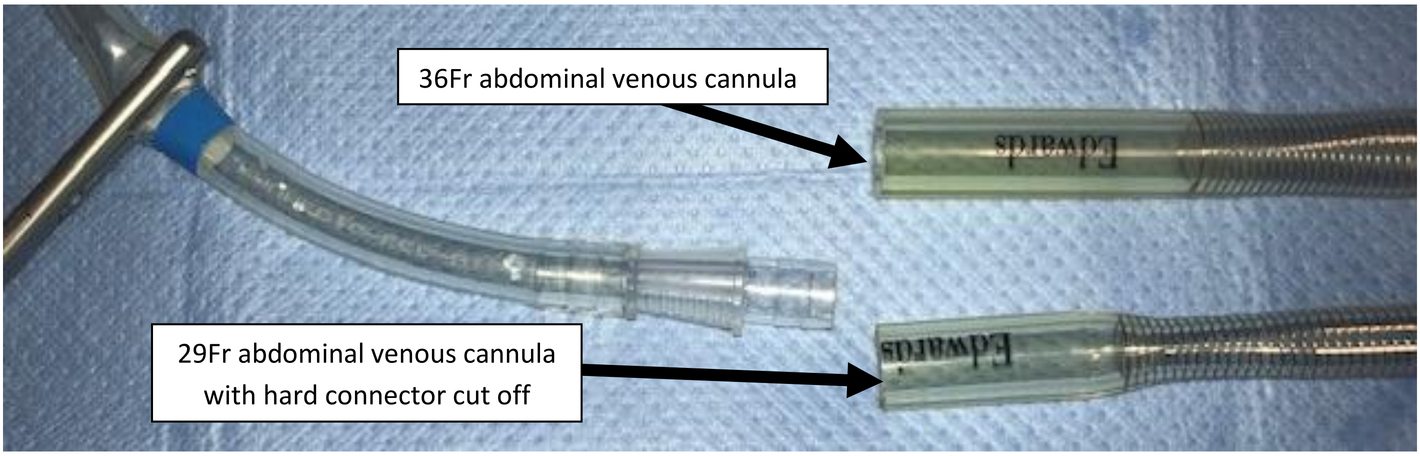

Edinburgh Transplant Centre ©.

CANNULA CONNECTION GUIDE

CONNECTS TO	Sash with hard connectors in place	Sash tubing – hard connectors removed
	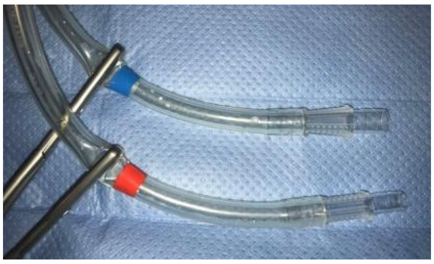	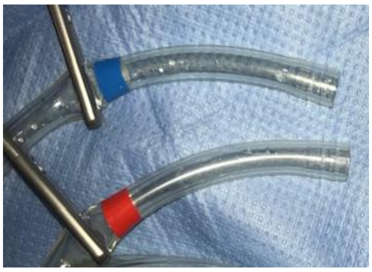
**Femoral Venous Cannula 25Fr**		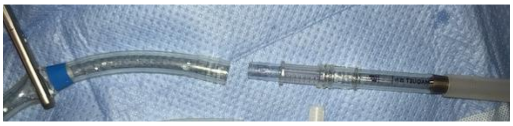
**Femoral Arterial Cannula 19Fr**		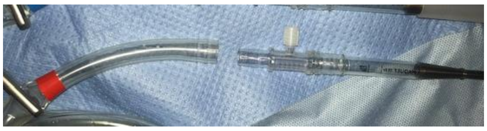
**Abdominal Venous Cannula 36Fr**	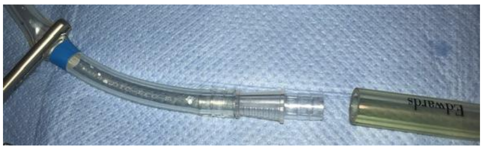	
**Abdominal Venous Cannula 29Fr – cut at the 's' of 'Edwards'**	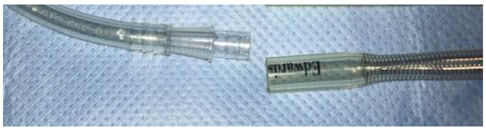	
**Abdominal Aortic Cannula any of 20-26Fr**	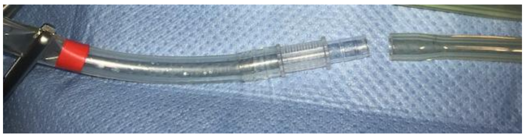	

Edinburgh Transplant Centre ©.
Version 2 Aug 2021. Review Aug 2022

Haemodynamic and biochemical goals

- Pump flow 2-3 litres/minute
- Temperature 35.5°C - 37.5°C
- Air / O2 to maintain a venous O2 saturation (SvO2) 60-80%
- Arterial pH 7.35-7.45
- Haematocrit > 20%
- Gas flow to maintain arterial pCO2 4.5 to 6.0 kPa.

Edinburgh Transplant Centre ©October 2020, Review October 2021

NRP TROUBLE SHOOTING

1. Frozen Screen

1. Hold down the safety button [1] and at the same time press the rotary knob [2] till you see the touchscreen calibration screen.

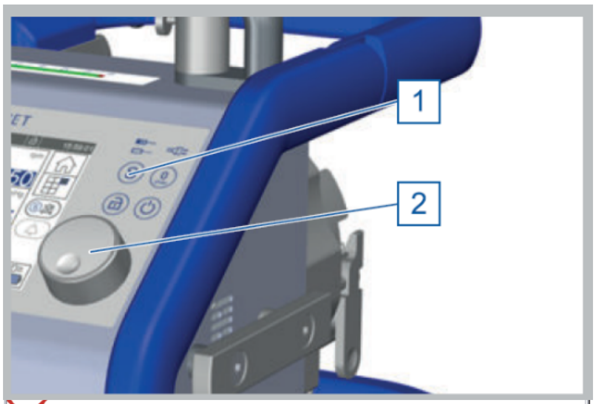

2. The touchscreen displays a red cross at various positions. Always touch exactly the centre of the Red Cross. CARDIOHELP checks the positions touched and calibrates the touchscreen accordingly.

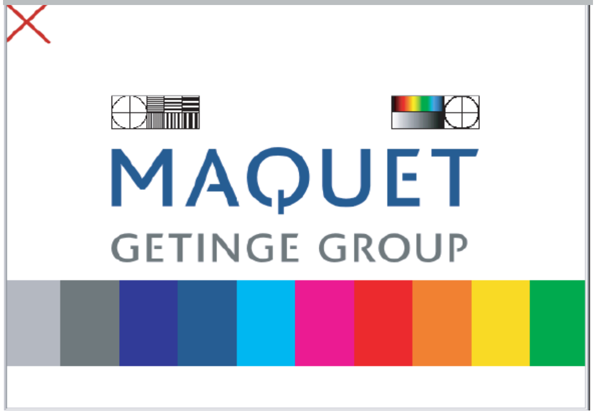

2. Increase of Volume in Reservoir

- ↑ in reservoir volume- if due to ↑ in venous return then ↑rpm to stabilise the blood level in the reservoir (the volume being given needs to equal the volume being returned).

- ↑ in reservoir volume & ↓ in pump flow & ↑ in RPM – Check Delta Pressure(ΔP) ΔP= P(int)-P(art)

    - If ΔP is unusually high i.e. double base value- this is likely to be due to clots either in oxygenator or filter → Add Heparin and check ACT → If ΔP is still seen increasing → convert to cold perfusion
    - If ΔP is not very high and both the Part and Pint are high –
        - Check for Kink in Arterial line
        - Check for Clamp in Arterial Line
        - Check for Arterial Cannula Position (Possible Cannula obstruction at the Tip of the Arterial Cannula)

Edinburgh Transplant Centre © October 2020, Review October 2021

NRP TROUBLE SHOOTING

3. Decrease of Volume in Reservoir

Causes of ↓ in volume in reservoir (venous return)	Action
**Airlock in venous line**	Move venous circuit tubing (up and down) to allow air to travel down into reservoir
**Kink in venous cannula or circuit tubing**	Release any obvious kinks/twists in cannula or circuit tubing
**Blocked venous cannula (pores/tip)**	Reposition cannula
**Excessive suction on the venous arm of circuit causing collapse of IVC**	Partially clamp venous line to reduce suction
**Hypovolaemia**	Add more volume<br>Check haemostasis

4. Empty Reservoir

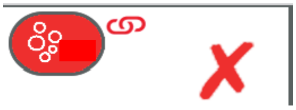

- Arterial Flow- Bubble sensor will detect air in circuit post-Reservoir when intervention is activated
- This will trigger high priority alarm
- Pump will stop automatically
- Add more volume if necessary
- Check if venous return is adequate.
- Reset flow sensor- press

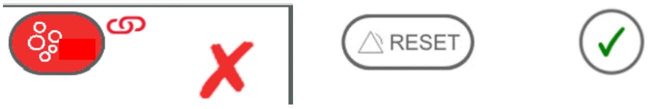

5. Air in Oxygenator

This should only happen if arterial bubble sensor intervention has not been activated.

- Clamp arterial line
- Stop pump
- Clamp venous line
- Run fluid into reservoir
- Aspirate air from the pre-membrane pigtail (using 50ml luer lock syringe)
- Remove any air from oxygenator purge port
- Start pump
- Remove yellow cap (back of oxygenator)
- Remove venous clamp
- Remove arterial clamp

Edinburgh Transplant Centre © October 2020, Review October 2021

edinburgh transplant centre

6. Air in Arterial Line Post Oxygenator

- Stop pump
- Clamp arterial line post filter
- Check if the Oxygenator and Pump is full of Air.
  - If Yes then follow the “Air in the Oxygenator- De-airing protocol” and then follow the next step.
  - If No then follow the next step.
- Move air along to leucocyte filter and evacuate air via blue de-air port and/or via purge line
- Restart pump
- Remove clamp

7. Broken Touchscreen

- Broken Touchscreen during usage of Cardiohelp

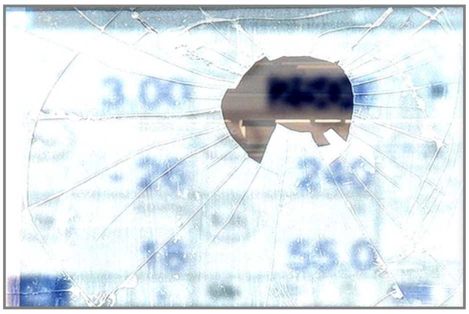

- Activate “Emergency Mode” by pressing the RED Button under the Rotary knob for 3 Beeps. (Till you hear a long beep)

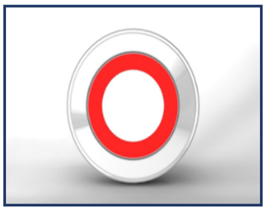

- Touchscreen functionality gets de-activated.

- Change RPM using the Rotary Knob and view RPM On the RPM display above the Touchscreen.

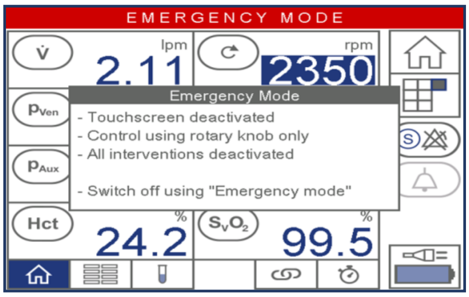

Supplement: Supplementary file 1 [file DataSheet1.PDF]
